# Gene conversion limits divergence of mammalian TLR1 and TLR6

**DOI:** 10.1186/1471-2148-7-148

**Published:** 2007-08-29

**Authors:** Egbert KO Kruithof, Nathalie Satta, Jia Wei Liu, Sylvie Dunoyer-Geindre, Richard J Fish

**Affiliations:** 1 Service of Angiology and Hemostasis, University Hospital of Geneva, CH-1211 Geneva, Switzerland

## Abstract

**Background:**

Toll-like receptors (TLR) recognize pathogen-associated molecular patterns and are important mediators of the innate immune system. TLR1 and TLR6 are paralogs and located in tandem on the same chromosome in mammals. They form heterodimers with TLR2 and bind lipopeptide components of gram-positive and gram-negative bacterial cell walls. To identify conserved stretches in TLR1 and TLR6, that may be important for their function, we compared their protein sequences in nine mammalian species(*Homo sapiens*, *Pan troglodytes*, *Macaca mulatta*, *Mus musculus*, *Rattus norvegicus*; *Erinaceus europaeus*, *Bos Taurus*, *Sus scrofa *and *Canis familiaris*).

**Results:**

The N-terminal sequences of the orthologous proteins showed greater similarity than corresponding paralog sequences. However, we identified a region of 300 amino acids towards the C-terminus of TLR1 and TLR6, where paralogs had a greater degree of sequence identity than orthologs. Preservation of DNA sequence identity of paralogs in this region was observed in all nine mammalian species investigated, and is due to independent gene conversion events. The regions having undergone gene conversion in each species are almost identical and encode the leucine-rich repeat motifs 16 to 19, the C-terminal cap motif, the transmembrane domain and most of the intracellular Toll/interleukin-1 receptor (TIR) domain.

**Conclusion:**

Our results show that, for a specific conserved region, divergence of TLR1 and TLR6 is limited by gene conversion, most likely because of the need for co-evolution with multiple intracellular and extracellular binding partners. Thus, gene conversion provides a mechanism for limiting the divergence of functional regions of protein paralogs, while allowing other domains to evolve diversified functions.

## Background

The innate immune response is important in the early stages of defense against bacterial or viral pathogens, and also for an efficient adaptive immune response [1]. Toll-like receptors (TLR) play a central role in the innate immune response. They recognize conserved pathogen-associated molecular patterns (PAMPs), such as lipopolysaccharides (LPS), lipopeptides, flagellins, dsRNA or CpG DNA motifs. PAMPs are specific for bacteria, viruses and protozoan parasites [2,3]. The human genome contains 10 different TLRs, and 12 mouse TLRs have been identified. TLRs are type 1 transmembrane proteins, with a large extracellular domain composed of up to 26 leucine-rich repeats (LRR) flanked by N-terminal and C-terminal cap motifs, a transmembrane domain and a cytoplasmic Toll/interleukin-1 receptor (TIR) domain. LRRs are motifs of 20–30 amino acids in length that fold into a horseshoe shape [4]. They occur in many different proteins and appear to provide a structural framework for the formation of protein-protein interactions [5]. In TLRs, the LRR motifs are involved in recognition of PAMPs and in hetero- or homo-dimerization. The TIR domain is highly conserved and interacts with adapter proteins such as MyD88 and TIRAP (also known as Mal) [6]. Ligand binding induces homo- or hetero-dimerization of TLRs, and subsequent recruitment of intracellular adapter proteins. Via intermediates such as TRAF6, IRAK family proteins and Ik-kinase family proteins, MAP-kinase signaling cascades and transcription factors including NF-kB family proteins are activated, which leads to increased expression of inflammatory cytokines, chemokines and adhesion molecules.

Six major TLR families have been identified in vertebrates; each TLR family recognizes one class of PAMPs [7]. Members of the TLR1 family, which include TLR1, TLR2 and TLR6, recognize lipopeptides, components of the cell wall of gram-positive and gram-negative bacteria. The unique particularity of this family is that TLR2 forms heterodimers with TLR1 or TLR6. These heterodimers recognize distinct molecular patterns of lipopeptides [8,9] and by exchanging domains between TLR1 and TLR6 the functional importance of LRR9-12, for discrimination between di-acyl and tri-acyl lipopeptides, was established [9].

Phylogenetic and chromosomal analysis suggests that TLR2 and a TLR1-like gene diverged from an ancestral gene early in vertebrate evolution [7]. The mammalian genes for TLR6, TLR1 and TLR10 are located consecutively on the same chromosome (4p14 in humans) and oriented in the same direction at intergenic distances of only 20 to 30 kilobases [7]. They result from successive tandem gene duplications of an ancestral gene that first gave rise to a TLR10 gene and a TLR1-like gene. Subsequent duplication of the TLR1-like gene resulted in the emergence of TLR1 and TLR6 [7]. The absence of separate TLR6 and TLR1 genes in the opossum and the platypus suggests that these paralogs arose by gene duplication after the divergence of placental mammals (eutherians) and marsupials (metatherians), and before the radiation of the eutherians (~100 My ago) ([7] and data presented here).

A previous report suggested the presence of coincidental evolution within the TLR1 family [7]. Furthermore, two recent studies showed that overall TIR domain sequences of TLR1 and TLR6 paralogs from three mammalian species clustered together, whereas the amino-terminal part of TLR1 or TLR6 clustered with their respective orthologs [10,11]. To better analyze this phenomenon, we compared the amino acid and DNA sequences of TLR1 and TLR6 in nine mammalian species to identify conserved regions that may be important for their functional activities. We found that sequences of the N-terminal domains, and the extreme C-terminal regions, are in agreement with divergence of TLR1 and TLR6 in a common ancestor of modern mammalian lineages. In contrast, for a 300 amino acid intervening sequence, paralogs in the same species were more similar than ortholog sequences between species, due to gene conversion events that occurred independently at the same positions in the ancestors of the mammals analyzed. This is a striking example of where gene conversion has enabled conservation of functional features of paralogs while other domains have evolved novel roles.

## Results

### Alignment of TLR1 and TLR6 protein sequences

**Figure 1:**
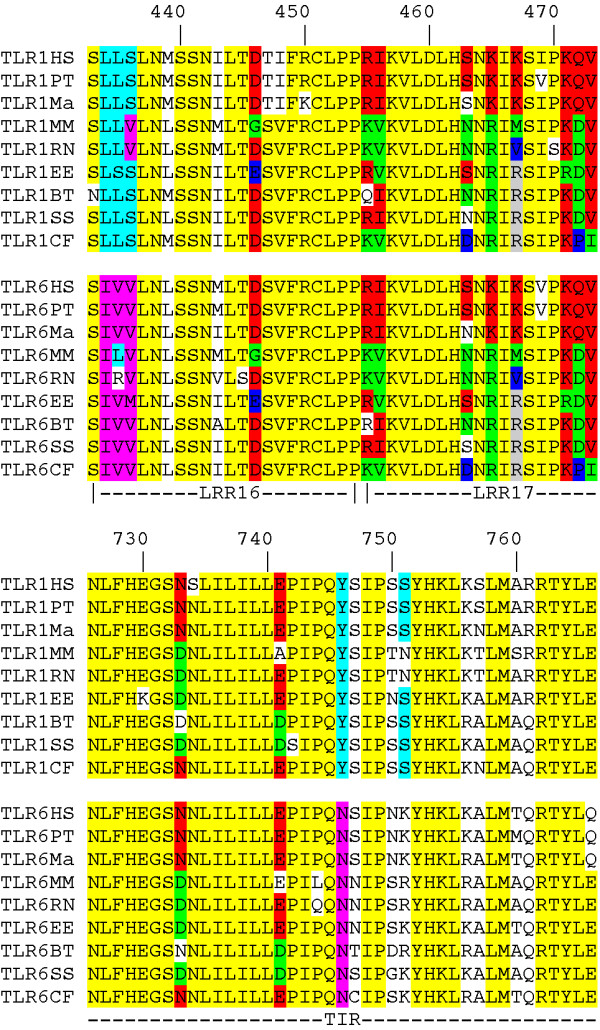
**Sequence identity patterns in mammalian TLR1 and TLR6**. Alignment of parts of the TLR1 and TLR6 amino acid sequences from nine mammalian species: *Homo sapiens *(HS), *Pan troglodytes *(PT), *Macaca mulatta *(Ma), *Mus musculus *(MM), *Rattus norvegicus *(RN), *Erinaceus europaeus *(EE), *Bos taurus *(BT), *Sus scrofa *(SS) and *Canis familiaris *(CF). Amino acids 433 to 473 and 726 to 766 are included as they include the junction sites of three patterns of sequence similarity. The three patterns of identity observed at a particular position are: A) ortholog sequences are more identical; B) paralog sequences are more identical; C) a high over all sequence identity. Color codes: Pattern A) in light blue (or pink): the same amino acid is conserved in 7 or more of 9 ortholog TLR1 (or TLR6) sequences and present in two or less TLR6 (or TLR1) sequences. Pattern B) in red, green, dark blue or grey: the sequences of the TLR1 and TLR6 paralogs are identical in at least seven species; these paralog pairs do not all have the same amino acid at this position. Pattern C) in yellow: the same amino acid is conserved in 14 or more of the 18 TLR1 and TLR6 sequences, patterns A or B do not apply. For the same analysis for the full length TLR1 and TLR6 proteins [see Additional Figure 1].

Conservation of amino acid sequence may reflect functional constraints on the evolution of proteins. As TLR1 and TLR6 both interact with lipopeptides, with TLR2 and with intracellular signal transducers such functional constraints are likely to be important in the evolution of these TLRs. Here, we conducted a search for conserved amino acids in TLR1 and TLR6, which may help to identify functionally important sequences. We aligned the amino acid sequences of TLR1 and TLR6 from nine mammalian species (human, chimpanzee, rhesus monkey, mouse, rat, hedgehog, cow, swine and dog). The complete alignment of TLR1 and TLR6 protein sequences, as well as the domain structure of TLR1 and TLR6, is given [see Additional file 1]. Overall, the average percentage identity for pairwise comparisons between sequences from the nine species was 75.5 ± 4.5 (SD) % for TLR1 and 75.2 ± 4.1% for TLR6. A close inspection of the sequence alignments revealed three patterns of sequence identity. In one pattern, an amino acid at a particular position of TLR1 was conserved among the orthologs and different from the amino acid at the same position in TLR6, and vice versa. In an alternative pattern, TLR1 and TLR6 from one species had the same amino acid, which was not necessarily conserved in other species at the same position. Visual inspection of the sequences revealed an abrupt transition from the first pattern to the second pattern in a region close to amino acid 436 [for numbering see Additional file 1] and a reversal to the first pattern near amino acid 745 (Figure 1 and [see Additional file 1]. The second pattern of sequence identity comprised part of the LRR16 motif, the LRR17, LRR18, and LRR19 motifs, the C-terminal cap motif, the transmembrane domain and most of the TIR domain. In a third pattern, TLR1 and TLR6 sequences had high overall similarity.

To determine whether these patterns were significant, we made a pairwise analysis for percentage sequence identity of the TLR1 and TLR6 sequences from one primate species (*Homo sapiens*), one rodent species (*Mus musculus*) and from hedgehog, cow, swine and dog. For the full length molecules, and for the regions 1–436 and 746-end, we observed that the average percentage sequence identity of the orthologous TLR1 or TLR6 sequences was significantly higher (p values ranging from < 0.01 to < 0.0001) than the average percentage sequence identity of the paralogous TLR1 and TLR6 sequence of the same species, whereas the reverse was true for the region 437–745 (p < 0.0001) [see Additional file 2].

### Analysis for gene conversion

**Figure 2:**
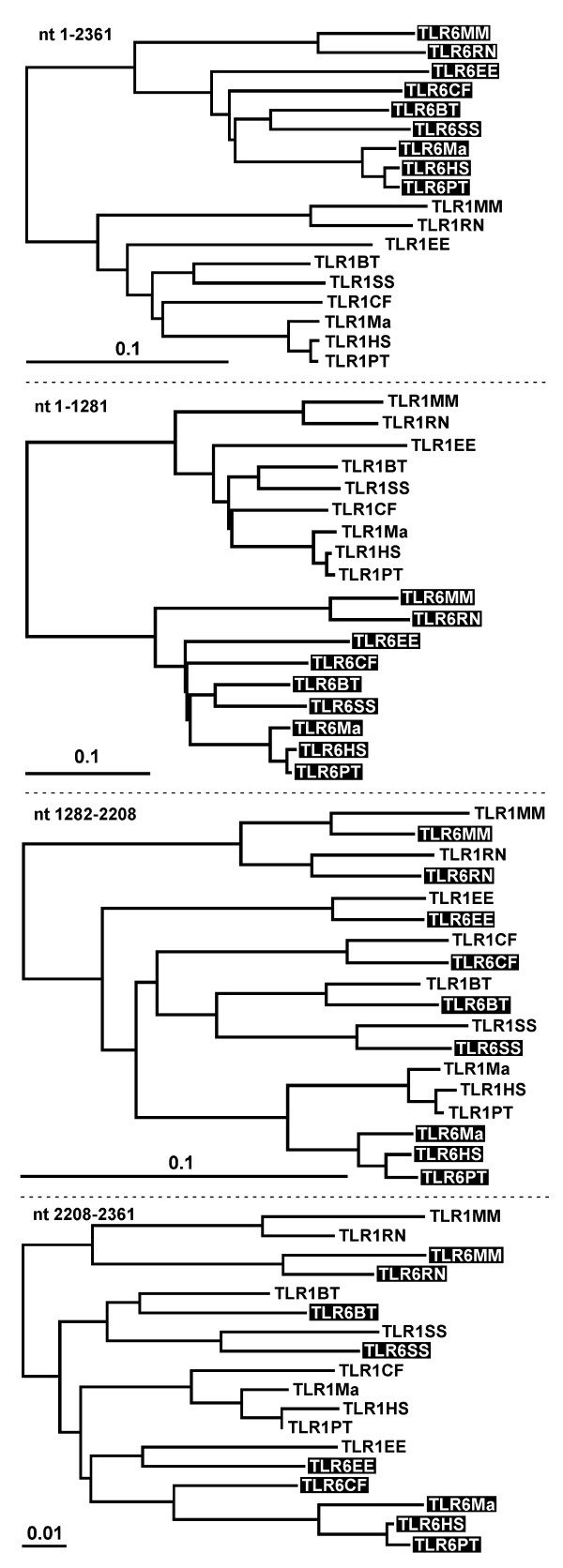
**Phylogenetic trees of mammalian TLR1 and TLR6**. Phylogenetic trees of DNA encoding the full length TLR1 and TLR6 proteins (nucleotide 1–2361), the region containing up to LRR16 (nucleotide 1–1281), the region containing LRR16 to the N-terminal three quarters of the TIR domain (nucleotide 1282–2208) and the C-terminal quarter of the TIR domain (nucleotide 2209–2361). Note that for the middle segment (nucleotide 1282 to 2208) the paralog TLR1 and TLR6 cluster together, whereas for the full length sequence and the 5' segments the orthologs cluster together.

To determine if the results for the region 437–745 could have been due to gene conversion, we created phylogenetic trees using DNA sequences encoding the full-length molecules and the different regions of sequence pattern described above (Figure 2); similar trees were observed when protein sequences were used (data not shown). Phylogenetic trees for DNA sequences encoding full length TLR1 or TLR6, as well as amino acid region 1–436 were concordant with a model in which TLR1 and TLR6 independently evolved from a common TLR1-like ancestor. The tree structure was compatible with the known evolutionary relationships among the mammalian species.

By contrast, for the DNA region encoding amino acids 437–745, the phylogenetic trees implied DNA transfer from TLR1 to TLR6 (or vice versa) by gene conversion independently occurring in the common ancestor of the primates and in the other six mammalian species (Figure 2). A closer analysis of the primate sequences gave some evidence for a gene conversion event occurring in this region after divergence of rhesus monkeys and the common ancestor of chimpanzees and humans, but branch lengths are too short to give reliable results. For the DNA region encoding amino acids 746-end, no clear phylogenetic relationships could be established. The rodent, dog and primate sequences of TLR1 and TLR6 clustered separately, whereas the cow, pig and hedgehog sequences of TLR1 and TLR6 clustered together (Figure 2). The phylogenetic tree for the first 300 bp of the 3' non-translated region was similar to that of the region encoding amino acid 1–436 (data not shown). Taken together, these data are compatible with independent gene conversion events starting in a region near the codon of amino acid 436 and ending, in all mammals, between the codon for amino acid 746 and the stop codon.

To further establish whether gene conversion has occurred, and identify the boundaries for each species, we made an analysis using the Geneconv program [12] (Table 1). As gene conversion between the TLR1 and TLR6 gene can only occur during meiotic division in the ancestor of a species, and not between species, we made a pairwise comparison of TLR1 and TLR6 sequences within each species. The results were highly significant (with p values ranging from 3.3 × 10^-37 ^to 3.5 × 10^-74^). The inferred boundaries for gene conversion started between nucleotide 1284 and 1306, which are within 25 bp downstream of the nucleotide position 1281 that was inferred by visual inspection of the protein sequences. For seven mammalian species the inferred 3' boundaries were situated between nucleotide 2204 and 2220, which is within 12 bp upstream of position 2208, inferred from the protein sequences. The inferred ends for the gene conversion fragments were 2287 for the cow TLR1/TLR6 sequences and 2313 for the pig sequences (Table 1).

**Table 1:** Gene conversion analysis for TLR1 and TLR6 sequences from nine mammalian species

	Fragments						
Sequence names	BC KA pvalue	Begin	End	Length	Num Poly	Num Dif	Total Difs

TLR1HS;TLR6HS	1.98 × 10^-41^	1289	2204	916	513	35	538
TLR1PT;TLR6PT	3.28 × 10^-37^	1289	2208	920	382	35	557
TLR1Ma;TLR6Ma	5.92 × 10^-43^	1289	2204	916	370	22	553
TLR1MM;TLR6MM	2.66 × 10^-69^	1284	2203	920	381	11	623
TLR1RN;TLR6RN	3.52 × 10^-74^	1306	2198	893	370	5	631
TLR1EE;TLR6EE	2.34 × 10^-58^	1281	2208	928	384	14	586
TLR1BT;TLR6BT	1.96 × 10^-44^	1305	2287	982	414	23	532
TLR1SS;TLR6SS	1.17 × 10^-43^	1281	2313	1033	434	26	524
TLR1CF;TLR6CF	1.49 × 10^-44^	1289	2208	920	382	16	535

BC KA p-values are Bonferroni-corrected Karlin-Altschul [34] p-values; the position of similar fragments is given with respect to the aligned human TLR1 DNA sequence [see Additional file 4]. "Num Poly" is the number of polymorphic sites in the fragment. "Num Dif" is the number of mismatches within the fragment. "Tot Difs" is the total number of mismatches between two sequences. HS: *Homo sapiens*; PT; *Pan Troglodytes*; Ma: *Macaca mulatta*; MM: *Mus musculus*; RN: *Rattus Norvegicus*; EE: *Erinaceus europaeus*; BT: *Bos taurus*; SS: *Sus scrofa*; CF: *Canis familiaris*.

To further discriminate whether the results described were due to gene conversion rather than convergent evolution we performed an analysis of codon usage for the conserved amino acids. Indeed, amino acid sequences that remained invariant through evolution or became identical through convergent evolution are less likely to be encoded by the same triplets than sequences that became identical after gene conversion. Overall the TLR1 and TLR6 paralogs share 66% amino acid identity [see Additional file 2]. Analysis of codon usage for conserved amino acids in TLR1 and TLR6 showed that on average identical codons were significantly (p < 0.0001) more frequently used to encode conserved amino acids in the region 437 to 744 (96.3 ± 1.7%) than in the regions encoding amino acids 1–436 (56.2 ± 3.5%) or 745 to end (64.7 ± 15.3%) (Table 2)

**Table 2:** Pattern of codon usage for conserved amino acids between TLR1 and TLR6

Species	Amino acid region 1–436	Amino acid region 437–744	Amino acid region 745 – end
*Homo sapiens*	57.2 (n = 215)	93.6 (n = 280)	57.1 (n = 35)
*Pan troglodytes*	56.3 (n = 213)	94.5 (n = 291)	61.8 (n = 34)
*Macaca mulatta*	58.5 (n = 207)	96.9 (n = 295)	62.5 (n = 32)
*Mus musculus*	52.1 (n = 188)	97.7 (n = 302)	43.8 (n = 32)
*Rattus norvegicus*	50.0 (n = 180)	97.7 (n = 304)	42.4 (n = 33)
*Erinaceus europaeus*	55.9 (n = 186)	97.7 (n = 304)	80.0 (n = 30)
*Bos Taurus*	58.0 (n = 212)	96.7 (n = 303)	80.6 (n = 36)
*Sus scrofa*	56.3 (n = 208)	94.4 (n = 302)	82.9 (n = 41)
*Canis familiaris*	61.8 (n = 191)	97.7 (n = 298)	71.4 (n = 35)
Average ± SD	56.2 ± 3.5 *	96.3 ± 1.7 *	64.7 ± 15.3 *

For three regions of TLR1 and TLR6 (amino acids 1 to 436, amino acids 437 to744 and amino acids 745 to end) we determined, for all conserved amino acids, whether the codon used in TLR1 or TLR6 was identical or synonymous. The results are expressed as identical/(identical + synonymous) × 100%. n : gives the number of conserved amino acids on which the averages were calculated.

* The percentage identical codon usage for conserved amino acids for the region 437–744 was significantly higher than for the regions 1–436 and 745-end.

TLR1, TLR6 and TLR10 share a common locus (chromosome 4p4 in humans). We investigated whether there was evidence for gene conversion between the genes for TLR10 and TLR1 or TLR6. Apart from a small region around nucleotides 2100 to 2200, in only the *Bos taurus *and *Sus scrofa *sequences, which is ancestral to the gene conversion events between TLR1 and TLR6 in these species, no evidence for gene conversion was observed [see Additional file 3].

TLR7 and TLR 8 share a common locus on the X-chromosome. We analyzed these genes, but observed no evidence for gene conversion (data not shown).

## Discussion

By comparing the ortholog and paralog protein sequences from a large number of species, functionally important sequences may be identified. Here, we made a comparative analysis of protein and coding DNA sequences of TLR1 and TLR6 from nine mammalian species, representing the mammalian orders primates, rodentia, insectivora, artiodactyla and carnivora. At the time of writing this article no complete sequence information for TLR1 or TLR6 was available for mammals representing other mammalian orders. We identified a 300 amino acid stretch near the C-terminal end of the proteins in which TLR1 and TLR6 from the same species were on average more than 96% identical, whereas orthologs of TLR1 and TLR6 were only 80% identical in this region (p < 0.0001). Our analyses show that the high degree of sequence identity between TLR1 and TLR6 from the same species is due to gene conversion that had independently occurred in six mammalian species and in an ancestor of three primates. Our data confirm and extend results of recent phylogenetic analyses showing that overall TIR domain sequences of TLR1 and TLR6 paralogs from three mammalian species clustered together, whereas the amino-terminal part of TLR1 or TLR6 or TIR domain sequences of other TLR genes clustered with their respective orthologs [10,11]. Our results show that the same applies not only for TIR domains of representatives of several other mammalian orders, but also that the boundaries for gene conversion are located at highly conserved positions. The region homogenized by gene conversion corresponds to the last four LRR motifs, the C-terminal cap, the transmembrane domain and three quarters of the TIR domain. We have no clear explanation as to why the boundaries of gene conversion are so highly conserved in most of the species. One explanation might be that the segments that were the subject of gene conversion lie directly adjacent to the segments that have TLR1- or TLR6-specific functions. Alternatively, the genes of both TLR1 and TLR6 might contain short conserved DNA segments that are particularly prone to gene conversion; co-evolution with common binding partners of TLR1 or TLR6 would then result in the preferential selection of the optimal intervening sequence.

Gene conversion is a common event in evolution and is a powerful means to restrain the divergence of paralog sequences. It appears to be favored when paralogs are located on the same chromosome at distances of less than 55 kb [13], as is the case for TLR1 and TLR6. Gene duplication and gene conversion in six mammalian species have shaped the paired immunoglobulin-like receptor (*PILR*) locus, which encodes regulators of the innate and adaptive immune systems with PILRA being an inhibitory receptor, and PILRB, its activating counterpart [14]. In a striking example of concerted evolution, EMR2, a member of the epidermal growth factor TM7 family, possesses a chimeric structure with a seven-span transmembrane region most related to EMR3 and an EGF domain region nearly identical to CD97. The chimeric structure of EMR2 has been continuously maintained since early mammalian radiation by gene conversion events between different regions of the EMR2 gene and the oppositely orientated and physically adjacent genes *CD97 *and *EMR3 *[15].

We may postulate that the unusual conservation of sequence identity for the region 436 to 746 of TLR1 and TLR6, in all nine mammalian species for which complete sequence information is available, reflects strong selective mechanisms that restrained divergence of this region. However, in view of the conserved gene conversion boundaries, restriction of high sequence identity to the region 436–746 allowed (or favored) independent evolution of the N-terminal region and extreme C-terminal region of TLR1 and TLR6. Information on the function of the various domains of TLR1 and TLR6 has become available in recent years and may support this postulate. Heterodimers of TLR1 or TLR6 with TLR2 mediate cell activation in response to a large number of diacyl and triacyl ligands. Gene duplication of the ancestor of TLR1 and TLR6 enabled divergence between the two receptors leading to an increase in the repertoire of lipopeptides that can be recognized by TLR2 heterodimers. Transfection of human embryonic kidney cells for expression of TLR1 or TLR6, in combination with TLR2 and CD14, made cells responsive to tri- and di-acylated lipopeptides, respectively [8]. Examination of chimeric receptors, generated by domain exchange between TLR1 and TLR6 revealed that LRR9-12 enables these receptors to discriminate between tri- and di-acylated lipopeptides [9]. The greater sequence identity of the TLR1 and TLR6 orthologs, and lower sequence identity of the paralogs in these domains, is in accordance with a preservation of lipopeptide discrimination potential during mammalian evolution.

The higher sequence identity of TLR1/6 paralogs for the amino acid region 436 to 746, generated by gene conversion, might reflect the need for TLR1 and TLR6 to co-evolve with common binding partners, in such a way that functional interaction surfaces are maintained. Analysis by fluorescence resonance energy transfer has shown that TLR1/TLR2 and TLR6/TLR2 heterodimers are present at the cell surface even in the absence of lipopeptide ligands [8]. It is therefore likely that TLR1 or TLR6 have at least to co-evolve with TLR2 to maintain a functional extracellular dimer interaction site. The localization of the interaction site may be inferred from the crystal structure of TLR3, which revealed homodimer formation with the dimer interface located in the C-terminal LRR motifs [16], a region corresponding to the N-terminal end of the conserved TLR1/TLR6 paralog region we describe. To what extent these dimer interfaces of TLR3 may be relevant for TLR2-mediated signaling remains to be established, because the formation of the TLR3 dimer structure might be a transient or forced encounter and therefore of questionable biological significance (for a discussion see [17]).

The contrasting requirement for diversity of ligand binding sites located in LRR9-12 that need to respond to evolutionary pressure from pathogens and co-evolution of dimer interaction sites in the C-terminal LRR motifs, is in agreement with the location in LRR16 of the gene conversion start sites. Our finding that in all species gene conversion starts in an almost identical position (Table 1) suggests that the LRR13-15 region is also important for ligand recognition and discrimination. In this context it is interesting to note that the dsRNA binding site in TLR3 is located at amino acids H539 and N541 [18]. In an alignment with TLR1 and TLR6, these amino acids are located immediately prior to the common start position for gene conversion between TLR1 and TLR6.

Other putative extracellular binding partners with which TLR1 and TLR6 might need to co-evolve are CD14 [8] and CD36 [19]. These proteins are associated with the TLR1/TLR2 and TLR6/TLR2 heterodimers when TLR ligands are present [8]. CD14 and CD36 are able to bind triacylated and diacylated lipopeptides and appear to serve as "sensors" or scavenger receptors to present the lipopeptides to TLR2 heterodimers [19,20]. However, direct binding of CD14 to TLR1 or TLR2 could not be detected [20].

Known or putative binding partners for the intracellular TIR domains of TLR1 and TLR6 are the TIR domains of TLR2, MyD88 and TIRAP [21,22]. The need to co-evolve with these functional binding partners may explain the extension of the preserved gene conversion sequence to the intracellular region. Recently, specific information has become available on the interactions between the TIR domains of TLR1 or TLR6 and intracellular binding partners. Site-directed mutagenesis and computer-based docking studies of the TIR domains of TLR1 and TLR2 revealed an interaction between Gly-676 (numbering according to reference [23] and corresponding to position 685 [see Additional file 1]) of the TLR1 BB loop with residues Arg-748 and Phe-749 of the TLR2 DD loop [23]. At this position TLR1 and TLR6 are identical in all species. It is interesting to note that at two other interaction sites orthologs are almost identical to each other and different from their paralogs. One of these interaction sites, which lies within the region homogenized by gene conversion, corresponds to His-646 in TLR1 [position 655 in Additional file 1]. The backbone NH of His-646 of TLR1 is predicted to form an intramolecular H-bond with the side chain of Asn-700, maintaining their tertiary structure in the TLR1/2 complex [23]. In addition, Asn-700 interacts with Asp-730 in TLR2 and an electrostatic interaction is predicted for side chains of TLR1 His-646 and TLR2 Asp-730. Next to His-646, at residue 645 [position 654 in Additional file 1], in eight out of nine species analyzed, TLR1 has a glycine, whereas all TLR6 sequences have glutamic acid. This suggests that selective mechanisms have maintained (or re-established) these distinct amino acids within the region of gene conversion. A third TLR1-TLR2 interaction site contains Tyr-737 of TLR1 [position 746 in Additional file 1], which for most mammals lies exactly at the 3' end of the region homogenized by gene conversion. Here all 9 TLR1 sequences analyzed have a tyrosine, while all TLR6 sequences have an asparagine. The observation that, for two out of three interaction sites, orthologous amino acids are identical and different from their paralogs, implies the existence of subtle structural differences between the TIR domain heterodimers of TLR1 and TLR6 with TLR2 that were maintained despite gene conversion events in their vicinity. To what extent this translates into functional differences between TLR1 and TLR6 remains to be established.

**Figure 3:**
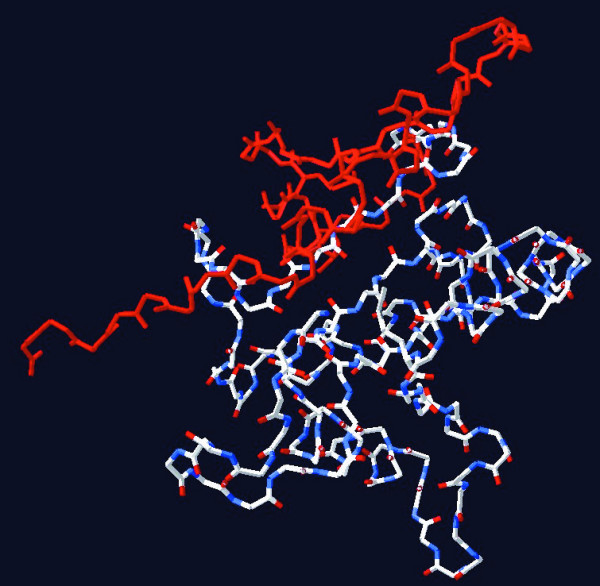
**Three-dimensional model of the TIR domain of human TLR1**. In red: amino acids 746 to 795 [numbering according to Additional file 1]. Note that the amino acids all lie in one plane at the surface of the TIR domain.

A common binding partner of TLR-TIR domains is MyD88. Recent results suggest that the TLR2-TIR domain can bind MyD88, whereas the TLR1-TIR and TLR6-TIR domains do not [24]. However, mutation of one TLR1 amino acid from Asn-672 to Asp [position 681 in Additional file 1] enables TLR1 to bind MyD88, suggesting that these two proteins have almost complementary surfaces. It remains to be established whether, in the context of lipopeptide-activated cells, MyD88 has the ability to bind TLR1. A second important molecule for signaling through TLR1/TLR2, TLR6/TLR2 or TLR4 is TIRAP, a sorting adapter molecule which facilitates MyD88 recruitment to the plasma membrane [25]. Presently, no information is available on direct binding of TIRAP to TLR1 or TLR6. Additional intracellular TLR binding proteins are heat shock protein 60 and the RNA helicase DDX36 (DHX36), which bind the TIR domain of TLR1, and heat shock protein 75, which binds TLR1 and TLR6 [24]. The interaction sites between these proteins and TLR1 or TLR6 are not yet determined. Taken together, the large number of common binding partners places severe co-evolutionary constraints on the TIR domains of TLR1 and TLR6, which may explain the occurrence of gene conversion events in this region. However, these constraints do not explain why the 3' ends of the gene conversion region lies within 15 bp of each other for 7 out of 9 analyzed mammalian species, and why for the region downstream of this site the TLR1 or TLR6 orthologs are again more similar to each other than to their respective paralogs. From the crystal structure of the TIR domain of TLR1 [26] we may conclude that the corresponding amino acids (745-end) all lie in one plane at the surface of the TIR domain (Figure 3). An intriguing possibility would be that TLR1 and TLR6 each interact with a different intracellular binding partner – either one of those mentioned above or originated by duplication of an ancestral binding partner of the TLR1/TLR6 ancestor – which might mediate intracellular responses that are specific for TLR1 or TLR6. However, the existence of such hypothetical intracellular TLR1- or TLR6-specific binding partners remains to be established.

The C-terminal cap motif and the transmembrane domain of TLR1 and TLR6 also show higher paralog than ortholog sequence identity. This either implies a requirement for co-evolution with as yet unknown binding partners for these regions, or that the selective mechanisms operating on LRR16-19 and the TIR domain stimulate gene conversion events that drag the intervening C-terminal cap and transmembrane sequences along.

TLR10 shares a common locus with TLR1 and TLR6. It is an orphan receptor that can form homodimers and heterodimers with TLR1 and TLR2, has a TIR domain that is functionally active and uses MyD88 as an adapter molecule [27]. Studies on the in vivo role of TLR10 are hampered by the fact that in mice TLR10 is a nonfunctional pseudogene. We investigated whether gene conversion had occurred between TLR10 and TLR1 or TLR6, but found no evidence for gene conversion except for a very small region in the genomes of *Bos Taurus *and *Sus scrofa*, which is ancestral to the gene conversion events between TLR1 and TLR6 in these species. The absence of consistent gene conversion between TLR10 and TLR1 or TLR6 is most likely due to the more recent divergence of TLR1 and TLR6 from a common ancestor, as reflected by the phylogenetic trees and the higher sequence identity between TLR1 and TLR6 (around 80%) as compared to that between TLR10 and TLR1 or TLR6 (less than 50%). Furthermore, the common binding partners of TLR1 and TLR6, which drive the coevolution of these proteins, may not be the same as the binding partners of TLR10.

## Conclusion

The results reported here show that the paralogous mammalian TLR1 and TLR6 genes have a conserved region of extremely high sequence identity that has been maintained by gene conversion. This implies that gene conversion was a common and useful evolutionary mechanism for these TLRs. Our findings show that protein paralogs with multiple functional domains can maintain common characteristics for specific domains by gene conversion, while allowing other domains to evolve diversified functions. Given the large number of paralogs that are present in a mammalian genome, and the common occurrence of multiprotein complexes, it is to be expected that similar examples of gene conversion will be discovered. By recognizing sequences maintained by gene conversion, through careful phylogenetic analysis of these genomes in several mammalian species, it may be possible to identify novel protein-protein interaction sites and functionally important protein sub-structures.

## Methods

The analysis is based on gene sequences coding for TLR1 and TLR6 from nine different mammalian species: *Homo sapiens*, *Pan troglodytes*, *Macaca mulatta *(representing the order Primates); *Mus musculus *and *Rattus norvegicus *(representing the order Rodentia); *Erinaceus europaeus *(representing the order Insectivora), *Bos taurus *and *Sus scrofa *(representing two families of the order Artiodactyla) and *Canis familiaris *(representing the order Carnivora). TLR10 sequences were from the same species except *Mus Musculus *that has a TLR10 pseudogene and *Erinaceus europaeus *for which no TLR10 sequence is available. All sequences were obtained from the Ensembl genome browser (release 44)[28], except the *Sus scrofa *sequences which were obtained as cDNA sequences from the NCBI nucleotide server [29]. For all TLR1, TLR6 and TLR10 genes the entire open reading frame is coded on a single exon. Also, we found no evidence for retroelements in the TLR1 and TLR6 genes. The numbering of the LRRs in TLR1 and TLR6 is according to Bell et al. [4] and is specified [see Additional file 1]. The DNA sequences used for the analyses are given [see Additional file 4].

### Note added in proof.        

The crystal structure of the TLR1-TLR2 heterodimer bound to a tri-acylated lipopeptide has been determined [35]. The interface between TLR1 and TLR2 was found to be located between LRR11 and LRR14.

Multiple alignment of protein and DNA sequences and calculations of percentage identities were made using ClustalW software [30] and phylogenetic trees of full length molecules, or segments thereof, created by the Difference of Sums of Squares method using Topali software [31] with default parameters and a bootstrap value of 1000. Codon usage for conserved amino acids was analyzed manually. Statistical analyses for differences in sequence identity of full length TLRs, or segments thereof, as well as of codon usage for conserved amino acids was analyzed by the non-parametric Wilcoxon two sample test. Detection of most likely borders of gene conversion events and statistics were done using Geneconv software [12]. The crystal structure of the TIR domain of TLR1 was determined by Xu et al. [26] and obtained from the protein databank with access number 1fyv [32]. The structure was visualized using the Swiss-PdbViewer program [33].

## Abbreviations

TLR, Toll-like receptor; LRR, Leucine-rich repeat; PAMP, Pathogen-associated molecular pattern; LPS, Lipopolysaccharide; TIR, Toll/interleukin-1 receptor.

## Authors' contributions

EK developed the idea, designed the study, performed the analysis and wrote the paper. NP, JL, SDG and RF contributed to the analysis of the data and the writing of the paper. All authors read and approved the final manuscript.

## Supplementary Material

Additional file 1 **Alignment of TLR1 and TLR6 amino acid sequences from nine mammalian species**. Sequences from *Homo sapiens *(HS), *Pan troglodytes *(PT), *Macaca mulatta *(Ma), *Mus musculus *(MM), *Rattus norvegicus *(RN), *Erinaceus europaeus *(EE), *Bos taurus *(BT), *Sus scrofa *(SS) and *Canis familiaris *(CF) are detailed. Three patterns of similarity are observed at a particular position: A) ortholog sequences are more identical; b) paralog sequences are more identical; C) a high over all identity between TLR1 and TLR6. Color codes: Pattern A) in light blue (or pink): the same amino acid is conserved in 7 or more of 9 ortholog TLR1 (or TLR6) sequences and present in two or less TLR6 (or TLR1) sequences. Pattern B) in red, green, dark blue etc.: the sequences of the TLR1 and TLR6 paralogs are identical in at least seven species. Pattern C) in yellow: the same amino acid is conserved in 14 or more out of 18 TLR1 and TLR6 sequences and patterns A or B do not apply. NB: the alignments do not show the N-terminal sequence MVKSLWDSLCN, which is found only in mouse and rat TLR6. Click here for file

Additional file 2 **Sequence similarity of partial and full length sequences of TLR1 and TLR6 from six mammalian species**. Sequences of TLR1 and TLR6 from six mammalian species representing five mammalian orders were analysed: *Homo sapiens *(HS1 and HS6), *Pan troglodytes *(PT1 and PT6), *Macaca mulatta *(Ma1 and Ma6), *Mus musculus *(MM1 and MM6), *Rattus norvegicus *(RN1 and RN6), *Erinaceus europaeus *(EE1 and EE6), *Bos taurus *(BT1 and BT6), *Sus scrofa *(SS1 and SS6) and *Canis familiaris *(CF1 and CF6). Pairwise percentage identities were calculated for all full length protein sequences and for the regions N-termini to amino acid 436, amino acids 437 to 744 and amino acid 745 to C-termini. Average results for percentage identities between orthologs and paralogs, as well as statistics, are given in section A). Individual results for comparisons between TLR1 and TLR6 orthologs (red and blue, respectively; n = 15), and the TLR1/TLR6 paralogs (green; n = 6), are given in B). Click here for file

Additional file 3 **Phylogenetic trees of mammalian TLR1, TLR6 and TLR10**. Top: phylogenetic tree of DNA encoding the region containing LRR16 to the N-terminal three quarters of the TIR domain (nucleotide 1282–2208). Note that for this segment the paralog TLR1 and TLR6 sequences cluster together for all non-primate sequences and are separate from the clustered TLR10 sequences. Bottom: For the small nucleotide region 2100 – 2200 the paralog TLR1, TLR6 and TLR 10 sequences cluster together, but only for bovine and swine sequences. Click here for file

Additional file 4 **DNA and protein sequences for TLR1 and TLR6 used for analyses**. TLR1 and TLR6 sequences were analysed from nine mammalian species: *Homo sapiens *(HS), *Pan troglodytes *(PT), *Macaca mulatta *(Ma) (representing the order Primates); *Mus musculus *(MM) and *Rattus norvegicus *(RN) (representing the order Rodentia); *Erinaceus europaeus *(EE) (representing the order Insectivora), *Bos taurus *(BT) and *Sus scrofa *(SS) (representing two families of the order Artiodactyla) and *Canis familiaris *(CF) (representing the order Carnivora). All sequences were obtained from the Ensembl genome browser [28], except the *Sus scrofa *sequences which were obtained from the NCBI nucleotide server [29]. The open reading frame start and stop codons are underlined in the DNA sequences. A DNA sequence alignment is given, with identical nucleotides marked with an * at the bottom of the alignment. In the protein sequences blue-yellow and yellow-purple highlights indicate the boundaries between the regions 1 to 436, 437 to 745 and 746 to end. Protein alignments are given in additional file 1. Click here for file
