# Supplementary material for: Gene conversion limits divergence of mammalian TLR1 and TLR6
Source: BMC Evol Biol. 2007 Aug 29;7:148. doi: 10.1186/1471-2148-7-148 (PMC2077338; doi:10.1186/1471-2148-7-148)
Supplement: Additional file 1 — Alignment of TLR1 and TLR6 amino acid sequences from nine mammalian species. Sequences from Homo sapiens (HS), Pan troglodytes (PT), Macaca mulatta (Ma), Mus musculus (MM), Rattus norvegicus (RN), Erinaceus europaeus (EE), Bos taurus (BT), Sus scrofa (SS) and Canis familiaris (CF) are detailed. Three patterns of similarity are observed at a particular position: A) ortholog sequences are more identical; b) paralog sequences are more identical; C) a high over all identity between TLR1 and TLR6. Color codes: Pattern A) in light blue (or pink): the same amino acid is conserved in 7 or more of 9 ortholog TLR1 (or TLR6) sequences and present in two or less TLR6 (or TLR1) sequences. Pattern B) in red, green, dark blue etc.: the sequences of the TLR1 and TLR6 paralogs are identical in at least seven species. Pattern C) in yellow: the same amino acid is conserved in 14 or more out of 18 TLR1 and TLR6 sequences and patterns A or B do not apply. NB: the alignments do not show the N-terminal sequence MVKSLWDSLCN, which is found only in mouse and rat TLR6. [file 1471-2148-7-148-S1.doc]

**Additional file 1**

# Alignment of TLR1 and TLR6 sequences

Sequences from *Homo sapiens* (HS), *Pan troglodytes* (PT), *Macaca mulatta* (Ma), *Mus musculus* (MM), *Rattus norvegicus* (RN), *Erinaceus europaeus* (EE), *Bos taurus* (BT), *Sus scrofa* (SS) and *Canis familiaris* (CF) are detailed. Three patterns of similarity are observed at a particular position: A) ortholog sequences are more identical; B) paralog sequences are more identical; C) a high over all identity between TLR1 and TLR6. Color codes: Pattern A) in light blue (or pink): the same amino acid is conserved in 7 or more of 9 ortholog TLR1 (or TLR6) sequences and present in two or less TLR6 (or TLR1) sequences. Pattern B) in red, green, dark blue etc.: the sequences of the TLR1 and TLR6 paralogs are identical in at least seven species. Pattern C) in yellow: the same amino acid is conserved in 14 or more out of 18 TLR1 and TLR6 sequences and patterns A or B do not apply. NB: the alignments do not show the N-terminal sequence MVKSLWDSLCN, which is found only in mouse and rat TLR6.

10 20 30 40 50 60

| | | | | |

TLR1HS -------MTSIFHFAIIFMLILQIRIQLSEESEFLVDRSKNGLIHVPKDLSQKTTILNIS

TLR1PT -------MPSIFHFAIIFMLILQIRIQLSEESEFLVDRSKNGLIHVPKDLSQKTTILNIS

TLR1Ma -------MTSIFHFAIIFMLTLQIRIQLSEESEFLVDRSKNSLIHVPKDLSQKTTILNIS

TLR1MM ---MTKPNSLIFYCIIVLGLTL-MKIQLSEECELIIKRPNANLTRVPKDLPLQTTTLDLS

TLR1RN ---MTKTQSTIFYCIVVLGLIL-IKIQLSEESELIIKRPNANLTRVPKDLPLQTTTLDVS

TLR1EE ---MTKTYSIVFHLIIIFMLIVKIRTLLSDGSDVLADRSNRTLIHIPKDLPPSTTILNVS

TLR1BT ---MTKKNSSIFHFAIIFILILEIRTQLSDESEFLIDRSKRGLTYVPKNLSLETTILDIS

TLR1SS ---MTKENLSIFHFAIIFILILEIRIQLSEESEVLVDRSKTGLTHVPKDLSLETTILDLS

TLR1CF ---MMKTNPSIFQFAIIFILILEIRIQLSEESDFLVNRSKAGLFHIPKDLSLKTTILDIS

TLR6HS MTKDKEPIVKSFHFVCLMIIIVGTRIQFSDGNEFAVDKSKRGLIHVPKDLPLKTKVLDMS

TLR6PT MTKDKEPIVKSFHFVCLMIIIVGTRIHFSDGNEFAVDKSKRGLIHVPKDLPLKTKVLDMS

TLR6Ma MTKDKEPVVKSFHFVCLMIIIVGTRIQFSDGSEFAVDKSKRGLTHVPKDLPPKTKVLDMS

TLR6MM MSQDRKPIVGSFHFVCALALIVGSMTPFSNELESMVDYSNRNLTHVPKDLPPRTKALSLS

TLR6RN MSQDREPIVESFHFVCTLALIVGSMTQFSDELESVVDYSNKNLTHVPKDLSPSTKSLSLS

TLR6EE MTRDKESTMRSFAYT--VIVTVGIVIQSSDENEFTVDLSKRGLTHIPRDLPSQTEVLDMS

TLR6BT MIKDKESPIRSCHFVYIVALVFGTIIQFSDESEFVVDMSKTSLIHVPKDLPPKTKVLDLS

TLR6SS MSKDKEPTVISLHSVYVMTLVWGTLIQFSEESEFVVDKSKIGLTRVPKDLPPQTKVLDVS

TLR6CF MIKDKDSITGSFHFVYIVTLIVGTIIQFSDESEFTVDMSNMNLTHVPEDLPPKTKILDMS

|--------N-cap--------||­­­­­­­-------

70 80 90 100 110 120

| | | | | |

TLR1HS QNYISELWTSDILSLSKLRILIISHNRIQYLDISVFKFNQELEYLDLSHNKLVKISCHPT

TLR1PT QNYISELWTSDILSLSKLRILIISHNRIQYLDISVFKFNHELEYLDLSHNKLVKISCHPT

TLR1Ma QNYISELWTSDILSLSKLRILIISHNRLQYLDISVFKFNQELEYLDLSHNKLAKISCHPT

TLR1MM QNNISELQTSDILSLSKLRVLIMSYNRLQYLNISVFKFNTELEYLDLSHNELKVILCHPT

TLR1RN QNNISELQTSDILLLSKLRVFIMSYNRLQYLNISVFKFNTELEYLDLSHNELRLISCHAT

TLR1EE HNYISELWASDILSLSKLKILIMSHNRIQNLDISVFRFNQELEYLDLSHNKLETISCHST

TLR1BT YNYISELQMPDILSLSKLKILIISHNRIQYLDLSVFKFNQELEYLDLSHNNLEKISCHPT

TLR1SS QNSISELQTSDILSLSKLRVFIISHNRIQYLDVSVFKFNQELEYLDLSHNKLEKISCHPM

TLR1CF QNYISELQTSDILSLSKLRILIVSYNRIQYLDISVFKFNQELEYLDLSHNELGRISCHPT

TLR6HS QNYIAELQVSDMSFLSELTVLRLSHNRIQLLDLSVFKFNQDLEYLDLSHNQLQKISCHPI

TLR6PT QNYIAELQVSDMSFLSELKVLRLSHNRIQLLDLSVFKFNQE-EYLDLSHNQLQKISCHPI

TLR6Ma HNYIAELQVSDISFLSELKVLRLSHNKIQLLDLSVFKFNQDLEYLDLSHNQLQKISCHPI

TLR6MM QNSISELRMPDISFLSELRVLRLSHNRIRSLDFHVFLFNQDLEYLDVSHNRLQNISCCPM

TLR6RN QNSISDLQMSDISFLSELRVLRLSHNRIRRLDFGVFLLNRDLEYLDVSHNQLQNISCCPM

TLR6EE QNNVSELHLSDMSLISRLKVLRLSHNRIQCLDFSVFRFNQDLEYLDLSHNQLKKLSCHAI

TLR6BT QNNISELHLSDISFLSGLRVLRLSHNRIQGLDISIFKFNHDLEYLDLSHNQLQKISCHPI

TLR6SS QNFITELHLSDISFLSQLTVLRLSQNRMQCLDISVFKFNQDLEYLDLSHNQLQTILCHPI

TLR6CF QNNISELHLSDMSYLSGLKILRISHNRIWWLDFSIFKFNQDLEYLDLSYNQLRNMSCHLI

---LRR1--------||---------LRR2---------||----- LRR3 --------

130 140 150 160 170 180

| | | | | |

TLR1HS VN-LKHLDLSFNAFDALPICKEFGNMSQLKFLGLSTTHLEKSSVLPIAHLNISKVLLVLG

TLR1PT VN-LKHLDLSFNAFDALPICKEFGNMSQLKFLGLSTTHLEKSSVLPIAHLNISKILLVLG

TLR1Ma VN-LKHLDLSFNAFDALPICKEFGNMSQLKFLGLSTTHLEKSTVLPIAHLNISKVLLVLG

TLR1MM VS-LKHLDLSFNAFDALPICKEFGNMSQLQFLGLSGSRVQSSSVQLIAHLNISKVLLVLG

TLR1RN AD-LKHLDLSFNAFDALPICKEFGNLSQLQFLGLSGSQIQNSSVQLIAHLNISKVLLVLG

TLR1EE AN-LKHLDLSFNAFVFLPICKEFGNMSQLEFLGLSASQLQKSRLLSISHLHISKVLLVLG

TLR1BT LN-LKHLDLSFNPFDALPICQEFGNMSQLEFLGLSATQLQKSSVQSITHLHISKVLLVLG

TLR1SS LN-LKHLDLSFNAFDALPICQEFGSMFQLEFLGLSATQLQKSSVLPIAHLHIGKVLLVLG

TLR1CF VN-LKHLDLSFNAFDDLPICKEFGNMSQLEFLGLSATQLQKSSMLPIASLHIRKVLLVLG

TLR6HS VS-FRHLDLSFNDFKALPICKEFGNLSQLNFLGLSAMKLQKLDLLPIAHLHLSYILLDLR

TLR6PT VS-FRHLDLSFNDFKALPICKEFGNLSQLNFLGLSAMKLQKLDLLPIAHLHLSYILLDLR

TLR6Ma MS-FRHLDLSFNDFEALPICKQFGNLSQLNFLGLSAMKLQKLDLLPIAHLHLSYILLDLR

TLR6MM AS-LRHLDLSFNDFDVLPVCKEFGNLTKLTFLGLSAAKFRQLDLLPVAHLHLSCILLDLV

TLR6RN VN-LKHLDLSFNDFEVLPVYKEFGNLRKLSFLGLSAAKFRQLDLLPISHLHLSCVLLDLV

TLR6EE QS-LQHLDLSFNDFDSLPICKEFGNLTQLNFLGLSTSKLQQLDLLPVSHLHVDSILLDLQ

TLR6BT TTTLKHLDLSFNDFDALPICKEFGNLTQLNFLGLSATKLQQLDLLPIAHLHLSCILLDLE

TLR6SS TS-LKHLDLSFNDFEALPICKEFGNLTQLNFLGLSATKLQQLDLLPIAHLHLSCILLDLE

TLR6CF RS-LKHLDLSFNDFHVLPICKEFGNLTQLQFLGLSATKLRQLDLLPIAHLHLSYILLDLQ

||------LRR4-----------||----------LRR5----------||---------

190 200 210 220 230 240

| | | | | |

TLR1HS ETYGEKEDPEGLQDFNTESLHIVFPTNKEFHFILDVSVKTVANLELSNIKCVLEDNKCSY

TLR1PT ETYGEKEDPEGLQDFNTESLHIVFPTNKEFHFILDVSVKTVANLELSNIKCVLEDNKCSY

TLR1Ma EHYGDKEDPEGLQNFNTESLHIVFPTSKEFNFILDVSVRTVANLELSNIKCVLEDNECSY

TLR1MM DAYGEKEDPESLRHVSTETLHIVFPSKREFRFLLDVSVSTTIGLELSNIKCVLEDQGCSY

TLR1RN DTYGEKEDPKCLQHISTETLHIVFPSKREFHFLLDMSVSTAISLELSNIKCVLEDKNCSY

TLR1EE DSYGEKEIPDSLQDLNTESLHIVFPLGKEFHFNLDVSISQAVSLELSNIQYVLEDGGACS

TLR1BT DTYGEREDAESLQDLKTQSLHIVFPTGKEFHFILDVSVGTTVSLELSNIKCVLDDNGCPY

TLR1SS DSYGEREDPESLQDLNTQSLHIVYPPGKEFHFMLDVSVSTAVNLELSNIRCVLDANGCHH

TLR1CF DTYGKKEDPESLQKLNTESLHIVFPIRKEFSFTLDVSVSTAVSLELSNIKCVPDGHGWSY

TLR6HS NYYIKENETESLQILNAKTLHLVFHPTSLFAIQVNISVNTLGCLQLTNIK—-LNDDNCQV

TLR6PT NYYIKENETESLQILNAKTLHLVFHPTSLFAIQVNISVNTLGCLQLTNIK--LNDDNCQV

TLR6Ma NYYIKENETESLQILNAKTLHLVFHPTSLFSIQVNISVNTLGCLQLTNIK--LNDDNCQV

TLR6MM SYHIKGGETESLQIPNTTVLHLVFHPNSLFSVQVNMSVNALGHLQLSNIK--LNDENCQR

TLR6RN NYQIKDGETESLQVPNTNVLHLVFHPNSLFSVQVNISVNALGCLQLSNIK--LNDENCQS

TLR6EE GYSMKGNETGSLQIFNTKKLHFVFHPNDLFSVLVNISVNMIECLQLTNIK--LNDDNCQV

TLR6BT D-YMKENKKESLQILNTKKLHLVFHPNSFFSVQVDISGNSLACLQLTNIK--LNDYNCQV

TLR6SS RYYMKENEKESLQILNTKKLHLVFHPNSFFSVQVNISVKSVGCLQLANIK--LSDDNCQV

TLR6CF GYYAKESEKGSLQILDTKTLHLVFHPNQLFSVQANMLVNNLGCLQLTNIK--LNNDNCQV

--LRR6----------||­­­­­-----------LRR7------------||-------------

250 260 270 280 290 300

| | | | | |

TLR1HS FLSILAK-LQTNPKLSNLTLNNIETTWNSFIRILQLVWHTTVWYFSISNVKLQGQLDFRD

TLR1PT FLSILAK-LQTNPKLSSLTLNNIETTWNSFIRILQLVWHTTVWYFSISNVKLQGQLDFRD

TLR1Ma FLNILAK-LQTNPKLSSLTLNNIETTWNSFIRILQLVWHTTVWYFSISNVKLQGQLDFRD

TLR1MM FLRALSK-LGKNLKLSNLTLNNVETTWNSFINILQIVWHTPVKYFSISNVKLQGQLAFRM

TLR1RN FLGTLER-LRKTQRLSNLTLNNVDTTWNSFINILQLVWHTPVKSFSISNVKLKGHFNFRR

TLR1EE FQNALRK-LQKNPRLSNLTLNNIDTTWNSFMMILQLVWHTGVEYFSIKNVKLQGWFHPRE

TLR1BT FENVLSK-LQKNSRLSNLTLNNIEITWNSFFTILQLVWRTNIEYFSISNVKLQGYLDSRD

TLR1SS FQNVLLK-LQKNSKLSNLTLNNIETTWNSFITTLQFVWHTSIEYFSISSVKLQGQLDFRD

TLR1CF FQNVLSK-LQKNSRLSSLTLNNIETTWNFFIMLLQLVWHTSIEYFSISNVKLQGYPDFRD

TLR6HS FIKFLSE-LTRGSTLLNFTLNHIETTWKCLVRVFQFLWPKPVEYLNIYNLTIIESIREED

TLR6PT FIKFLSE-LTRGPTLLNFTLNHIETTWKCLVRVFQFLWPKPVEYLNIYNLTIIESIHEEE

TLR6Ma FIKFLLE-LTRGPTLLNFTLNHIETTWKCLVRVFQFLWPKPVEYLNIYNLTIIESIHEED

TLR6MM LMTFLSE-LTRGPTLLNVTLQHIETTWKCSVKLFQFFWPRPVEYLNIYNLTITERIDREE

TLR6RN LIIFLSE-LTRGPTLLNLTLQHIETNWKCFVRLLQFLWPRPVEYLNIYNLTITESISRET

TLR6EE LINFLSEPLIRGPNLLNLTLDHVETTWKCLVRVFQSLWSKPIEYLNIYNLTVVERIDEEE

TLR6BT LLKFLSG-LTGGPTLLNFTLNHVETTWKCLVKVFQFLWPKPIEYLNIYNLTIVESIDEEV

TLR6SS FITFLLE-LTQGPTLLNFTLNHVETTWKCLVGIFQFLWPKPVEYLSIYNLTIVESIDEED

TLR6CF LIQFLSE-LTRGPTLLNFTLQHVKTTWKCLVRIFKFLWPKPVQYLNIYNLTIVESINKEY

LRR8------||--------------LRR9--------------||--------LRR10-

310 320 330 340 350 360

| | | | | |

TLR1HS FDYSGTSLKALSIHQVVSDVFGFPQSYIYEIFSNMNIKNFTVSGTRMVHMLCPSKISPFL

TLR1PT FDYSGTSLKALSIHQVVSDVFSFPQSDIYEIFSNMNIKNFTVSGTRMVHMLCPSKISPFL

TLR1Ma FDYSGTSLKALSVHQVVSDVFNFPQRDIYEIFSNMNIKNFTVSGTRMIHMVCPSKISPFL

TLR1MM FNYSDTSLKALSIHQVVTDVFSFPQSYIYSIFANMNIQNFTMSGTHMVHMLCPSQVSPFL

TLR1RN FHYSDTSLRALSIHQVVTDVFSFPQSNIYSIFSNMNIQSFTVSGTRMVHMLCPDQISPFL

TLR1EE FNYSDTSLKSLTIHQVVNNAYSLEQNSIYKIFANMNIQHFTVSGTPMVHMLCPLQTSPFL

TLR1BT FDYSDTSLKALSIHKVVHDVFSLPQGYVYKILSNMNIQHLTVSAAHMVHMVCPSQISPFL

TLR1SS FDYSDTSLKALSLHQVVSEVFSFPQSYIYKIFSNMNIQYLTVSATHMVHMVCPSQISPFL

TLR1CF FDYSDTSLKALSIHQVVSNAFNLPQSYIYKIFSNMNIQNFTVSGTHMVHMVCPSQISPFL

TLR6HS FTYSKTTLKALTIEHITNQVFLFSQTALYTVFSEMNIMMLTISDTPFIHMLCPHAPSTFK

TLR6PT FTYSKTTLKALKIEHITNKVFLFSQTALYTVFSEMNIMMLTISDTPFIHMLCPHAPSTFK

TLR6Ma FTYSKTTLKALKIEHITNQVFIFSQTALYTVFSEMNIMMLTISDTPFIHMLCPRAPSTFK

TLR6MM FTYSETALKSLMIEHVKNQVFLFSKEALYSVFAEMNIKMLSISDTPFIHMVCPPSPSSFT

TLR6RN FIYVETVLKSLKIEHVTNQVFLFVKDALYSVFAEMNIRMLTLSDTPFIHMVCPEFPSTFA

TLR6EE FTYSKTSLKALKIEHITNRVFLFSQTALYTVFSEMNIMMLTISEAPLIHMLCPQAPSMFK

TLR6BT FTYYKTTLKALKIEHITNKVFIFSQTALYTVFSEMNILMLTISDTRFIHMLCPQEPSTFK

TLR6SS FIYYETTLKGVKIEHITKRVFIFSQTALYRVFSDMNIRMLTIADTHFIHMLCPQVPSTFN

TLR6CF IHYPKTALKALTIEHVKNEVFLFSQTALYTIFSEMNIMMLTISDTPFIHMLCPPPSNTFK

--------||---------LRR11--------||---------LRR12--------||--

370 380 390 400 410 420

| | | | | |

TLR1HS HLDFSNNLLTDTVFENCGHLTELETLILQMNQLKELSKIAEMTTQMKSLQQLDISQNSVS

TLR1PT HLDFSNNLLTDTVFENCGHLTELETLILQMNQLKELSKIAEMTTQMKSLQQLDISQNSVS

TLR1Ma HLDFSNNLLTDTVFENCGHLTELETLILQMNQLKELSKIAEMTTRMKSLQQLDISQNSVS

TLR1MM HVDFTDNLLTDMVFKDCRNLVRLKTLSLQKNQLKNLENIILTSAKMTSLQKLDISQNSLR

TLR1RN YLDFTDNLLTDIVFEDCRNLIRLKTLSLQKNQLKTLENIILMSMEMTSLQKLDISQNSLR

TLR1EE YLDFSNNLLTDMIFKDCGNLTKLETLILQMNQLQEFTKIVYMTKKMKSLQLLDISQNSLR

TLR1BT YLNFSNNLLTDTVFINCTNLANLKTLILQKNQLKELVNIVHMTQEMKSLQQLDVSQNSLM

TLR1SS YLDFSNNALTDMVFKNCANLANLNTLSLQMNQLKELVNVIHMTKEMQSLQQLDVSQNTLR

TLR1CF HLDFSNNLLTDIVFKNCRNLIKLETLSLQMNQLKELASIAQMTNEMKSLQQLDISQNSLR

TLR6HS FLNFTQNVFTDSIFEKCSTLVKLETLILQKNGLKDLFKVGLMTKDMPSLEILDVSWNSLE

TLR6PT FLNFTQNVFTDSIFEKCSTLVKLETLILQKNGLKDLFKVGLMTKDMPSLEILDVSWNSLE

TLR6Ma FLNFTQNVFTDSIFEKCSTLVKLETLILQKNGLKDLFKVGLMTKDMPSLEILDVSWNSLE

TLR6MM FLNFTQNVFTDSVFQGCSTLKRLQTLILQRNGLKNFFKVALMTKNMSSLETLDVSLNSLN

TLR6RN FLNFTQNVFTDSIFQGCSTLKRLETLILQRNGLKNLFKVALMTKTMSSLETLDVSLNSLN

TLR6EE FLNFTHNVFTDSIFQNCSTLGRLETLILQKNELKELFKVGLMTKDMQSLEILDVSWNSLN

TLR6BT FLNFTQNSFTDSVFQNCDTLARLETLILQKNELKDLFKTSLMTKDMLSLETLDVSWNSLE

TLR6SS FLNFTQNVFTDSVFQNCKTLARLETLILQKNKLEDLFKISLMTKDMLSLEILDVSSNSLE

TLR6CF FLNFTQNVFTDSVFQSCSHLVRLETLILRKNKLKDLYKVGLMTKHMTSLEILDVSVNSLE

------LRR13---------||---------LRR14----------||------------

430 440 450 460 470 480

| | | | | |

TLR1HS YDEKKGDCSWTKSLLSLNMSSNILTDTIFRCLPPRIKVLDLHSNKIKSIPKQVVKLEALQ

TLR1PT YDEKKGDCSWTKSLLSLNMSSNILTDTIFRCLPPRIKVLDLHSNKIKSVPKQVVKLEALQ

TLR1Ma YDEKKGDCSWTKSLLSLNMSSNILTDTIFKCLPPRIKVLDLHSNKIKSIPKQVIKLEALQ

TLR1MM YSDGGIPCAWTQSLLVLNLSSNMLTGSVFRCLPPKVKVLDLHNNRIMSIPKDVTHLQALQ

TLR1RN YSDAGSPCSWTQSLLVLNLSSNMLTDSVFRCLPPKVKVLDLHNNRIVSISKDVTHLQALQ

TLR1EE IDENEGNCSWTESLSSLNLSSNILTESVFRCLPPRVKVLDLHSNRIRSIPRDVNNLEALQ

TLR1BT YDESEGNCPWARNLLSLNMSSNILTDSVFRCLPPQIKVLDLHNNRIRSIPKDVTGLETLQ

TLR1SS YDENEGSCTWTGSLLSLNLSSNILTDSVFRCLPPRIKVLDLHNNRIRSIPKDVAHLEALQ

TLR1CF YDENEGNCSWTRSLLSLNMSSNILTDSVFRCLPPKVKVLDLHDNRIRSIPKPIMKLEDLQ

TLR6HS SGRHKENCTWVESIVVLNLSSNMLTDSVFRCLPPRIKVLDLHSNKIKSVPKQVVKLEALQ

TLR6PT SGRHKENCTWVESIVVLNLSSNMLTDSVFRCLPPRIKVLDLHSNKIKSVPKQVIKLKALQ

TLR6Ma SGRHRENCTWVESIVVLNLSSNILTDSVFRCLPPRIKVLDLHNNKIKSIPKQVVKLEALQ

TLR6MM SHAYDRTCAWAESILVLNLSSNMLTGSVFRCLPPKVKVLDLHNNRIMSIPKDVTHLQALQ

TLR6RN SHVYDRTCAWAESIRVLNLSSNVLSDSVFRCLPPKVKVLDLHNNRIVSIPKDVTHLQALQ

TLR6EE YDRYDGICTWAQSIVMLNLSSNILTESVFRCLPPRVKVLDLHSNRIRSIPRDVNNLEALQ

TLR6BT YDRSNGNCSWVGSIVVLNLSSNALTDSVFRCLPPRIKVLDLHNNRIRSIPKDVTGLETLQ

TLR6SS YDRHGENCTWVGSIVVLNLSSNILTDSVFRCLPPRIKVLDLHSNRIRSIPKDVAHLEALQ

TLR6CF YDRYDGNCTWVGSIVVLNLSSNILTDSVFRCLPPKVKVLDLHDNRIRSIPKPIMKLEDLQ

--LRR15----||--------LRR16-------||--------LRR17--------||--

490 500 510 520 530 540

| | | | | |

TLR1HS ELNVAFNSLTDLPGCGSFSSLSVLIIDHNSVSHPSADFFQSCQKMRSIKAGDNPFQCTCE

TLR1PT ELNVAFNSLTDLPGCGSFSSLSVLIIDHNSVSHPSADFFQSCQKMRSIKAGDNPFQCTCE

TLR1Ma ELNVAFNSLTDLPGCGSFSSLSVLIIDHNSVSHPSADFFQSCQKMRSIKAGNNPFQCTCE

TLR1MM ELNVASNSLTDLPGCGAFSSLSVLVIDHNSVSHPSEDFFQSCQNIRSLTAGNNPFQCTCE

TLR1RN ELNVASNFLTDLPGCGAFSSLSVLVIDHNSVSHPSSDFFQSCQNIRSITAGNNPFRCTCE

TLR1EE VLNVASNFLTNLPGCGAFSSLSALIIDYNSISSPSVDFFQSCQNIRSVKAGNNPFQCTCE

TLR1BT ELNLASNSLAHLPGCGIFSSLSILIIDYNSISNPSADFFQSCQKIRSLKAGNNPFQCSCE

TLR1SS ELNVASNSLAHLPGCGSFSSLSILIIDYNSISNPSADFFQSCQKIRSLKAGNNPFQCTCE

TLR1CF ELNVASNSLAHFPDCGTFNRLSVLIIDSNSISNPSADFLQSCHNIRSISAGNNPFQCTCE

TLR6HS ELNVAFNSLTDLPGCGSFSSLSVLIIDHNSVSHPSADFFQSCQKMRSIKAGDNPFQCTCE

TLR6PT ELNVAFNSLTDLPGCGSFSSLSVLIIDHNSVSHPSADFFQSCQKMRSIKAGDNPFQCTCE

TLR6Ma ELNVAFNSLTDLPGCGSFSSLSVLIIDHNSVSHPSADFFQSCQKMRSIKAGNNPFQCTCE

TLR6MM ELNVASNSLTDLPGCGAFSSLSVLVIDHNSVSHPSEDFFQSCQNIRSLTAGNNPFQCTCE

TLR6RN ELNVASNFLTDLPGCGAFSSLSVLVIDHNSVSHPSSDFFQSCQNIRSITAGNNPFRCTCE

TLR6EE VLNVASNFLTNLPGCGAFSSLSALIIDYNSISSPSVDFFQSCQNIRSVKAGNNPFQCTCE

TLR6BT ELNLASNSLAHLPGCGIFSSLSILIIDYNSISNPSADFFQSCQKIRSLKAGNNPFQCSCE

TLR6SS ELNVASNSLAHLPGCGSFSSLSILTIDYNSISNPSADFFQSCQKIRSLKAGNNPFQCTCE

TLR6CF ELNVASNSLAHFPDCGTFNRLSVLIIDSNSISNPSADFLQSCHNIRSMSAGNNPFQCTCE

--------LRR18-----||---------LRR19--------||----------------

550 560 570 580 590 600

| | | | | |

TLR1HS LGEFVKNIDQVSSEVLEGWPDSYKCDYPESYRGTLLKDFHMSELSCNITLLIVTIVATML

TLR1PT LREFVKNIDQVSSEVLEGWPDSYKCDYPESYRGSPLKDFHMSELSCNITLLIVTIVATML

TLR1Ma LREFIKNIEQVSSEVVEGWPDSYKCDYPESYRGTPLKDFHMSELSCNITLLIVTIGATML

TLR1MM LRDFVKNIGWVAREVVEGWPDSYRCDYPESSRGTALRDFHMSPLSCDTVLLTVTIGATML

TLR1RN LREFVKNIGQASREVVEGWPDSYRCDYPDSIKGTPLQDFHMSPLSCDTILLTVTIGATLL

TLR1EE LREFVQRMGQVSREVVEDWPGSYQCDYPESFKGTALKDFHMSQLSCNTTLLIVTIVVIVL

TLR1BT LRDFIQSIGQVSSDVVEGWPESYKCDYPESYKGTPLKDFQVSELSCNTALLIVTIVVPGL

TLR1SS LRDFIQSLGQVSSDVVESWPDSYECEYPESYKGTLLKDFRVSELSCNTALLIVTIGVTGL

TLR1CF LREFVQSLGQVASKVVEGWPDSYKCDSPENYKGTLLKDFHVSPLSCNTTLLLVTIGVAVL

TLR6HS LREFVKNIDQVSSEVLEGWPDSYKCDYPESYRGSPLKDFHMSELSCNITLLIVTIGATML

TLR6PT LRQFVKSIDQVSSEVLEGWPDSYKCDYPESYRGTPLKDFHMSELSCNITLLIITIGATML

TLR6Ma LREFVKNIEQVSSEVVEGWPDSYKCDYPESYRGTPLKDFHMSELSCNITLLIITIGATML

TLR6MM LRDFVKNIGWVAREVVEGWPDSYRCDYPESSKGTALRDFHMSPLSCDTVLLTITIGATML

TLR6RN LREFVKNIGQASREVVEGWPDSYRCDYPDSIKGTPLQDFHMSPLSCDTILLTVTIGATLL

TLR6EE LREFVQRMGQVSREVVENWPGSYQCDYPESFKGTALKDFHMSQLSCNTTLLIVTIVVIVL

TLR6BT LRDFIQSVGQVSSDVVEGWPESYKCDYPESYKGTPLKDFQVSELSCNTALLIVTIVVPGL

TLR6SS LRDFIQSLGQVSSDVVESWPDSYECEYPESYKGTLLKDFRVSELSCNTALLIVTIGVTGL

TLR6CF LREFVQSLGQVASKVVEGWPDSYKCDSPENYKGTLLKDFHVSPLSCNTTLLLVTIGVAVL

----------------C-cap---------------------------||----trans-

610 620 630 640 650 660

| | | | | |

TLR1HS VLAVTVTSLCSYLDLPWYLRMVCQWTQTRRRARNIPLEELQRNLQFHAFISYSGHDSFWV

TLR1PT VLAVTVTSLCIYLDLPWYLRMVCQWTQTRRRARNIPLEELQRNLQFHAFISYSGHDSFWV

TLR1Ma VLAVTVTFLCIYLDLPWYLRMVCQWTQTRRRARNVPLEELQRNLQFHAFISYSGHDSFWV

TLR1MM VLAVTGAFLCLYFDLPWYVRMLCQWTQTRHRARHIPLEELQRNLQFHAFVSYSGHDSAWV

TLR1RN LLAAIGASLCLYFDLPWYLRMLWQWTQTRHRARNIPLEELQRNLQFHAFVSYSGHDSAWV

TLR1EE VLGSTTVMLCIYFDVLWYLRMMCHWTQTRQRARNTPLAELQRNLQFHAFISYSEHDSAWV

TLR1BT VLAVAVTVLCIYLDLPWYLRMVCQWTQTRRRARNVPLEELQRTLQFHAFISYSGHDSAWV

TLR1SS ALALTMTGLCVYFDLPWYLRMLCQWTQTRRRARNVPLEELQRTLQFHAFISYSGHDSAWV

TLR1CF VFTVTVTALCIYFDLPWYLRMVFQWTQTRRRARNTPLENLQRTIQFHAFISYSGHDSAWV

TLR6HS VLAVTVTSLCIYLDLPWYLRMVCQWTQTRRRARNIPLEELQRNLQFHAFISYSEHDSAWV

TLR6PT VLAVTVTSLCIYLDLPWYLRMVCQWTQTRRRARNIPLEELQRNLQFHAFISYSEHDSAWV

TLR6Ma VLAVTVTFLCIYLDLPWYLRMVCQWTQTRHRARNVPLEELQRNLQFHAFISYSEHDSAWV

TLR6MM VLAVTGAFLCLYFDLPWYVRMLCQWTQTRHRARHIPLEELQRNLQFHAFVSYSEHDSAWV

TLR6RN LLAAIGASLCLYFDLPWYLRMLWQWTQTRHRARNIPLEELQRNLQFHAFVSYSEHDSAWV

TLR6EE VLGSTTVMLCIYFDVLWYLRMMCHWTQTRQRARNTPLAELQRNLQFHAFISYSEHDSAWV

TLR6BT VLAVAVTVLCIYLDLPWYLRMVCQWTQTRRRARNVPLEELQRTLQFHAFISYSEHDSAWV

TLR6SS ALALTMTGLCVYFDLPWYLRMLCQWTQTRRRARNVPLEELQRTLQFHAFISYSEHDSAWV

TLR6CF VFTVTVTALCIYFDLPWYLRMVFQWTQTRRRARNTPLEELQRTIQFHAFISYSEHDSAWV

-membrane---||--------TIR domain------>

670 680 690 700 710 720

| | | | | |

TLR1HS KNELLPNLEKEGMQICLHERNFVPGKSIVENIITCIEKSYKSIFVLSPNFVQSEWCHYEL

TLR1PT KNELLPNLEKEGMQICLHERNFVPGKSIVENIITCIEKSYKSIFVLSPNFVQSEWCHYEL

TLR1Ma KNELLPNLEKEGMQICLHERNFVPGKSIVENIINCIEKSYKSIFVLSPNFVQSEWCHYEL

TLR1MM KNELLPNLEKDDIQICLHERNFVPGKSIVENIINFIEKSYKSIFVLSPHFIQSEWCHYEL

TLR1RN KNELLPNLEKDDIRVCLHERNFVPGKSIVENIIHFIEKSYKSIFVLSPHFIQSEWCHYEL

TLR1EE KSELLPNLEKENIRICLHERNFVPGKSIIENIINCIEKSYKSIFILSPNFVQSEWCHYEL

TLR1BT KNELIPNLEKEDIRICLHERNFVAGKSIVENIINCIEKSYKSIFVLSPNFVQSEWCHYEL

TLR1SS KNELLPNVEKEGIKICLHERNFVPGKSIMENIINCIEKSYKSIFVLSPNFVQSEWCHYEL

TLR1CF KSELLPNLEKEELRICLHERNFIPGKSIVENIINCIEKSYKSIFVLSPNFVQSEWCHYEL

TLR6HS KSELVPYLEKEDIQICLHERNFVPGKSIVENIINCIEKSYKSIFVLSPNFVQSEWCHYEL

TLR6PT KTELVPYLEKEDIQICLHERNFVPGKSIVENIINCIEKSYKSIFVLSPNFVQSEWCHYEL

TLR6Ma KNELVPYLEKEGMQVCLHERNFVPGKSIVENIINCIEKSYKSIFVLSPNFVQSEWCHYEL

TLR6MM KNELLPNLEKDDIRVCLHERNFVPGKSIVENIINFIEKSYKAIFVLSPHFIQSEWCHYEL

TLR6RN KNELLPNLEKDDIRVCLHERNFVPGKSIVENIIHFIEKSYKSIFVLSPHFIQSEWCHYEL

TLR6EE KNELVPCLEKENIRICLHERNFVPGKSIIENIINCIEKSYKSIFVLSPNFVQSEWCHYEL

TLR6BT KNELIPNLEKEDIRICLHERNFVAGKSIVENIINCIEKSYKSIFVLSPNFVQSEWCHYEL

TLR6SS KNELVPCLEKEGIKICLHERNFVPGKSIMENIINCIEKSYKSIFVLSPNFVQSEWCHYEL

TLR6CF KNELVPCLEKEELRICLHERNFIPGKSIVENIINCIEKSYKSIFVLSPNFVQSEWCHYEL

730 740 750 760 770 780

| | | | | |

TLR1HS YFAHHNLFHEGSNSLILILLEPIPQYSIPSSYHKLKSLMARRTYLEWPKEKSKRGLFWAN

TLR1PT YFAHHNLFHEGSNNLILILLEPIPQYSIPSSYHKLKSLMARRTYLEWPKEKSKRGLFWAN

TLR1Ma YFAHHNLFHEGSNNLILILLEPIPQYSIPSSYHKLKNLMARRTYLEWPKEKSKHGLFWAN

TLR1MM YFAHHNLFHEGSDNLILILLAPIPQYSIPTNYHKLKTLMSRRTYLEWPTEKNKHGLFWAN

TLR1RN YFAHHNLFHEGSDNLILILLEPIPQYSIPTNYHKLKTLMARRTYLEWPTEKSKHGLFWAN

TLR1EE YFAHHNLFHKGSDNLILILLEPIPQYSIPNSYHKLKALMARRTYLEWPKEKRKHGLFWAS

TLR1BT YFAHHNLFHEGSDNLILILLDPIPQYSIPSSYHKLRALMAQRTYLEWPKEKSKHGLFWAN

TLR1SS YFAHHNLFHEGSDNLILILLDSIPQYSIPSSYHKLKALMAQRTYLEWPKEKSKHGLFWAN

TLR1CF YFAHHNLFHEGSNNLILILLEPIPQYSIPSSYHKLKNLMAQRTYLEWPKEKSKHGLFWAN

TLR6HS YFAHHNLFHEGSNNLILILLEPIPQNSIPNKYHKLKALMTQRTYLQWPKEKSKRGLFWAN

TLR6PT YFAHHNLFHEGSNNLILILLEPIPQNSIPNKYHKLKALMMQRTYLQWPKEKSKRGLFWAN

TLR6Ma YFAHHNLFHEGSNNLILILLEPIPQNSIPNKYHKLRALMTQRTYLQWPKEKSKRGLFWAN

TLR6MM YFAHHNLFHEGSDNLILILLEPILQNNIPSRYHKLRALMAQRTYLEWPTEKGKRGLFWAN

TLR6RN YFAHHNLFHEGSDNLILILLEPIQQNNIPSRYHKLRALMAQRTYLEWPIEKGKRGLFWAN

TLR6EE YFAHHNLFHEGSDNLILILLEPIPQNNIPSKYHKLKALMTQRTYLEWPKEKSKHGLFWAN

TLR6BT YFAHHNLFHEGSNNLILILLDPIPQNTIPDRYHKLRALMAQRTYLEWPKEKNKHGLFWAN

TLR6SS YFAHHNLFHEGSDNLILILLDPIPQNSIPGKYHKLKALMAQRTYLEWPKEKSKHGLFWAN

TLR6CF YFAHHNLFHEGSNNLILILLEPIPQNCIPSKYHKLRALMTQRTYLEWPKEKSKHGLFWAN

790

|

TLR1HS LRAAINIKLTEQAKK-

TLR1PT LKAAINIKLTEQAKK-

TLR1Ma LRAAINIKLTEQAKK-

TLR1MM LRASINVKLVNQAEGTCYTQQ-

TLR1RN LRASINVKLVNQAEATCYTQQ-

TLR1EE LRVSINIKLTEQAKEVCHTQIQNILTTSAF-

TLR1BT LRASINIKLMEKAAEIH-

TLR1SS LRASINIKLMEKAEEISYTQI-

TLR1CF LRASINIKLREQAKK-

TLR6HS IRAAFNMKLTLVTENNDVKS-

TLR6PT IRAAFNMKLTLVTENNDVKS-

TLR6Ma IRATFNVKLTLVTENNDVKS-

TLR6MM LRASFIMKLALV-NEDDVKT-

TLR6RN LRASFIMKLALV-NENDVKT-

TLR6EE ITAAFHMKLTLV-NENDAET-

TLR6BT IRAAFNIKLRLVTENDDVKG-

TLR6SS IRAAFNIKLKLVAEEDDVKT-

TLR6CF IRAAFNMKLTLIAENNNAEAS-
