# Supplementary material for: Gene conversion limits divergence of mammalian TLR1 and TLR6
Source: BMC Evol Biol. 2007 Aug 29;7:148. doi: 10.1186/1471-2148-7-148 (PMC2077338; doi:10.1186/1471-2148-7-148)
Supplement: Additional file 2 — Sequence similarity of partial and full length sequences of TLR1 and TLR6 from six mammalian species. Sequences of TLR1 and TLR6 from six mammalian species representing five mammalian orders were analysed: Homo sapiens (HS1 and HS6), Pan troglodytes (PT1 and PT6), Macaca mulatta (Ma1 and Ma6), Mus musculus (MM1 and MM6), Rattus norvegicus (RN1 and RN6), Erinaceus europaeus (EE1 and EE6), Bos taurus (BT1 and BT6), Sus scrofa (SS1 and SS6) and Canis familiaris (CF1 and CF6). Pairwise percentage identities were calculated for all full length protein sequences and for the regions N-termini to amino acid 436, amino acids 437 to 744 and amino acid 745 to C-termini. Average results for percentage identities between orthologs and paralogs, as well as statistics, are given in section A). Individual results for comparisons between TLR1 and TLR6 orthologs (red and blue, respectively; n = 15), and the TLR1/TLR6 paralogs (green; n = 6), are given in B). [file 1471-2148-7-148-S2.doc]

# Additional file 2

**Sequence similarity of partial and full length sequences of TLR1 and TLR6 from six mammalian species**

Sequences of TLR1 and TLR6 from six mammalian species were analysed: *Homo sapiens* (HS1 and HS6), *Mus musculus* (MM1 and MM6), *Erinaceus europaeus* (EE1 and EE6), *Bos taurus* (BT1 and BT6), *Sus scrofa* (SS1 and SS6) and *Canis familiaris* (CF1 and CF6). Pairwise percentage homologies were calculated for all full length protein sequences and for the regions N-termini to amino acid 436, amino acids 437 to 744 and amino acid 745 to C-termini. Average results for homologies between orthologs and paralogs, as well as statistics, are given in section A). Individual results for comparisons between TLR1 and TLR6 orthologs (red and blue, respectively; n= 15), and the TLR1/TLR6 paralogs (green; n=6), are given in B).

**A) Average (± SD) percentage sequence identity**

|  | Full length | Region 1 – 436 | Region 437 – 744 | Region 745 - end |
| --- | --- | --- | --- | --- |
| a) TLR1 orthologs | 75.5 ± 4.5 | 71.6 ± 5.7 | 79.7 ± 3.3 | 81.1 ±7.6 |
| b) TLR6 orthologs | 75.2 ± 4.1 | 71.5 ± 5.1 | 80.3 ± 3.0 | 74.7 ± 5.2 |
| c) TLR1/TLR6 paralogs | 66.8 ± 2.1 | 46.0 ± 3.4 | 96.7 ± 1.4 | 65.2 ± 7.3 |
| p values a) vs b) | > 0.94 | > 0.94 | 0.419 | 0.025 |
| p values a) vs c) | 0.00011 | < 0.0001 | < 0.0001 | 0.0003 |
| p values b) vs c) | < 0.0001 | < 0.0001 | < 0.0001 | 0.01 |

N.B. p values were calculated using the Wilcoxon two sample test

**B) Individual results**

**Full length**

HS1 100

HS6 68 100

MM1 73 60 100

MM6 59 73 64 100

EE1 73 60 69 57 100

EE6 60 76 57 70 65 100

BT1 78 62 70 58 74 61 100

BT6 64 80 59 71 60 76 69 100

SS1 78 62 72 59 74 61 85 66 100

SS6 63 79 59 70 60 74 65 84 69 100

CF1 79 61 71 56 75 59 80 63 81 62 100

CF6 61 77 57 70 58 74 61 78 61 76 66

HS1 HS6 MM1 MM6 EE1 EE6 BT1 BT6 SS1 SS6 CF1

**region start 1-436**

HS1 100

HS6 50 100

MM1 68 44 100

MM6 44 69 42 100

EE1 69 46 63 43 100

EE6 47 74 43 63 43 100

BT1 75 48 64 43 69 46 100

BT6 51 78 46 67 45 73 49 100

SS1 76 48 66 44 69 46 80 50 100

SS6 49 76 46 65 45 68 46 81 48 100

CF1 78 48 68 42 72 45 77 49 80 47 100

CF6 48 75 43 67 44 70 45 75 45 71 44

HS1 HS6 MM1 MM6 EE1 EE6 BT1 BT6 SS1 SS6 CF1

**Region 437 to 744**

HS1 100

HS6 94 100

MM1 80 81 100

MM6 78 80 97 100

EE1 77 79 77 78 100

EE6 78 79 78 78 98 100

BT1 82 82 78 77 79 80 100

BT6 81 83 77 77 80 80 97 100

SS1 81 81 79 78 80 80 89 88 100

SS6 80 82 78 78 80 82 88 88 97 100

CF1 78 79 75 75 77 77 82 81 81 80 100

CF6 78 79 75 76 77 79 81 82 81 82 97

HS1 HS6 MM1 MM6 EE1 EE6 BT1 BT6 SS1 SS6 CF1

**Region 745 to end**

HS1 100

HS6 70 100

MM1 74 56 100

MM6 64 66 57 100

EE1 84 58 71 55 100

EE6 66 79 53 74 55 100

BT1 82 63 69 67 78 63 100

BT6 66 78 56 71 58 74 69 100

SS1 84 61 73 64 80 62 94 63 100

SS6 72 80 56 67 63 75 73 81 72 100

CF1 90 66 76 66 84 66 88 70 90 74 100

CF6 68 80 57 67 55 81 69 74 66 74 68

HS1 HS6 MM1 MM6 EE1 EE6 BT1 BT6 SS1 SS6 CF1
