# Supplementary figures and images for: Gene conversion limits divergence of mammalian TLR1 and TLR6
Source: BMC Evol Biol. 2007 Aug 29;7:148. doi: 10.1186/1471-2148-7-148 (PMC2077338; doi:10.1186/1471-2148-7-148)

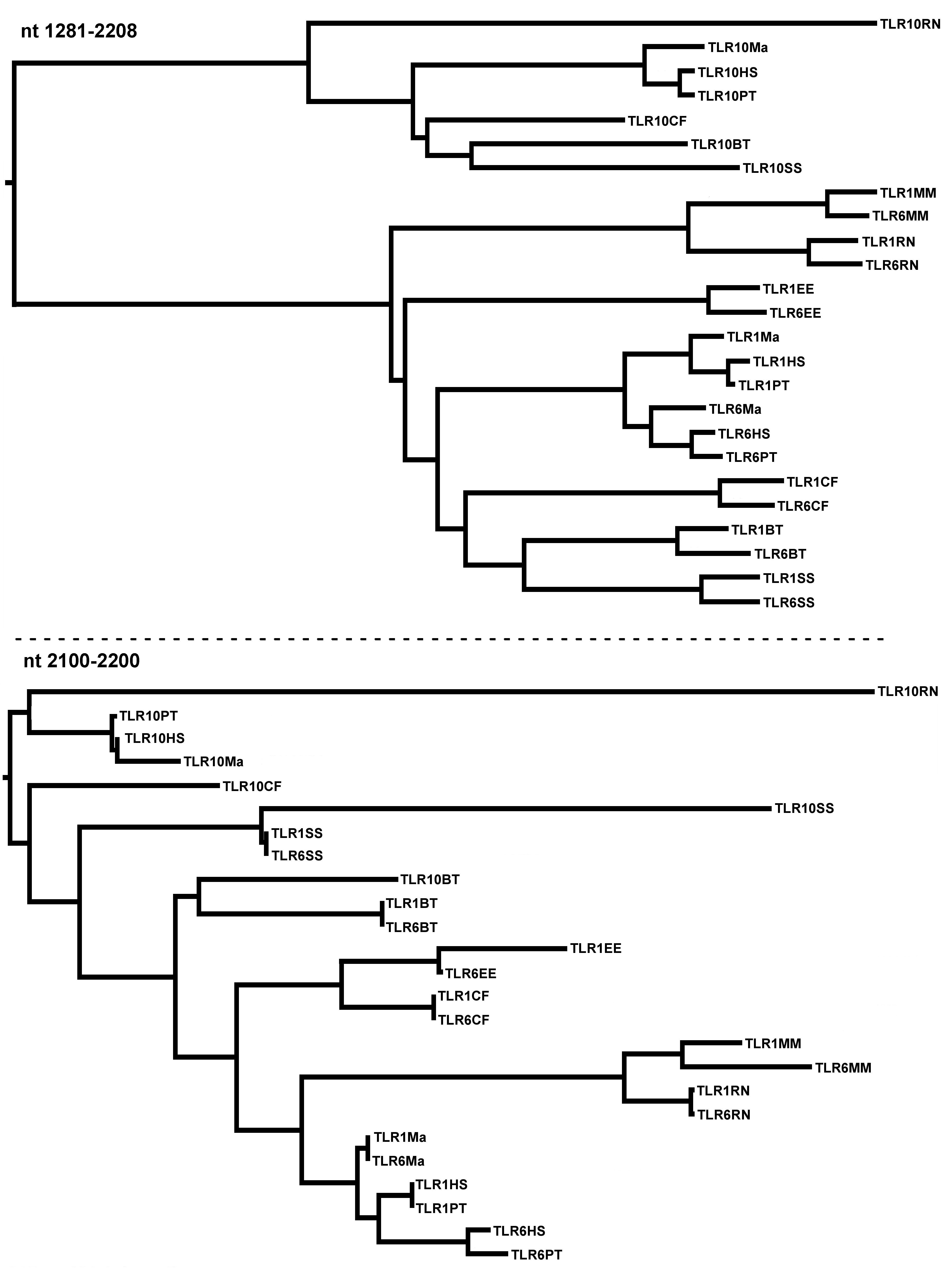

Supplement: Additional file 3 — Phylogenetic trees of mammalian TLR1, TLR6 and TLR10. Top: phylogenetic tree of DNA encoding the region containing LRR16 to the N-terminal three quarters of the TIR domain (nucleotide 1282–2208). Note that for this segment the paralog TLR1 and TLR6 sequences cluster together for all non-primate sequences and are separate from the clustered TLR10 sequences. Bottom: For the small nucleotide region 2100 – 2200 the paralog TLR1, TLR6 and TLR 10 sequences cluster together, but only for bovine and swine sequences. [file 1471-2148-7-148-S3.jpeg]
