# Supplementary material for: Gene conversion limits divergence of mammalian TLR1 and TLR6
Source: BMC Evol Biol. 2007 Aug 29;7:148. doi: 10.1186/1471-2148-7-148 (PMC2077338; doi:10.1186/1471-2148-7-148)
Supplement: Additional file 4 — DNA and protein sequences for TLR1 and TLR6 used for analyses. TLR1 and TLR6 sequences were analysed from nine mammalian species: Homo sapiens (HS), Pan troglodytes (PT), Macaca mulatta (Ma) (representing the order Primates); Mus musculus (MM) and Rattus norvegicus (RN) (representing the order Rodentia); Erinaceus europaeus (EE) (representing the order Insectivora), Bos taurus (BT) and Sus scrofa (SS) (representing two families of the order Artiodactyla) and Canis familiaris (CF) (representing the order Carnivora). All sequences were obtained from the Ensembl genome browser [28], except the Sus scrofa sequences which were obtained from the NCBI nucleotide server [29]. The open reading frame start and stop codons are underlined in the DNA sequences. A DNA sequence alignment is given, with identical nucleotides marked with an * at the bottom of the alignment. In the protein sequences blue-yellow and yellow-purple highlights indicate the boundaries between the regions 1 to 436, 437 to 745 and 746 to end. Protein alignments are given in additional file 1. [file 1471-2148-7-148-S4.doc]

# Additional file 4

**DNA and protein sequences for TLR1 and TLR6 used for analyses**

TLR1 and TLR6 sequences were analysed from nine mammalian species: *Homo sapiens* (HS), *Pan troglodytes* (PT), *Macaca mulatta* (Ma) (representing the order Primates); *Mus musculus* (MM) and *Rattus norvegicus* (RN)(representing the order Rodentia); *Erinaceus europaeus* (EE) (representing the order Insectivora), *Bos taurus* (BT) and *Sus scrofa* (SS) (representing two families of the order Artiodactyla) and *Canis familiaris* (CF) (representing the order Carnivora). TLR10 sequences were from the same species except *Mus Musculus* that has a TLR10 pseudogene and *Erinaceus europaeus* for which no TLR10 sequence is presently available. All sequences were obtained from the Ensembl genome browser, release 44 <http://www.ensembl.org/index.html>, except the *Sus scrofa* sequences which were obtained from the NCBI nucleotide server: <http://www.ncbi.nlm.nih.gov/entrez/query.fcgi?CMD=search&DB=nucleotide>. The open reading frame start and stop codons are underlined in the DNA sequences. A DNA sequence alignment is given for TLR1 and TLR6, with identical nucleotides marked with an * at the bottom of the alignment. In the protein sequences of TLR1 and TLR6 blue-yellow and yellow-purple highlights indicate the boundaries between the regions 1 to 436, 437 to 745 and 746 to end. Protein alignments for TLR1 and TLR6 are given in additional file 1.

>TLR1HS

ATGACTAGCATCTTCCATTTTGCCATTATCTTCATGTTAATACTTCAGATCAGAATACAATTATCTGAAGAAAGTGAATTTTTAGTTGATAGGTCAAAAAACGGTCTCATCCACGTTCCTAAAGACCTATCCCAGAAAACAACAATCTTAAATATATCGCAAAATTATATATCTGAGCTTTGGACTTCTGACATCTTATCACTGTCAAAACTGAGGATTTTGATAATTTCTCATAATAGAATCCAGTATCTTGATATCAGTGTTTTCAAATTCAACCAGGAATTGGAATACTTGGATTTGTCCCACAACAAGTTGGTGAAGATTTCTTGCCACCCTACTGTGAACCTCAAGCACTTGGACCTGTCATTTAATGCATTTGATGCCCTGCCTATATGCAAAGAGTTTGGCAATATGTCTCAACTAAAATTTCTGGGGTTGAGCACCACACACTTAGAAAAATCTAGTGTGCTGCCAATTGCTCATTTGAATATCAGCAAGGTCTTGCTGGTCTTAGGAGAGACTTATGGGGAAAAAGAAGACCCTGAGGGCCTTCAAGACTTTAACACTGAGAGTCTGCACATTGTGTTCCCCACAAACAAAGAATTCCATTTTATTTTGGATGTGTCAGTCAAGACTGTAGCAAATCTGGAACTATCTAATATCAAATGTGTGCTAGAAGATAACAAATGTTCTTACTTCCTAAGTATTCTGGCGAAACTTCAAACAAATCCAAAGTTATCAAATCTTACCTTAAACAACATTGAAACAACTTGGAATTCTTTCATTAGGATCCTCCAGCTGGTTTGGCATACAACTGTATGGTATTTCTCAATTTCAAACGTGAAGCTACAGGGTCAGCTGGACTTCAGAGATTTTGATTATTCTGGCACTTCCTTGAAGGCCTTGTCTATACACCAAGTTGTCAGCGATGTGTTCGGTTTTCCGCAAAGTTATATCTATGAAATCTTTTCGAATATGAACATCAAAAATTTCACAGTGTCTGGTACACGCATGGTCCACATGCTTTGCCCATCCAAAATTAGCCCGTTCCTGCATTTGGATTTTTCCAATAATCTCTTAACAGACACGGTTTTTGAAAATTGTGGGCACCTTACTGAGTTGGAGACACTTATTTTACAAATGAATCAATTAAAAGAACTTTCAAAAATAGCTGAAATGACTACACAGATGAAGTCTCTGCAACAATTGGATATTAGCCAGAATTCTGTAAGCTATGATGAAAAGAAAGGAGACTGTTCTTGGACTAAAAGTTTATTAAGTTTAAATATGTCTTCAAATATACTTACTGACACTATTTTCAGATGTTTACCTCCCAGGATCAAGGTACTTGATCTTCACAGCAATAAAATAAAGAGCATTCCTAAACAAGTCGTAAAACTGGAAGCTTTGCAAGAACTCAATGTTGCTTTCAATTCTTTAACTGACCTTCCTGGATGTGGCAGCTTTAGCAGCCTTTCTGTATTGATCATTGATCACAATTCAGTTTCCCACCCATCGGCTGATTTCTTCCAGAGCTGCCAGAAGATGAGGTCAATAAAAGCAGGGGACAATCCATTCCAATGTACCTGTGAGCTAGGAGAATTTGTCAAAAATATAGACCAAGTATCAAGTGAAGTGTTAGAGGGCTGGCCTGATTCTTATAAGTGTGACTACCCGGAAAGTTATAGAGGAACCCTACTAAAGGACTTTCACATGTCTGAATTATCCTGCAACATAACTCTGCTGATCGTCACCATCGTTGCCACCATGCTGGTGTTGGCTGTGACTGTGACCTCCCTCTGCAGCTACTTGGATCTGCCCTGGTATCTCAGGATGGTGTGCCAGTGGACCCAGACCCGGCGCAGGGCCAGGAACATACCCTTAGAAGAACTCCAAAGAAATCTCCAGTTTCATGCATTTATTTCATATAGTGGGCACGATTCTTTCTGGGTGAAGAATGAATTATTGCCAAACCTAGAGAAAGAAGGTATGCAGATTTGCCTTCATGAGAGAAACTTTGTTCCTGGCAAGAGCATTGTGGAAAATATCATCACCTGCATTGAGAAGAGTTACAAGTCCATCTTTGTTTTGTCTCCCAACTTTGTCCAGAGTGAATGGTGCCATTATGAACTCTACTTTGCCCATCACAATCTCTTTCATGAAGGATCTAATAGCTTAATCCTGATCTTGCTGGAACCCATTCCGCAGTACTCCATTCCTAGCAGTTATCACAAGCTCAAAAGTCTCATGGCCAGGAGGACTTATTTGGAATGGCCCAAGGAAAAGAGCAAACGTGGCCTTTTTTGGGCTAACTTAAGGGCAGCCATTAATATTAAGCTGACAGAGCAAGCAAAGAAATAG

>TLR1PT

ATGCCTAGCATCTTCCATTTTGCCATTATCTTCATGTTAATACTTCAGATCAGAATACAATTATCTGAAGAAAGTGAATTTTTAGTTGATAGGTCAAAAAACGGTCTCATCCACGTTCCTAAAGACCTGTCCCAGAAAACAACAATCTTAAATATATCGCAAAATTATATATCTGAGCTTTGGACTTCTGACATCTTATCACTGTCAAAACTGAGGATTTTGATAATTTCTCATAATAGAATCCAGTATCTTGATATCAGTGTTTTCAAATTCAACCACGAATTGGAATACTTGGATTTGTCCCACAACAAGTTGGTGAAGATTTCTTGCCACCCTACTGTGAACCTCAAGCACTTGGACCTGTCATTTAATGCATTTGATGCCCTGCCTATATGCAAAGAGTTTGGCAATATGTCTCAACTAAAATTTCTGGGGTTGAGCACCACACACTTAGAAAAATCTAGTGTGCTGCCAATTGCTCATTTGAATATCAGCAAGATCTTGCTGGTCTTAGGAGAGACTTATGGGGAAAAAGAAGACCCTGAGGGCCTTCAAGACTTTAACACTGAGAGTCTGCACATTGTGTTCCCCACAAACAAAGAATTCCATTTTATTTTGGATGTGTCAGTCAAGACTGTAGCAAATCTGGAACTATCTAATATCAAATGTGTGCTAGAAGATAACAAATGTTCTTACTTCCTAAGTATTCTGGCGAAACTTCAAACAAATCCAAAGTTATCAAGTCTTACCTTAAACAACATTGAAACAACTTGGAATTCTTTCATTAGGATCCTCCAGCTGGTTTGGCATACAACCGTATGGTATTTCTCAATTTCAAACGTGAAGCTACAGGGTCAGCTGGACTTCAGAGATTTTGATTATTCTGGCACTTCCTTGAAGGCCTTGTCTATACACCAAGTTGTCAGCGATGTGTTCAGTTTTCCGCAAAGTGATATCTATGAAATCTTTTCGAATATGAACATCAAAAATTTCACAGTGTCTGGTACACGCATGGTCCACATGCTTTGCCCATCCAAAATTAGCCCGTTCCTGCATTTGGATTTTTCCAATAATCTCTTAACAGACACGGTTTTTGAAAATTGTGGGCACCTTACTGAGTTGGAGACACTTATTTTACAAATGAATCAATTAAAAGAACTTTCAAAAATAGCTGAAATGACTACACAGATGAAGTCTCTGCAACAATTGGATATTAGCCAGAATTCTGTAAGCTATGATGAAAAGAAAGGAGACTGTTCTTGGACTAAAAGTTTATTAAGTTTAAATATGTCTTCAAATATACTTACTGACACTATTTTCAGATGTTTACCTCCCAGGATCAAGGTACTTGATCTTCACAGCAATAAAATAAAGAGCGTTCCTAAACAAGTCGTAAAACTGGAAGCTTTGCAAGAACTCAATGTTGCTTTCAATTCTTTAACTGACCTTCCTGGATGTGGCAGCTTTAGCAGCCTTTCTGTATTGATCATTGATCACAATTCAGTTTCCCACCCATCGGCTGATTTCTTCCAGAGCTGCCAGAAGATGAGGTCAATAAAAGCAGGGGACAATCCATTCCAATGTACCTGTGAGCTAAGAGAATTTGTCAAAAATATAGACCAAGTATCAAGTGAAGTGTTAGAGGGCTGGCCTGATTCTTATAAGTGTGACTACCCAGAAAGTTATAGAGGAAGCCCACTAAAGGACTTTCACATGTCTGAATTATCCTGCAACATAACTCTGCTGATCGTCACCATCGTTGCCACCATGCTGGTGTTGGCTGTGACTGTGACCTCCCTCTGCATCTACTTGGATCTGCCCTGGTATCTCAGGATGGTGTGCCAGTGGACCCAGACCCGGCGCAGGGCCAGGAACATACCCTTAGAAGAACTCCAAAGAAATCTCCAGTTTCATGCATTTATTTCATATAGTGGGCACGATTCTTTCTGGGTGAAGAATGAATTATTACCAAACCTAGAGAAAGAAGGTATGCAGATTTGCCTTCATGAGAGAAACTTTGTTCCTGGCAAGAGCATTGTGGAAAATATCATCACCTGCATTGAGAAGAGTTACAAGTCCATCTTTGTTTTGTCTCCCAACTTTGTCCAGAGTGAATGGTGCCATTATGAACTCTACTTTGCCCATCACAATCTCTTTCATGAAGGATCTAATAACTTAATCCTGATCTTGCTGGAACCCATTCCACAGTACTCCATTCCTAGCAGTTATCACAAGCTCAAAAGTCTCATGGCCAGGAGGACTTATTTGGAATGGCCCAAGGAAAAGAGCAAACGTGGCCTTTTTTGGGCTAACTTAAAGGCAGCCATTAATATTAAGCTGACAGAGCAAGCAAAGAAATAG

>TLR1Ma

ATGACTAGCATCTTCCATTTTGCCATTATCTTTATGTTAACACTTCAGATCAGAATACAATTATCTGAAGAAAGTGAATTTTTAGTTGATAGGTCAAAAAACAGTCTCATCCACGTTCCTAAAGACCTATCCCAGAAAACAACAATCTTAAATATATCACAAAATTATATATCTGAGCTTTGGACTTCTGACATCTTATCACTGTCAAAGCTGAGGATTTTGATAATTTCTCATAATAGACTCCAGTATCTTGATATCAGTGTTTTCAAATTCAACCAGGAATTGGAATACTTGGATTTGTCCCACAACAAGTTGGCGAAAATTTCTTGCCACCCTACTGTGAACCTCAAGCACTTGGACCTGTCCTTTAATGCATTTGATGCCCTGCCTATATGCAAAGAGTTTGGCAATATGTCTCAACTAAAATTTCTGGGGTTGAGTACTACACACTTAGAAAAATCTACTGTGCTGCCAATTGCTCATTTGAATATCAGCAAGGTCTTGCTGGTCTTAGGAGAGCATTATGGGGACAAAGAAGACCCTGAGGGCCTTCAAAACTTTAACACTGAGAGTCTGCACATTGTGTTCCCAACAAGCAAAGAATTCAATTTTATTTTGGATGTGTCAGTCAGGACTGTAGCAAATCTGGAACTATCTAATATCAAATGTGTGCTAGAAGATAACGAATGTTCTTACTTCCTAAATATTCTGGCAAAACTTCAAACAAATCCAAAGTTATCAAGTCTTACTTTAAACAACATTGAAACAACTTGGAATTCTTTCATTAGGATCCTCCAGCTGGTTTGGCATACAACCGTATGGTATTTCTCAATTTCAAACGTGAAGCTACAGGGTCAACTGGACTTCAGAGATTTTGATTATTCTGGCACTTCCCTGAAGGCCTTGTCTGTACACCAAGTCGTCAGCGATGTGTTCAATTTTCCACAAAGGGATATCTATGAAATCTTTTCAAATATGAACATCAAAAATTTCACAGTGTCTGGTACACGCATGATCCACATGGTTTGCCCATCCAAAATCAGCCCGTTCCTGCATTTGGATTTTTCCAATAATCTCTTAACAGACACGGTTTTTGAAAATTGTGGGCACCTTACTGAGTTGGAGACACTTATTTTACAAATGAATCAATTAAAAGAACTTTCAAAAATAGCTGAAATGACTACACGGATGAAGTCTCTGCAACAATTGGATATTAGCCAGAATTCTGTAAGCTATGATGAAAAGAAAGGAGATTGCTCTTGGACTAAAAGTTTATTAAGTTTAAATATGTCTTCAAATATACTTACTGACACTATTTTCAAATGTTTACCTCCCAGGATCAAGGTACTTGATCTTCACAGCAATAAAATAAAGAGCATTCCTAAACAAGTCATAAAACTGGAAGCTTTGCAAGAACTCAATGTTGCTTTCAATTCTTTAACTGACCTTCCTGGATGTGGCAGCTTTAGCAGCCTTTCTGTATTGATCATTGATCACAATTCAGTTTCCCACCCATCAGCTGATTTCTTCCAGAGCTGCCAGAAGATGAGGTCAATAAAAGCAGGAAACAATCCATTCCAGTGTACCTGTGAGCTAAGAGAATTTATCAAAAATATAGAGCAAGTATCAAGTGAAGTGGTAGAGGGCTGGCCTGATTCTTATAAGTGTGACTACCCAGAAAGTTATAGAGGAACCCCACTAAAGGACTTTCACATGTCTGAATTATCCTGCAACATAACTCTGCTGATCGTCACCATCGGTGCCACCATGCTGGTGTTGGCTGTGACTGTGACCTTCCTCTGCATCTACTTGGATCTGCCCTGGTATCTCAGGATGGTGTGCCAGTGGACCCAGACCCGGCGCAGGGCCAGGAATGTACCCTTAGAAGAACTCCAAAGAAATCTCCAGTTTCATGCATTTATTTCATATAGTGGGCACGATTCTTTCTGGGTGAAGAATGAATTATTACCAAACCTAGAGAAAGAAGGTATGCAGATTTGCCTTCATGAGAGAAACTTTGTTCCTGGCAAGAGCATTGTGGAAAATATCATCAACTGCATTGAGAAGAGTTACAAGTCCATCTTTGTTTTGTCTCCCAACTTTGTCCAGAGTGAGTGGTGCCATTATGAACTCTACTTTGCCCATCACAATCTCTTTCATGAAGGATCTAATAACTTAATCCTGATCTTGCTGGAACCCATTCCGCAGTACTCCATTCCTAGCAGCTATCACAAGCTCAAAAATCTCATGGCCAGGAGGACTTATTTGGAATGGCCCAAGGAAAAGAGCAAACATGGGCTTTTTTGGGCTAATCTAAGGGCAGCCATTAATATTAAGCTGACAGAGCAAGCAAAAAAATAG

>TLR1MM

ATGACTAAACCAAATTCCCTCATCTTCTACTGTATCATTGTTTTAGGACTGACACTTATGAAAATCCAATTATCTGAGGAATGTGAGCTTATCATAAAGAGGCCAAACGCAAACCTTACCAGAGTGCCCAAGGACCTACCCTTGCAAACAACTACTTTAGATCTATCACAAAACAATATATCTGAGCTTCAGACTTCTGACATCCTCTCATTGTCCAAGCTGAGGGTCCTGATAATGTCCTACAACAGACTCCAGTATCTTAATATCAGTGTTTTCAAATTCAACACAGAGCTGGAATATTTGGATTTGTCCCACAATGAGCTAAAGGTGATCTTGTGCCACCCAACAGTCAGCCTCAAGCATTTGGACCTCTCCTTTAATGCCTTTGATGCCCTGCCTATATGCAAAGAATTTGGCAACATGTCCCAACTACAGTTCCTGGGGTTGAGCGGTTCTCGGGTACAAAGTTCAAGTGTGCAGCTGATTGCTCATTTGAACATCAGTAAGGTTTTGCTGGTGTTAGGAGATGCTTATGGGGAAAAAGAAGACCCCGAATCTCTTCGGCACGTTAGCACTGAGACTCTGCATATTGTTTTCCCGTCGAAAAGAGAATTCCGTTTTCTTCTGGATGTGTCCGTCAGCACTACGATCGGTTTGGAACTGTCTAACATCAAGTGTGTGCTTGAAGACCAGGGCTGCTCTTATTTCTTACGTGCTTTGTCAAAGCTTGGAAAGAATCTGAAGCTCTCAAATCTTACCCTGAACAATGTGGAAACAACGTGGAATTCCTTCATTAATATCCTCCAGATAGTTTGGCATACGCCAGTCAAATATTTCTCAATTTCAAATGTGAAGCTACAAGGTCAACTTGCCTTCAGGATGTTCAATTATTCTGACACTTCTCTGAAGGCTTTGTCGATACATCAAGTTGTCACTGATGTCTTCAGCTTCCCCCAAAGTTACATATACAGTATCTTTGCCAATATGAACATCCAAAACTTTACAATGTCTGGAACACACATGGTCCACATGCTGTGCCCGTCCCAAGTTAGCCCATTTCTGCATGTGGACTTTACAGATAACCTTTTAACAGACATGGTTTTTAAAGACTGTAGAAACTTAGTTAGATTGAAAACACTTAGTTTACAAAAGAATCAGTTAAAAAACCTTGAGAATATAATCCTCACATCTGCAAAGATGACATCCCTACAAAAACTAGACATTAGCCAGAATTCTCTAAGGTACAGCGATGGGGGAATCCCATGCGCCTGGACCCAGAGTTTGTTAGTTTTAAATTTGTCTTCGAATATGCTTACAGGCTCTGTCTTCAGATGCTTACCTCCCAAAGTCAAGGTCCTTGACCTTCACAACAACAGGATAATGAGCATCCCTAAAGATGTCACCCACCTGCAGGCTTTGCAGGAACTCAATGTAGCATCCAACTCCTTAACTGACCTTCCTGGGTGCGGGGCCTTCAGCAGCCTTTCTGTGCTGGTCATCGACCATAACTCAGTTTCCCATCCCTCTGAGGATTTCTTCCAGAGCTGTCAGAATATTAGATCCCTAACAGCGGGAAACAACCCATTCCAATGCACATGTGAGCTGAGGGACTTTGTCAAGAACATAGGCTGGGTAGCAAGAGAAGTGGTGGAGGGCTGGCCTGACTCTTACAGGTGTGACTACCCAGAAAGCTCTAGGGGAACTGCACTGAGGGACTTCCACATGTCTCCACTATCCTGTGATACTGTTCTGCTGACTGTCACCATCGGGGCCACTATGCTGGTGCTGGCTGTCACTGGGGCTTTCCTCTGTCTCTACTTTGACCTGCCCTGGTATGTGAGGATGCTGTGTCAGTGGACACAGACCAGGCACAGGGCCAGGCACATCCCCTTAGAGGAACTCCAGAGAAACCTCCAGTTCCATGCTTTTGTCTCATACAGTGGGCATGATTCTGCCTGGGTGAAGAACGAATTACTACCCAACCTAGAGAAAGATGACATCCAGATTTGCCTCCATGAGAGGAACTTTGTCCCTGGCAAGAGCATTGTGGAGAACATCATCAATTTCATTGAGAAGAGTTACAAGTCCATCTTTGTGCTGTCTCCCCACTTCATCCAGAGTGAGTGGTGTCATTATGAACTCTATTTTGCCCATCACAATCTCTTCCATGAAGGCTCTGATAACTTAATCCTCATCTTGCTGGCACCCATTCCCCAGTACTCCATCCCTACCAATTACCACAAGCTCAAAACTCTCATGTCACGAAGGACCTATCTGGAATGGCCCACAGAGAAGAACAAGCATGGACTTTTTTGGGCAAACCTAAGAGCATCCATTAATGTTAAGCTGGTTAACCAGGCAGAAGGAACGTGTTACACACAGCAATAA

>TLR1RN

ATGACTAAAACACAGTCCACCATCTTCTATTGTATTGTTGTCTTAGGGCTGATACTTATCAAAATCCAGTTATCTGAGGAAAGTGAGTTGATCATTAAGAGGCCAAATGCAAACCTTACCAGAGTGCCCAAGGACCTACCCTTGCAAACAACTACTTTAGATGTATCACAAAACAATATATCTGAGCTTCAGACTTCTGACATACTCTTGTTGTCCAAACTGAGGGTCTTCATTATGTCCTACAACAGACTCCAGTATCTTAATATCAGTGTTTTCAAATTCAACACGGAACTGGAATATTTGGATTTGTCCCACAATGAGTTAAGGCTGATCTCTTGCCACGCAACAGCCGACCTCAAACATTTAGACCTCTCCTTTAATGCATTTGATGCCCTGCCCATATGCAAAGAGTTTGGCAACCTGTCCCAACTACAGTTTCTGGGATTGAGCGGTTCTCAGATACAAAATTCAAGTGTGCAGCTGATTGCTCATCTGAACATCAGTAAGGTTTTGCTGGTGTTAGGAGACACTTATGGGGAAAAAGAAGATCCCAAGTGTCTTCAGCACATTAGCACTGAGACTCTGCATATCGTTTTCCCTTCCAAAAGAGAATTCCATTTTCTTCTGGACATGTCTGTCAGCACGGCCATCAGTTTGGAACTGTCTAACATCAAGTGTGTGCTTGAGGACAAGAACTGCTCTTACTTCCTAGGTACCTTAGAAAGACTTAGAAAGACTCAGAGGCTCTCAAATCTTACCCTGAACAACGTGGACACAACATGGAATTCCTTCATTAACATCCTTCAGCTGGTTTGGCATACACCAGTCAAGTCTTTCTCAATTTCAAACGTGAAACTAAAAGGTCATTTTAACTTCAGAAGATTCCATTATTCTGACACTTCTCTGAGGGCTTTGTCGATACATCAAGTTGTCACTGATGTGTTCAGCTTCCCCCAAAGTAACATATACAGCATCTTCTCCAATATGAACATCCAAAGTTTTACAGTATCTGGAACACGCATGGTCCACATGCTTTGCCCAGACCAAATTAGCCCATTTCTGTATTTGGACTTTACAGATAACCTTTTAACAGACATAGTTTTTGAAGACTGTAGAAATTTAATTAGATTGAAAACACTTAGTTTACAAAAGAATCAGTTAAAAACACTTGAAAATATAATTCTTATGTCTATGGAGATGACATCCCTACAAAAACTAGACATTAGCCAGAATTCTCTAAGGTACAGTGATGCGGGAAGCCCATGCTCCTGGACCCAGAGTTTGTTAGTTTTAAATTTGTCTTCAAACATGCTTACGGACTCTGTCTTCAGGTGCTTACCTCCCAAGGTCAAGGTCCTTGACCTTCACAACAACAGGATAGTGAGCATCTCTAAAGATGTCACCCACCTGCAAGCTTTGCAGGAACTCAATGTCGCATCCAATTTTTTAACTGACCTTCCTGGATGTGGAGCCTTCAGTAGCCTTTCTGTGCTGGTCATCGACCATAACTCAGTTTCCCACCCCTCCTCTGATTTCTTCCAGAGCTGTCAGAATATCAGGTCCATAACAGCGGGGAACAACCCATTCCGATGCACATGTGAGCTGAGGGAGTTTGTCAAAAACATAGGTCAGGCATCAAGAGAAGTGGTGGAGGGCTGGCCTGACTCTTACAGGTGTGATTACCCAGACAGCATTAAGGGAACCCCACTGCAGGACTTCCACATGTCTCCACTGTCCTGCGATACAATTCTACTGACTGTCACCATTGGGGCCACTCTGCTGCTGCTGGCTGCCATTGGGGCTTCCCTCTGTCTCTACTTTGATCTGCCCTGGTATCTCAGGATGCTATGGCAGTGGACACAGACCAGGCACAGGGCCCGGAACATCCCCTTAGAGGAACTGCAGAGGAACCTCCAGTTCCATGCTTTTGTCTCATACAGTGGGCATGATTCTGCCTGGGTGAAGAATGAATTACTACCAAACCTAGAGAAAGATGACATTCGGGTTTGCCTCCATGAGAGAAACTTTGTCCCTGGCAAGAGCATTGTGGAGAACATCATACACTTCATTGAGAAGAGTTACAAGTCCATCTTTGTGCTGTCTCCCCACTTCATCCAGAGTGAGTGGTGCCATTATGAACTCTACTTTGCCCATCACAATCTCTTCCACGAAGGGTCTGATAACTTAATCCTGATCTTGCTGGAACCAATTCCACAGTACTCCATCCCTACCAATTACCACAAGCTCAAAACTCTCATGGCACGGAGGACCTATTTGGAATGGCCCACAGAAAAGAGCAAGCATGGACTCTTTTGGGCAAATCTAAGAGCATCCATTAATGTTAAGCTGGTCAACCAGGCAGAAGCAACATGTTACACACAGCAATAA

>TLR1EE

ATGACTAAAACTTACTCTATTGTCTTCCATTTAATCATCATCTTCATGCTAATAGTTAAGATCAGAACTCTACTATCTGATGGAAGTGATGTTTTAGCTGACAGATCAAACAGGACTCTCATTCATATTCCCAAGGATCTACCCCCCAGTACGACAATCCTAAATGTTTCACACAACTATATATCTGAGCTTTGGGCTTCTGACATCCTGTCACTCTCAAAGCTGAAGATCTTGATAATGTCTCATAACAGAATCCAGAATCTTGATATTAGTGTTTTCAGATTCAACCAGGAATTGGAATACTTGGATTTATCTCACAACAAATTGGAGACAATTTCTTGCCACTCCACTGCGAACCTTAAACATTTGGATCTCTCCTTCAATGCATTTGTTTTCCTACCCATATGCAAAGAGTTTGGTAACATGTCCCAGCTAGAATTTCTAGGGTTGAGTGCAAGCCAGTTACAAAAGTCTAGGTTGCTGTCAATTTCCCATCTGCACATCAGTAAGGTTTTACTGGTCTTAGGAGACTCCTATGGGGAAAAAGAAATCCCTGACAGCCTTCAAGACCTGAACACAGAAAGTCTTCACATTGTTTTTCCCCTGGGGAAGGAGTTCCATTTTAATTTGGATGTGTCAATCAGTCAGGCAGTAAGTCTGGAACTGTCTAATATCCAGTATGTGCTGGAAGATGGTGGTGCATGTTCTTTCCAAAATGCTCTGAGGAAACTTCAGAAGAATCCAAGGCTATCAAATCTTACTTTAAATAACATTGACACCACTTGGAATTCCTTCATGATGATTCTCCAGTTGGTTTGGCACACAGGCGTAGAATATTTCTCAATTAAAAATGTGAAACTACAAGGTTGGTTTCATCCCAGAGAGTTTAATTATTCTGACACTTCACTGAAATCCTTGACTATACATCAGGTTGTCAATAATGCATACAGTTTAGAACAAAATTCTATCTACAAAATCTTTGCAAATATGAACATCCAACATTTCACAGTGTCTGGTACACCTATGGTCCACATGCTTTGCCCATTACAAACTAGCCCATTTCTGTATTTGGATTTTTCTAATAATCTCTTGACAGACATGATTTTCAAAGATTGTGGAAATTTGACCAAATTAGAGACTCTTATTTTACAAATGAATCAACTACAAGAATTTACAAAGATAGTTTACATGACCAAGAAGATGAAGTCTCTACAACTACTGGATATTAGCCAGAATTCTCTAAGGATTGATGAAAATGAAGGAAATTGTTCTTGGACAGAAAGTTTATCAAGTTTAAATTTATCTTCAAATATACTTACTGAGTCTGTTTTCAGATGTCTACCTCCCAGGGTCAAGGTCCTTGATCTTCACAGTAACAGAATAAGGAGCATCCCAAGAGATGTCAACAATCTGGAAGCTTTGCAAGTACTCAATGTTGCTTCCAATTTTTTAACCAACCTTCCTGGATGTGGTGCCTTTAGCAGCCTTTCTGCACTGATCATTGACTATAACTCAATTTCCAGTCCATCAGTTGATTTCTTCCAGAGCTGCCAGAACATTAGGTCAGTAAAAGCAGGGAACAACCCATTCCAGTGTACATGTGAGCTCAGAGAGTTTGTCCAGAGAATGGGCCAAGTGTCAAGGGAAGTGGTAGAGGACTGGCCTGGTTCTTACCAGTGTGACTATCCAGAAAGCTTTAAGGGAACTGCACTAAAGGACTTCCACATGTCTCAGCTGTCCTGCAACACCACTCTGTTGATTGTCACCATTGTGGTCATTGTGCTGGTGTTGGGTAGTACCACGGTCATGCTCTGTATCTACTTTGATGTGCTCTGGTATCTGAGGATGATGTGCCATTGGACCCAGACCCGGCAAAGGGCTAGGAACACTCCCTTAGCTGAACTCCAGAGAAACCTCCAGTTCCATGCTTTCATTTCATATAGTGAACATGATTCCGCCTGGGTGAAGAGTGAATTACTACCAAACTTAGAAAAAGAAAATATACGAATTTGTCTTCATGAGAGAAACTTTGTCCCTGGGAAGAGCATCATAGAAAACATTATCAACTGCATTGAGAAAAGTTACAAGTCCATCTTTATTCTGTCTCCCAACTTTGTACAGAGTGAGTGGTGCCATTATGAGCTCTACTTTGCCCACCACAATCTCTTTCATAAAGGGTCTGATAACTTAATCTTGATCTTGCTAGAACCCATTCCACAGTATTCCATTCCTAACAGTTATCACAAGCTTAAAGCTCTCATGGCAAGAAGGACTTATTTGGAATGGCCCAAGGAGAAGAGAAAACATGGACTTTTTTGGGCTAGCTTAAGAGTATCCATTAATATTAAATTGACAGAGCAAGCAAAAGAAGTATGTCATACACAAATCCAAAATATTCTTACTACTTCTGCTTTTTGA

>TLR1BT

ATGACTAAAAAAAATTCTAGCATCTTCCATTTTGCCATCATCTTTATATTAATACTTGAGATCAGAACTCAATTATCTGATGAAAGTGAATTTTTAATTGACAGGTCAAAAAGAGGTCTCACCTATGTTCCCAAAAACTTATCCCTGGAAACAACCATCTTAGATATATCATACAACTATATTTCTGAGCTTCAGATGCCTGACATCCTCTCACTATCAAAGCTGAAGATTTTGATAATTTCTCATAATAGAATCCAGTATCTTGACTTGAGTGTTTTTAAATTCAACCAGGAACTGGAATACTTGGATTTGTCCCACAACAATTTGGAGAAGATTTCTTGCCACCCTACTCTGAACCTCAAGCACTTAGACCTCTCATTTAATCCATTTGATGCCCTGCCCATATGCCAAGAGTTTGGCAACATGTCTCAACTAGAATTTCTGGGGTTGAGTGCCACACAGTTACAGAAATCCAGTGTGCAGTCAATCACTCATTTGCACATCAGCAAGGTTTTATTGGTCTTAGGAGATACTTATGGGGAAAGAGAAGATGCCGAGAGCCTTCAAGACCTTAAGACACAGAGTCTGCACATTGTTTTCCCCACAGGAAAGGAATTCCATTTTATTTTGGACGTGTCAGTCGGCACCACAGTGAGTCTGGAACTGTCTAATATCAAATGTGTGCTTGATGATAATGGGTGTCCTTATTTCGAAAATGTTCTGTCAAAACTTCAAAAGAACTCAAGGTTATCAAATCTTACTTTAAACAACATTGAAATAACTTGGAATTCCTTCTTCACGATCCTCCAGTTGGTTTGGCGTACAAACATTGAGTACTTCTCCATTTCAAATGTGAAACTACAAGGTTACCTTGACTCTAGAGATTTTGATTATTCTGACACTTCACTGAAGGCCTTGTCTATACACAAAGTTGTCCATGATGTGTTCAGTCTTCCACAAGGTTATGTCTATAAAATATTGTCAAATATGAACATCCAGCATCTCACAGTGTCTGCTGCACACATGGTCCACATGGTCTGCCCATCCCAAATTAGCCCATTTCTGTATTTGAATTTTTCCAATAATCTCTTAACAGACACAGTTTTCATAAACTGTACAAATTTGGCTAATTTGAAGACACTTATCCTACAAAAGAATCAGTTAAAAGAACTTGTAAACATAGTTCATATGACCCAGGAAATGAAGTCTCTACAACAACTGGATGTTAGCCAGAATTCCCTGATGTATGATGAAAGTGAAGGAAATTGCCCTTGGGCCAGAAATTTATTAAGTTTAAATATGTCTTCAAATATACTTACTGACTCTGTTTTCAGATGTTTACCTCCTCAGATCAAGGTTCTTGATCTTCACAATAACAGAATAAGGAGCATCCCTAAAGATGTCACTGGTCTAGAAACTTTGCAAGAACTCAACCTTGCTTCCAATTCTTTAGCCCACCTTCCTGGATGTGGTATCTTTAGCAGCCTTTCCATACTGATCATTGACTATAACTCAATTTCCAATCCATCAGCTGATTTCTTCCAGAGCTGCCAGAAGATTAGGTCCCTCAAAGCGGGGAACAATCCATTCCAATGTTCCTGTGAGCTAAGAGACTTCATCCAAAGTATAGGCCAAGTATCAAGTGACGTGGTAGAGGGCTGGCCTGAGTCTTATAAGTGTGACTATCCGGAAAGCTACAAGGGAACCCCTCTAAAGGACTTCCAGGTATCTGAGCTATCCTGCAACACAGCTCTGCTGATCGTCACCATTGTGGTCCCTGGGCTGGTGCTGGCTGTTGCTGTGACTGTCCTCTGTATCTACCTGGATCTGCCCTGGTACCTCAGGATGGTGTGTCAGTGGACCCAGACCCGGCGCAGGGCCAGGAATGTACCCTTGGAAGAACTCCAAAGAACTCTCCAGTTCCATGCTTTTATTTCATATAGTGGGCACGATTCTGCCTGGGTGAAGAATGAATTAATACCTAACCTAGAAAAAGAAGATATAAGAATTTGTCTCCATGAGAGAAACTTTGTTGCTGGCAAGAGCATTGTGGAAAATATCATCAACTGCATTGAGAAAAGTTACAAATCCATCTTTGTCTTGTCTCCCAACTTTGTCCAGAGCGAATGGTGCCATTATGAACTCTACTTTGCCCACCACAATCTCTTCCATGAAGGATCTGATAACTTAATCCTGATCTTGCTGGATCCCATTCCACAGTATTCCATTCCTAGCAGCTACCACAAGCTAAGAGCTCTCATGGCACAGAGAACTTATTTGGAATGGCCCAAGGAGAAGAGTAAACACGGACTTTTTTGGGCTAACCTAAGAGCATCCATTAATATTAAACTGATGGAAAAAGCAGCAGAAATACATTAA

>TLR1SS

ATGACTAAAGAGAATCTTAGCATCTTCCATTTTGCCATCATCTTCATATTAATACTTGAGATCAGAATTCAATTATCTGAGGAAAGTGAAGTTTTAGTTGACAGATCAAAAACAGGTCTCACCCATGTTCCCAAAGACCTATCCTTGGAAACGACAATCTTAGATCTATCACAAAACTCTATCTCTGAGCTTCAGACTTCTGACATCCTCTCACTATCAAAGCTGAGGGTTTTCATAATTTCTCATAATAGAATACAGTATCTTGATGTCAGTGTTTTCAAATTCAACCAGGAACTGGAATACTTGGATTTGTCCCACAACAAGTTGGAGAAGATTTCGTGCCACCCTATGCTGAACCTCAAGCACTTGGACCTCTCATTTAATGCATTCGATGCCCTGCCCATATGCCAAGAGTTTGGCAGTATGTTTCAACTAGAATTTCTGGGGTTGAGTGCCACACAGTTACAAAAATCCAGTGTGTTGCCAATCGCTCATTTGCATATCGGTAAGGTTTTACTTGTCTTAGGAGACTCTTACGGGGAAAGAGAAGACCCTGAGAGCCTTCAAGACCTTAACACACAGAGTCTGCACATTGTTTATCCCCCAGGAAAGGAATTTCATTTTATGTTGGACGTGTCAGTCAGCACCGCAGTAAATCTGGAACTGTCCAATATCAGATGTGTGCTGGATGCTAACGGATGTCATCACTTCCAAAATGTTCTATTGAAACTTCAAAAGAACTCAAAGTTATCCAACCTGACTTTGAACAACATTGAAACCACTTGGAATTCTTTCATCACCACCCTCCAGTTTGTCTGGCATACAAGCATCGAGTATTTCTCCATTTCCAGTGTGAAACTACAAGGTCAGCTGGACTTCAGAGATTTTGATTATTCTGACACGTCACTGAAGGCCTTGTCTCTACACCAAGTTGTCAGTGAGGTGTTCAGTTTCCCACAAAGTTACATCTATAAAATCTTTTCAAATATGAACATTCAGTATCTCACAGTGTCTGCCACACACATGGTCCACATGGTTTGCCCATCCCAAATTAGCCCATTTCTATATTTGGATTTTTCCAATAATGCCTTAACAGACATGGTTTTTAAAAATTGTGCAAACTTGGCTAATTTGAACACACTCAGTTTACAAATGAATCAGTTAAAAGAACTTGTGAATGTCATCCATATGACCAAAGAAATGCAGTCTCTACAGCAATTGGATGTTAGCCAGAATACCCTGAGGTATGATGAAAATGAGGGAAGTTGCACTTGGACCGGAAGTTTATTAAGTTTAAATTTATCTTCAAATATACTCACTGACTCTGTTTTCAGATGTTTACCTCCCAGGATCAAGGTTCTTGATCTTCACAATAACAGAATAAGGAGCATCCCTAAGGATGTCGCCCATCTGGAAGCTCTGCAAGAACTCAATGTTGCTTCCAATTCTTTAGCTCACCTGCCTGGATGTGGTTCCTTTAGCAGCCTTTCCATTCTGATCATTGACTATAATTCAATTTCCAACCCATCAGCTGACTTCTTCCAGAGCTGCCAGAAGATTAGGTCCCTCAAAGCAGGGAACAATCCATTCCAATGTACATGTGAGCTAAGAGACTTCATCCAAAGTCTAGGTCAAGTATCGAGTGACGTGGTAGAGAGTTGGCCTGATTCTTACGAGTGTGAGTATCCAGAAAGCTATAAGGGGACTCTGCTCAAGGACTTCCGTGTATCTGAATTATCCTGCAACACAGCTCTGCTGATTGTCACCATCGGAGTCACTGGGCTGGCACTGGCTCTTACCATGACCGGCCTCTGTGTCTACTTTGATCTGCCCTGGTATCTCAGGATGCTGTGTCAGTGGACCCAGACTCGGCGCAGGGCTAGGAATGTACCCTTAGAAGAACTCCAAAGAACTCTCCAGTTCCATGCCTTCATTTCATATAGTGGGCATGATTCTGCTTGGGTAAAGAATGAATTACTACCAAATGTAGAAAAAGAAGGTATAAAGATTTGTCTCCATGAGAGAAACTTTGTTCCTGGCAAGAGCATCATGGAAAATATCATAAACTGCATTGAGAAAAGCTACAAGTCCATCTTTGTTTTGTCTCCCAACTTTGTCCAGAGCGAGTGGTGCCACTATGAACTCTACTTTGCCCACCACAACCTCTTCCATGAAGGGTCTGATAACTTAATCCTGATCTTGCTGGATTCCATTCCACAGTATTCCATCCCCAGCAGCTATCACAAACTCAAAGCTCTCATGGCACAGCGAACTTATTTGGAATGGCCCAAGGAGAAGAGCAAACATGGACTTTTTTGGGCTAATCTGAGAGCATCCATTAATATTAAATTGATGGAGAAAGCAGAAGAAATAAGTTACACACAGATCTAA

>TLR1CF

ATGATGAAAACTAATCCTAGCATCTTCCAATTTGCCATCATCTTCATATTAATACTTGAGATCAGAATACAATTGTCTGAAGAAAGTGATTTTCTAGTTAACAGATCAAAAGCAGGTCTCTTTCACATTCCCAAAGACCTATCCCTGAAAACAACAATCTTAGATATATCACAAAACTATATATCTGAGCTTCAGACTTCTGACATCCTATCACTATCAAAGCTGAGGATTTTGATTGTTTCTTATAATAGAATTCAATATCTTGATATCAGTGTTTTCAAATTCAACCAGGAATTGGAATACTTGGATCTGTCCCACAATGAGTTGGGGAGGATTTCTTGCCATCCTACCGTGAACCTCAAGCACTTAGACCTTTCATTTAATGCATTTGATGATCTACCCATATGCAAAGAGTTTGGCAACATGTCTCAACTAGAGTTTCTGGGGTTGAGTGCCACACAGTTACAGAAATCTAGCATGCTACCAATTGCTTCTTTGCATATCAGAAAGGTTTTACTGGTCTTAGGAGACACTTATGGGAAAAAAGAAGACCCTGAGAGCCTTCAAAAGCTTAACACAGAAAGTCTTCACATTGTTTTCCCTATAAGAAAGGAATTCAGTTTTACTCTGGATGTATCAGTCAGCACTGCAGTAAGTCTCGAATTGTCTAATATCAAATGTGTGCCAGATGGTCATGGATGGTCTTATTTCCAAAATGTTCTGTCAAAACTTCAAAAGAATTCAAGGTTATCAAGTCTTACTTTAAACAACATTGAAACAACTTGGAATTTTTTCATTATGCTCCTTCAGTTGGTTTGGCATACAAGCATAGAGTATTTCTCAATTTCAAATGTAAAACTACAAGGTTACCCTGACTTCAGAGATTTTGATTATTCTGACACTTCACTGAAGGCCTTATCTATACACCAAGTCGTTAGTAATGCATTCAATTTGCCACAAAGTTATATCTATAAAATCTTTTCAAATATGAACATCCAAAATTTCACAGTGTCTGGTACGCACATGGTCCACATGGTTTGCCCATCTCAAATTAGTCCATTTCTGCATTTGGATTTTTCTAATAATCTCTTAACAGACATTGTTTTTAAAAATTGTAGAAACTTGATTAAACTGGAGACACTTAGTTTACAAATGAATCAATTAAAAGAACTTGCAAGTATAGCTCAAATGACCAACGAGATGAAGTCTCTACAACAATTGGATATTAGCCAGAATTCTCTAAGGTATGATGAAAATGAAGGAAACTGCTCTTGGACTAGAAGTTTATTAAGTTTAAATATGTCTTCAAATATACTTACTGACTCTGTTTTCAGATGTTTACCTCCCAAGGTCAAGGTGCTTGATCTTCACGATAACAGAATAAGGAGCATTCCTAAACCAATCATGAAGCTAGAAGATTTGCAAGAACTCAATGTTGCTTCCAATTCTTTAGCCCACTTTCCTGACTGTGGTACTTTTAATAGGCTTTCTGTACTGATCATTGACTCTAATTCAATTTCCAATCCATCAGCTGATTTCCTCCAGAGCTGCCATAACATTAGGTCCATAAGCGCAGGGAATAATCCATTCCAGTGTACATGTGAGCTGAGAGAATTTGTCCAAAGTCTAGGCCAGGTAGCAAGCAAAGTAGTAGAGGGTTGGCCTGATTCTTATAAGTGTGACTCTCCAGAAAACTATAAGGGAACCCTACTGAAGGACTTTCACGTGTCTCCGTTATCCTGCAACACAACTCTGCTGCTTGTCACCATTGGGGTCGCTGTGCTAGTGTTCACTGTTACTGTGACTGCGCTCTGTATCTACTTTGATCTGCCCTGGTATCTTAGGATGGTGTTTCAGTGGACCCAGACCCGGCGCAGGGCAAGAAACACACCCTTAGAAAATCTCCAAAGAACCATCCAGTTCCATGCTTTTATTTCATATAGCGGGCATGATTCTGCCTGGGTGAAGAGTGAATTACTACCAAACCTAGAAAAAGAAGAACTAAGGATTTGTCTCCATGAGAGAAACTTTATTCCTGGCAAGAGCATTGTGGAAAATATCATAAACTGCATTGAGAAAAGTTACAAGTCCATCTTTGTTCTGTCTCCCAACTTTGTTCAGAGTGAGTGGTGCCATTATGAACTGTACTTTGCCCACCACAATCTCTTTCATGAAGGATCTAATAACTTAATCTTGATCTTGCTGGAACCTATTCCACAGTATTCCATTCCTAGCAGCTATCACAAGCTCAAAAATCTCATGGCACAAAGGACTTATTTGGAATGGCCCAAGGAGAAGAGCAAACATGGACTTTTTTGGGCTAACCTAAGAGCGTCTATTAATATTAAATTGAGGGAGCAAGCAAAAAAATAG

>TLR6HS

ATGACCAAAGACAAAGAACCTATTGTTAAAAGCTTCCATTTTGTTTGCCTTATGATCATAATAGTTGGAACCAGAATCCAGTTCTCCGACGGAAATGAATTTGCAGTAGACAAGTCAAAAAGAGGTCTTATTCATGTTCCAAAAGACCTACCGCTGAAAACCAAAGTCTTAGATATGTCTCAGAACTACATCGCTGAGCTTCAGGTCTCTGACATGAGCTTTCTATCAGAGTTGACAGTTTTGAGACTTTCCCATAACAGAATCCAGCTACTTGATTTAAGTGTTTTCAAGTTCAACCAGGATTTAGAATATTTGGATTTATCTCATAATCAGTTGCAAAAGATATCCTGCCATCCTATTGTGAGTTTCAGGCATTTAGATCTCTCATTCAATGATTTCAAGGCCCTGCCCATCTGTAAGGAATTTGGCAACTTATCACAACTGAATTTCTTGGGATTGAGTGCTATGAAGCTGCAAAAATTAGATTTGCTGCCAATTGCTCACTTGCATCTAAGTTATATCCTTCTGGATTTAAGAAATTATTATATAAAAGAAAATGAGACAGAAAGTCTACAAATTCTGAATGCAAAAACCCTTCACCTTGTTTTTCACCCAACTAGTTTATTCGCTATCCAAGTGAACATATCAGTTAATACTTTAGGGTGCTTACAACTGACTAATATTAAATTGAATGATGACAACTGTCAAGTTTTCATTAAATTTTTATCAGAACTCACCAGAGGTTCAACCTTACTGAATTTTACCCTCAACCACATAGAAACGACTTGGAAATGCCTGGTCAGAGTCTTTCAATTTCTTTGGCCCAAACCTGTGGAATATCTCAATATTTACAATTTAACAATAATTGAAAGCATTCGTGAAGAAGATTTTACTTATTCTAAAACGACATTGAAAGCATTGACAATAGAACATATCACGAACCAAGTTTTTCTGTTTTCACAGACAGCTTTGTACACCGTGTTTTCTGAGATGAACATTATGATGTTAACCATTTCAGATACACCTTTTATACACATGCTGTGTCCTCATGCACCAAGCACATTCAAGTTTTTGAACTTTACCCAGAACGTTTTCACAGATAGTATTTTTGAAAAATGTTCCACGTTAGTTAAATTGGAGACACTTATCTTACAAAAGAATGGATTAAAAGACCTTTTCAAAGTAGGTCTCATGACGAAGGATATGCCTTCTTTGGAAATACTGGATGTTAGCTGGAATTCTTTGGAATCTGGTAGACATAAAGAAAACTGCACTTGGGTTGAGAGTATAGTGGTGTTAAATTTGTCTTCAAATATGCTTACTGACTCTGTTTTCAGATGTTTACCTCCCAGGATCAAGGTACTTGATCTTCACAGCAATAAAATAAAGAGCGTTCCTAAACAAGTCGTAAAACTGGAAGCTTTGCAAGAACTCAATGTTGCTTTCAATTCTTTAACTGACCTTCCTGGATGTGGCAGCTTTAGCAGCCTTTCTGTATTGATCATTGATCACAATTCAGTTTCCCACCCATCGGCTGATTTCTTCCAGAGCTGCCAGAAGATGAGGTCAATAAAAGCAGGGGACAATCCATTCCAATGTACCTGTGAGCTAAGAGAATTTGTCAAAAATATAGACCAAGTATCAAGTGAAGTGTTAGAGGGCTGGCCTGATTCTTATAAGTGTGACTACCCAGAAAGTTATAGAGGAAGCCCACTAAAGGACTTTCACATGTCTGAATTATCCTGCAACATAACTCTGCTGATCGTCACCATCGGTGCCACCATGCTGGTGTTGGCTGTGACTGTGACCTCCCTCTGCATCTACTTGGATCTGCCCTGGTATCTCAGGATGGTGTGCCAGTGGACCCAGACTCGGCGCAGGGCCAGGAACATACCCTTAGAAGAACTCCAAAGAAACCTCCAGTTTCATGCTTTTATTTCATATAGTGAACATGATTCTGCCTGGGTGAAAAGTGAATTGGTACCTTACCTAGAAAAAGAAGATATACAGATTTGTCTTCATGAGAGAAACTTTGTCCCTGGCAAGAGCATTGTGGAAAATATCATCAACTGCATTGAGAAGAGTTACAAGTCCATCTTTGTTTTGTCTCCCAACTTTGTCCAGAGTGAGTGGTGCCATTACGAACTCTATTTTGCCCATCACAATCTCTTTCATGAAGGATCTAATAACTTAATCCTCATCTTACTGGAACCCATTCCACAGAACAGCATTCCCAACAAGTACCACAAGCTGAAGGCTCTCATGACGCAGCGGACTTATTTGCAGTGGCCCAAGGAGAAAAGCAAACGTGGGCTCTTTTGGGCTAACATTAGAGCCGCTTTTAATATGAAATTAACACTAGTCACTGAAAACAATGATGTGAAATCTTAA

>TLR6PT

ATGACCAAAGACAAAGAACCTATTGTTAAAAGCTTCCATTTTGTTTGCCTTATGATCATAATAGTTGGAACCAGAATCCACTTCTCCGACGGAAATGAATTTGCAGTAGACAAGTCAAAAAGAGGTCTTATTCATGTTCCAAAAGACCTACCGCTGAAAACCAAAGTCTTAGATATGTCTCAGAACTACATAGCTGAGCTTCAGGTCTCTGACATGAGCTTTCTATCAGAGTTGAAAGTTTTGAGACTTTCCCATAACAGAATCCAGCTACTTGATTTAAGTGTTTTCAAGTTCAACCAGGATTTAGAATATTTGGATTTATCTCATAATCAGTTGCAAAAGATATCCTGCCATCCTATTGTGAGTTTCAGGCATTTAGATCTCTCATTCAATGATTTCAAGGCCCTGCCCATCTGTAAGGAATTTGGCAACTTGTCACAACTGAATTTCTTGGGATTGAGTGCTATGAAGCTGCAAAAATTAGATTTGCTGCCAATTGCTCACTTGCATCTAAGTTATATCCTTCTGGATTTAAGAAATTATTATATAAAAGAAAATGAGACGGAAAGTCTACAAATTCTGAATGCAAAAACCCTTCACCTTGTTTTTCACCCAACTAGTTTATTCGCTATCCAAGTGAACATATCAGTTAATACTTTAGGGTGCTTACAACTGACTAATATTAAATTGAATGATGACAACTGTCAAGTTTTCATTAAATTTTTATCAGAACTCACCAGAGGTCCAACCTTACTGAATTTTACCCTCAACCACATAGAAACGACTTGGAAATGCTTGGTCAGAGTCTTTCAATTTCTTTGGCCCAAACCTGTGGAATATCTCAATATTTACAATTTAACAATAATTGAAAGCATTCATGAAGAAGAGTTTACTTATTCTAAAACGACATTGAAAGCATTGAAAATAGAACATATCACGAACAAAGTTTTTCTGTTTTCACAGACAGCGTTGTACACCGTGTTTTCTGAGATGAACATTATGATGTTAACCATTTCAGATACACCTTTTATACACATGCTGTGTCCTCATGCACCAAGCACATTCAAGTTTTTGAACTTTACCCAGAACGTTTTCACAGATAGTATTTTTGAAAAATGTTCCACGTTAGTTAAATTGGAGACACTTATCTTACAAAAGAATGGATTAAAAGACCTTTTCAAAGTAGGTCTTATGACTAAGGATATGCCTTCTTTGGAAATACTGGATGTTAGCTGGAATTCTTTGGAATCTGGTAGACATAAGGAAAACTGCACTTGGGTTGAGAGTATAGTGGTGTTAAATTTGTCTTCAAATATGCTTACTGACTCTGTTTTCAGATGTTTACCTCCCAGGATCAAGGTACTTGATCTTCACAGCAATAAAATAAAGAGCGTTCCTAAACAAGTCATAAAACTGAAAGCTTTGCAAGAACTCAATGTTGCTTTCAATTCTTTAACTGACCTTCCTGGATGTGGCAGCTTTAGCAGCCTTTCTGTATTGATCATTGATCACAATTCAGTTTCCCACCCATCAGCTGATTTCTTCCAGAGCTGCCAGAAGATGAGGTCAATAAAAGCAGGGGACAATCCATTCCAATGTACCTGTGAGCTAAGACAATTTGTCAAAAGTATAGACCAAGTATCAAGTGAAGTGTTAGAGGGCTGGCCAGATTCTTATAAGTGTGACTACCCAGAAAGTTATAGAGGAACCCCACTAAAGGACTTTCACATGTCTGAATTATCCTGCAACATAACTCTGCTGATCATCACCATCGGTGCCACCATGCTGGTGTTGGCTGTGACTGTGACCTCCCTCTGCATCTACTTGGATCTGCCCTGGTATCTCAGGATGGTGTGCCAGTGGACCCAGACCCGGCGCAGGGCCAGGAACATACCCTTAGAAGAACTCCAAAGAAATCTCCAGTTTCATGCTTTTATTTCATATAGTGAACATGATTCTGCCTGGGTGAAAACTGAATTGGTACCTTACCTAGAAAAAGAAGATATACAGATTTGTCTTCATGAAAGAAACTTTGTCCCTGGCAAGAGCATTGTGGAAAATATCATCAACTGCATTGAGAAGAGTTACAAGTCCATCTTTGTTTTGTCTCCCAACTTTGTCCAGAGTGAATGGTGCCATTACGAACTCTATTTTGCCCATCACAATCTCTTTCATGAAGGATCTAATAACTTAATCCTCATCTTACTGGAACCCATTCCACAGAACAGCATTCCCAACAAGTACCACAAGCTGAAGGCTCTCATGATGCAGCGGACTTATTTGCAGTGGCCCAAGGAGAAAAGCAAACGTGGGCTCTTTTGGGCTAACATTAGAGCCGCTTTTAATATGAAATTAACACTAGTCACTGAAAACAATGATGTGAAATCTTAA

>TLR6Ma

ATGACCAAAGACAAAGAACCTGTTGTTAAAAGCTTCCATTTTGTTTGCCTTATGATCATAATAGTTGGAACTAGAATCCAGTTCTCTGACGGAAGTGAATTTGCAGTAGACAAGTCAAAAAGAGGTCTTACTCATGTTCCAAAAGACCTACCGCCGAAAACCAAAGTCTTAGATATGTCTCACAACTACATAGCTGAGCTTCAGGTCTCTGACATAAGCTTTCTGTCAGAGTTGAAAGTTTTGAGACTTTCCCATAACAAAATCCAGCTACTTGATTTAAGTGTTTTCAAGTTCAACCAGGACTTGGAATATTTGGATTTATCTCATAATCAGTTGCAAAAGATATCCTGCCATCCTATTATGAGTTTCAGGCATTTAGATCTCTCATTCAATGACTTCGAGGCCCTGCCCATCTGTAAGCAATTTGGCAACTTGTCACAACTGAATTTCTTGGGGTTGAGTGCTATGAAGTTACAAAAATTAGATTTACTGCCAATTGCTCACTTGCATCTAAGTTACATCCTTCTGGATTTAAGAAATTATTATATAAAAGAAAATGAGACAGAAAGTCTACAAATTCTGAATGCAAAAACACTTCACCTTGTTTTTCACCCAACTAGTTTATTCTCTATCCAAGTGAACATATCAGTTAATACTTTAGGGTGCTTACAACTGACTAATATTAAATTGAATGATGACAACTGTCAAGTTTTCATTAAATTTTTATTAGAACTCACTAGAGGTCCAACCTTGCTGAATTTTACCCTCAATCACATAGAAACAACTTGGAAATGCCTGGTGAGAGTCTTTCAATTTCTTTGGCCCAAACCTGTGGAATATCTCAATATTTACAATTTAACAATAATTGAAAGCATTCATGAAGAAGATTTTACTTATTCTAAAACGACATTGAAAGCATTGAAAATAGAACATATCACGAACCAAGTTTTTATCTTTTCGCAGACAGCATTGTACACCGTGTTTTCTGAGATGAACATTATGATGTTAACCATTTCAGATACACCTTTTATACACATGCTGTGTCCTCGTGCACCAAGCACATTCAAGTTTTTGAACTTTACCCAGAATGTTTTCACAGATAGTATTTTTGAAAAATGTTCCACGTTAGTTAAATTGGAGACACTTATCTTACAAAAGAATGGATTAAAAGACCTTTTCAAAGTAGGTCTCATGACTAAGGATATGCCATCTTTGGAAATACTGGATGTTAGCTGGAATTCTTTGGAATCTGGTAGACATAGGGAAAACTGCACTTGGGTTGAGAGTATAGTGGTGTTAAATTTGTCTTCAAATATACTTACTGACTCCGTTTTCAGATGTTTACCTCCCAGGATCAAGGTACTTGATCTTCACAACAATAAAATAAAGAGCATTCCTAAACAAGTCGTAAAACTGGAAGCTTTGCAAGAACTCAATGTTGCTTTCAATTCTTTAACTGACCTTCCTGGATGTGGCAGCTTTAGCAGCCTTTCTGTATTGATCATTGATCACAATTCAGTTTCCCACCCATCAGCTGATTTCTTCCAGAGCTGCCAGAAGATGAGGTCAATAAAAGCAGGGAACAATCCATTCCAGTGTACCTGTGAGCTAAGAGAATTTGTCAAAAATATAGAGCAAGTATCAAGTGAAGTGGTAGAGGGCTGGCCTGATTCTTATAAGTGTGACTACCCAGAAAGTTATAGAGGAACCCCACTAAAGGACTTTCACATGTCTGAATTATCCTGCAACATAACTCTGCTGATCATCACCATCGGTGCCACCATGCTGGTGTTGGCTGTGACTGTGACCTTCCTCTGCATCTACTTGGATCTGCCCTGGTATCTCAGGATGGTGTGCCAGTGGACCCAGACCCGGCACAGGGCCAGGAATGTACCCTTAGAAGAACTCCAAAGAAATCTCCAGTTTCATGCATTTATTTCATATAGTGAACATGATTCTGCCTGGGTGAAAAATGAATTGGTACCTTACCTAGAGAAAGAAGGTATGCAGGTTTGCCTTCATGAGAGAAACTTTGTTCCTGGTAAGAGCATTGTGGAAAATATCATCAACTGCATTGAGAAGAGTTACAAGTCCATCTTTGTTTTGTCTCCCAACTTTGTCCAGAGTGAGTGGTGCCATTATGAACTCTACTTTGCCCATCACAATCTCTTTCATGAAGGATCTAATAACCTAATCCTCATCTTACTGGAACCCATTCCACAGAATAGCATTCCCAACAAGTACCACAAGCTGAGGGCTCTGATGACTCAGAGGACTTATTTGCAATGGCCCAAGGAGAAAAGCAAACGTGGGCTCTTTTGGGCTAACATTAGAGCCACTTTTAATGTGAAATTAACACTAGTCACTGAAAACAATGATGTGAAATCTTAA

>TLR6MM

ATGAGCCAAGACAGAAAACCCATCGTGGGGAGTTTCCACTTTGTTTGCGCCCTGGCCTTAATAGTCGGAAGCATGACCCCGTTCTCTAATGAACTTGAGTCTATGGTAGACTATTCAAACAGGAACCTTACTCATGTCCCCAAAGACCTGCCACCAAGAACAAAAGCCCTGAGTCTGTCTCAAAACTCTATATCTGAGCTTCGGATGCCTGATATCAGCTTTCTGTCAGAGCTGAGAGTTCTGAGACTCTCCCACAACAGGATACGGAGCCTTGATTTCCATGTATTCTTGTTCAATCAGGACTTAGAATACCTGGATGTCTCACACAATCGGTTGCAAAACATCTCTTGCTGCCCTATGGCGAGCCTGAGGCATCTAGACCTCTCATTCAATGACTTTGATGTACTGCCTGTGTGTAAGGAATTTGGCAACCTGACGAAGCTGACTTTCCTGGGATTAAGTGCTGCAAAGTTCCGACAACTGGATCTGCTCCCAGTTGCTCACTTGCATCTAAGCTGCATTCTTCTGGACTTAGTGAGTTATCATATAAAAGGCGGGGAAACAGAAAGTCTTCAGATTCCCAATACCACCGTTCTCCATTTGGTCTTTCATCCAAATAGCTTGTTCTCTGTTCAAGTGAACATGTCTGTAAACGCTTTAGGACATTTACAACTGAGTAATATTAAATTGAATGATGAAAACTGTCAAAGGTTAATGACATTTTTATCAGAACTCACCAGAGGTCCAACCTTATTGAATGTGACCCTCCAGCACATAGAAACAACCTGGAAGTGCTCGGTTAAACTTTTCCAATTCTTTTGGCCCCGACCGGTGGAGTACCTCAATATTTACAACTTAACGATAACTGAGAGAATCGACAGGGAAGAATTTACTTACTCGGAGACAGCACTGAAGTCACTGATGATAGAGCACGTCAAAAACCAAGTGTTCCTCTTTTCAAAGGAGGCGCTATACTCGGTGTTTGCTGAGATGAACATCAAGATGCTCTCTATCTCAGACACCCCTTTCATCCACATGGTGTGCCCGCCATCCCCAAGCTCATTTACATTTCTGAACTTTACCCAGAATGTTTTTACTGACAGTGTTTTTCAAGGCTGTTCCACCTTAAAGAGATTGCAGACACTTATCTTACAAAGGAATGGTTTGAAGAACTTTTTTAAAGTAGCTCTCATGACTAAGAATATGTCCTCTCTGGAAACTTTGGATGTTAGTTTGAATTCTTTGAACTCTCATGCATATGACAGGACATGCGCCTGGGCTGAGAGCATATTGGTGTTGAATTTGTCTTCGAATATGCTTACAGGCTCTGTCTTCAGATGCTTACCTCCCAAGGTCAAGGTCCTTGACCTTCACAACAACAGGATAATGAGCATCCCTAAAGATGTCACCCACCTGCAGGCTTTGCAGGAACTCAATGTAGCATCCAACTCCTTAACTGACCTTCCTGGGTGTGGGGCCTTCAGCAGCCTTTCTGTGCTGGTCATCGACCATAACTCAGTTTCCCATCCCTCTGAGGATTTCTTCCAGAGCTGTCAGAATATTAGATCCCTAACAGCGGGAAACAACCCATTCCAATGCACATGTGAGCTGAGGGACTTTGTCAAGAACATAGGCTGGGTAGCAAGAGAAGTGGTGGAGGGCTGGCCTGACTCTTACAGGTGTGACTACCCAGAAAGCTCTAAGGGAACTGCACTGAGGGACTTCCACATGTCTCCACTGTCCTGTGATACTGTTCTGCTGACTGTCACCATCGGGGCCACTATGCTGGTGCTGGCTGTCACTGGGGCTTTCCTCTGTCTCTACTTTGACCTGCCCTGGTATGTGAGGATGCTGTGTCAGTGGACACAGACCAGGCACAGGGCCAGGCACATCCCCTTAGAGGAACTCCAGAGAAACCTCCAGTTCCATGCTTTTGTCTCATACAGTGAGCATGATTCTGCCTGGGTGAAGAACGAATTACTACCCAACCTAGAGAAAGATGACATCCGGGTTTGCCTCCATGAGAGGAACTTTGTCCCTGGCAAGAGCATTGTGGAGAACATCATCAATTTCATTGAGAAGAGTTACAAGGCCATCTTTGTGCTGTCTCCCCACTTCATCCAGAGTGAGTGGTGCCATTATGAACTCTATTTTGCCCATCATAATCTCTTCCATGAAGGCTCTGATAACTTAATCCTCATCTTGCTGGAACCCATTCTACAGAACAACATTCCCAGTAGATACCACAAGCTGCGGGCTCTCATGGCACAGCGGACTTACTTGGAATGGCCTACTGAGAAGGGCAAACGTGGGCTGTTTTGGGCCAACCTTAGAGCTTCATTTATTATGAAGTTAGCCTTAGTCAATGAGGATGATGTGAAAACTTGA

>TLR6RN

ATGTCCCAAGACAGAGAACCCATCGTGGAGAGTTTCCATTTTGTTTGCACCCTGGCCTTAATAGTCGGAAGCATGACCCAGTTCTCTGATGAATTTGAGTCTGTAGTAGACTATTCAAACAAGAACCTTACTCATGTCCCAAAAGACCTGTCACCAAGCACAAAATCCTTGAGTCTGTCTCAAAACTCCATATCTGATCTTCAGATGTCTGATATCAGCTTTCTGTCAGAGCTGAGAGTTCTGAGACTCTCCCACAACAGAATACGGAGACTTGACTTTGGTGTGTTCTTGCTCAATCGGGACTTAGAATACCTGGATGTCTCTCACAATCAGTTACAAAACATCTCTTGCTGTCCTATGGTGAACTTGAAACATCTAGACCTCTCATTCAATGACTTTGAAGTGCTGCCCGTGTATAAGGAATTTGGCAACTTGATGAAGCTGAGTTTCTTGGGATTAAGTGCTGCAAAGTTCCGACAACTGGATCTGCTCCCAATTTCTCACTTGCACCTGAGCTGTGTTCTTCTGGACTTGGTGAATTATCAGATAAAAGATGGTGAAACAGAAAGTCTTCAGGTTCCAAATACCAACGTTCTCCATTTGGTCTTTCATCCGAATAGCCTGTTCTCTGTGCAAGTGAACATATCTGTAAATGCTTTAGGATGCTTACAACTGAGTAATATTAAATTGAATGATAAAAACTGTCAAAGCTTAATTATATTTTTATCAGAACTCACCAGAGGTCCAACCTTATTGAATCTGACCCTCCAGCACATAGAAACAAACTGGAAGTGCTTTGTTAGACTTTTACAATTCCTTTGGCCCAGACCTGTGGAGTATCTCAATATTTACAACTTAACGATAACTGAGAGCATAAGCAGGGAGACATTTATTTACGTGGAGACGGTGTTGAAGTCACTGAAGATAGAGCATGTCACAAACCAAGTGTTCCTCTTTGTGAAGGATGCACTATATTCTGTGTTTGCAGAAATGAACATCAGGATGCTCACACTGTCAGACACGCCATTCATCCACATGGTGTGCCCTGAGTTCCCAAGCACATTTGCATTTCTGAACTTTACCCAGAACGTTTTTACTGACAGCATTTTTCAAGGCTGTTCCACCTTGAAGAGACTGGAGACACTTATCTTGCAAAGGAATGGTTTAAAGAACCTTTTTAAGGTAGCTCTCATGACCAAGACTATGTCCTCTCTGGAAACATTGGATGTCAGTTTGAATTCTTTGAACTCTCATGTGTATGACAGAACATGTGCTTGGGCCGAGAGCATACGGGTGTTGAATTTGTCTTCGAATGTACTTTCGGACTCTGTCTTCAGGTGCTTACCTCCCAAGGTCAAGGTCCTTGACCTTCACAACAACAGGATAGTGAGCATCCCTAAAGATGTCACCCACCTGCAAGCTTTGCAGGAACTCAATGTCGCATCCAATTTTTTAACTGACCTTCCTGGATGTGGAGCCTTCAGTAGCCTTTCTGTGCTGGTCATCGACCATAACTCAGTTTCCCACCCCTCCTCTGATTTCTTCCAGAGCTGTCAGAATATCAGGTCCATAACAGCGGGGAACAACCCATTCCGATGCACATGTGAGCTGAGGGAGTTTGTCAAAAACATAGGTCAGGCATCAAGAGAAGTGGTGGAGGGCTGGCCTGACTCTTACAGATGTGATTACCCAGACAGCATTAAGGGAACCCCACTGCAGGACTTCCACATGTCTCCACTGTCCTGCGATACAATTCTACTGACTGTCACCATTGGGGCCACTCTGCTGCTACTGGCTGCCATTGGGGCTTCCCTCTGTCTCTACTTTGATCTGCCCTGGTATCTCAGGATGCTATGGCAGTGGACACAGACCAGGCACAGGGCCAGGAACATCCCCTTAGAGGAACTGCAGAGGAACCTCCAGTTCCATGCTTTTGTCTCATACAGTGAGCATGATTCTGCCTGGGTGAAGAATGAATTACTACCAAACCTAGAGAAAGATGACATTCGGGTTTGCCTCCATGAGAGAAACTTTGTCCCTGGCAAGAGCATTGTGGAGAACATCATACACTTCATTGAGAAGAGTTACAAGTCCATCTTTGTGCTGTCTCCCCACTTCATCCAGAGTGAGTGGTGCCATTATGAACTCTACTTTGCCCATCACAATCTCTTCCACGAAGGGTCTGATAACTTAATCCTGATCTTGCTGGAACCCATCCAACAGAACAACATTCCCAGTAGATACCACAAGCTGAGGGCTCTCATGGCACAGCGGACTTACTTGGAATGGCCTATTGAGAAGGGTAAACGTGGGCTGTTTTGGGCCAACCTTAGAGCTTCTTTTATTATGAAGCTAGCCTTAGTTAATGAGAATGATGTGAAAACTTGA

>TLR6EE

ATGACCAGAGACAAAGAATCTACTATGAGAAGTTTTGCTTATACTGTGATCGTAACAGTTGGGATCGTAATCCAGTCCTCTGATGAAAATGAATTTACAGTAGATTTGTCCAAAAGAGGCCTCACTCATATTCCAAGAGATTTGCCATCCCAAACCGAAGTCTTAGATATGTCTCAAAACAACGTATCTGAACTTCACCTTTCTGATATGAGCCTTATCTCAAGACTCAAAGTTTTAAGACTTTCCCATAACAGGATCCAGTGCCTTGATTTTAGTGTTTTCAGATTCAACCAGGATTTGGAATATTTGGATTTATCCCACAATCAGTTGAAAAAATTGTCCTGCCATGCCATCCAGAGTCTCCAGCATTTAGACCTCTCCTTCAATGACTTTGATAGTTTGCCCATTTGTAAGGAATTTGGCAACTTGACACAACTGAATTTCTTGGGATTAAGTACCTCAAAGTTGCAACAGTTAGACCTGCTACCAGTTTCTCACTTGCATGTAGACAGCATCCTTCTGGATTTACAAGGCTATTCTATGAAAGGAAACGAGACTGGGAGTCTTCAAATTTTCAATACAAAAAAACTTCACTTTGTTTTTCATCCAAATGATTTGTTCTCTGTGCTAGTGAACATATCTGTTAACATGATAGAGTGCTTACAGCTGACTAATATCAAACTGAATGATGACAACTGTCAAGTTTTAATAAATTTCTTATCAGAACCACTTATCAGAGGCCCAAACTTACTGAATTTGACCCTAGATCATGTGGAAACAACTTGGAAATGTTTAGTTAGAGTTTTTCAGTCCCTTTGGTCCAAGCCTATAGAATACCTGAATATTTACAATTTGACAGTAGTTGAAAGAATTGATGAAGAAGAGTTCACTTACTCCAAAACATCATTGAAGGCACTGAAAATAGAGCATATTACAAATAGAGTTTTTCTTTTTTCACAGACTGCATTGTACACAGTGTTTTCTGAGATGAACATTATGATGTTAACCATATCAGAGGCACCTTTAATACACATGCTTTGTCCTCAGGCACCAAGCATGTTCAAGTTTTTGAACTTTACCCACAATGTTTTTACAGATAGTATTTTTCAAAACTGTTCCACACTAGGTAGATTAGAAACACTTATCTTACAGAAGAATGAATTGAAAGAACTTTTTAAAGTAGGTCTAATGACTAAGGATATGCAGTCTTTGGAAATACTGGATGTAAGCTGGAATTCTCTGAACTATGATAGATATGATGGAATTTGTACGTGGGCTCAGAGTATAGTGATGTTAAATTTATCTTCAAATATACTTACTGAGTCTGTTTTCAGATGTCTACCTCCCAGGGTTAAGGTCCTTGATCTTCACAGTAACAGAATAAGGAGCATCCCAAGAGATGTCAACAATCTGGAAGCTTTGCAAGTACTCAATGTTGCTTCCAATTTTTTAACCAACCTTCCTGGATGTGGTGCCTTTAGCAGCCTTTCTGCACTGATCATTGACTATAACTCAATTTCCAGTCCATCAGTTGATTTCTTCCAGAGCTGCCAGAACATTAGGTCAGTAAAAGCAGGGAACAACCCATTCCAGTGTACATGTGAGCTCAGAGAGTTTGTCCAGAGAATGGGCCAAGTGTCAAGGGAAGTGGTAGAGAACTGGCCTGGTTCTTACCAGTGTGACTATCCAGAAAGCTTTAAGGGAACTGCACTAAAGGACTTCCACATGTCTCAGCTGTCCTGCAACACCACTCTGTTGATTGTCACCATTGTGGTCATTGTGCTGGTGTTGGGTAGTACCACGGTCATGCTCTGTATCTACTTTGATGTGCTCTGGTATCTGAGGATGATGTGCCATTGGACCCAGACCCGGCAAAGGGCTAGGAACACTCCCTTAGCTGAACTCCAGAGAAACCTCCAGTTCCATGCTTTCATTTCATATAGTGAACATGATTCCGCCTGGGTGAAGAATGAGCTGGTACCCTGCCTAGAAAAAGAAAATATACGAATTTGTCTTCATGAGAGAAACTTTGTCCCTGGGAAGAGCATCATAGAAAACATTATCAACTGCATTGAGAAAAGTTACAAGTCCATCTTTGTTCTGTCTCCCAACTTTGTACAGAGTGAGTGGTGCCATTATGAGCTCTACTTTGCCCACCACAATCTCTTTCATGAAGGGTCTGATAACTTAATCTTGATCTTGCTGGAACCCATTCCACAGAACAACATTCCTAGTAAGTATCACAAGCTGAAGGCTCTCATGACACAACGAACTTATTTGGAATGGCCTAAGGAGAAGAGCAAACATGGACTTTTCTGGGCTAACATCACAGCTGCTTTTCATATGAAATTAACACTAGTGAATGAAAATGATGCAGAAACTTAG

>TLR6BT

ATGATCAAAGACAAAGAATCTCCCATCAGAAGCTGTCATTTTGTTTACATTGTGGCCTTAGTATTTGGAACCATAATCCAGTTCTCTGATGAAAGTGAATTTGTGGTAGACATGTCAAAAACAAGCCTTATTCATGTTCCCAAAGACCTGCCACCAAAAACCAAAGTCTTAGACTTGTCTCAAAACAACATATCTGAGCTTCACCTGTCTGATATCAGCTTTCTCTCAGGGCTGAGAGTTCTGAGACTTTCCCATAATAGAATCCAGGGCCTTGATATTAGTATTTTCAAGTTCAACCATGATTTGGAATATTTGGATTTATCTCATAATCAGTTGCAGAAGATATCCTGCCATCCAATCACCACGACTCTCAAGCATTTAGACCTCTCATTCAATGACTTCGATGCCCTGCCCATCTGTAAGGAATTTGGCAACTTGACCCAACTGAATTTCTTAGGATTAAGTGCTACAAAGTTACAACAATTAGATTTACTACCCATTGCTCACTTGCACCTAAGTTGTATCCTTCTGGATTTGGAAGACTATATGAAAGAAAATAAGAAAGAAAGTCTTCAAATTCTGAATACAAAGAAACTTCACCTTGTTTTTCACCCAAATAGCTTTTTCTCTGTCCAAGTGGACATATCAGCGAATAGTTTAGGGTGCTTACAACTGACTAATATTAAATTGAATGATTACAATTGTCAAGTTTTACTTAAATTTTTATCAGGACTCACTGGAGGACCAACCTTACTAAATTTTACCCTCAACCACATGGAAACAACTTGGAAATGTTTGGTTAAAGTTTTTCAGTTCCTTTGGCCCAAACCTATAGAATATCTCAATATTTACAATTTAACAATAGTTGAAAGCATTGATGAAGAAGTTTTTACTTATTATAAAACGACATTGAAGGCACTGAAAATAGAACATATTACAAACAAAGTTTTTATTTTTTCACAGACAGCATTATACACAGTGTTTTCTGAGATGAACATTCTGATGTTAACCATATCAGACACACGCTTTATACACATGCTTTGTCCTCAGGAACCAAGCACATTTAAGTTTTTGAACTTTACCCAGAATAGTTTCACAGATAGTGTCTTTCAAAATTGTGACACTTTAGCTAGATTGGAGACACTTATCTTACAAAAGAATGAATTAAAAGACCTTTTCAAAACAAGTCTCATGACTAAGGATATGCTTTCTTTGGAAACACTGGATGTTAGCTGGAATTCTTTGGAATATGACAGAAGTAATGGAAATTGCTCTTGGGTTGGGAGTATAGTGGTGTTAAATTTATCTTCAAATGCACTCACTGACTCTGTTTTCAGATGTTTACCTCCTCGGATCAAGGTTCTTGATCTTCACAATAACAGAATAAGGAGCATCCCTAAAGATGTCACTGGTCTAGAAACTTTGCAAGAACTCAACCTTGCTTCCAATTCTTTAGCCCACCTTCCTGGATGTGGTATCTTTAGCAGCCTTTCCATACTGATCATTGACTATAATTCAATTTCCAATCCATCAGCTGATTTCTTCCAGAGCTGCCAGAAGATTAGGTCCCTCAAAGTGGGGAACAATCCATTCCAATGTTCCTGTGAGCTAAGAGACTTTATCCAAAGTGTAGGCCAAGTATCCAGTGACGTGGTAGAGGGCTGGCCTGAGTCTTATAAGTGTGACTATCCGGAAAGCTACAAGGGAACCCCTCTAAAGGACTTCCAGGTATCTGAGCTATCCTGCAACACAGCTCTGCTGATCGTCACCATTGTGGTCCCTGGGCTGGTGCTGGCTGTTGCTGTGACTGTCCTCTGTATCTACCTGGATCTGCCCTGGTACCTCAGGATGGTGTGTCAGTGGACCCAGACCCGGCGCAGGGCCAGGAATGTACCCTTGGAAGAACTCCAAAGAACTCTCCAGTTCCATGCTTTTATTTCATACAGTGAACATGACTCTGCCTGGGTGAAGAATGAATTAATACCTAACCTAGAAAAAGAAGATATAAGAATTTGTCTCCACGAGAGAAACTTTGTTGCCGGCAAGAGCATTGTGGAAAATATCATCAACTGCATTGAGAAAAGTTACAAATCCATCTTTGTCTTGTCTCCCAACTTTGTCCAGAGCGAATGGTGCCATTATGAACTCTACTTTGCCCACCACAATCTCTTCCATGAAGGATCTAATAACTTAATCCTGATCTTGCTGGAACCCATTCCACAGAACACCATTCCTGATAGATATCACAAGCTAAGAGCTCTCATGGCACAGAGAACTTATTTGGAATGGCCCAAGGAGAAGAACAAGCATGGACTCTTTTGGGCTAATATTAGAGCTGCTTTTAATATTAAATTAAGACTAGTCACTGAAAATGATGATGTGAAAGGTTAA

>TLR6SS

ATGAGCAAAGACAAAGAACCTACTGTCATAAGCCTTCATTCTGTGTATGTCATGACCTTAGTATGGGGAACCCTAATCCAGTTCTCTGAAGAAAGTGAATTTGTGGTAGACAAGTCAAAAATAGGCCTTACTCGTGTTCCAAAAGACCTGCCACCCCAAACCAAAGTCTTAGATGTGTCTCAAAACTTCATAACTGAGCTTCACCTCTCTGACATCAGCTTTCTCTCGCAGCTGACGGTTTTGAGACTTTCCCAGAATAGGATGCAGTGCCTTGATATCAGTGTTTTCAAGTTCAATCAGGATTTGGAATATTTGGATTTATCTCACAATCAGTTGCAGACAATCTTGTGCCATCCCATCACCAGCCTCAAGCATTTGGACCTCTCATTCAATGACTTTGAAGCCCTGCCCATATGTAAGGAGTTTGGCAACTTGACACAACTGAATTTCTTGGGATTAAGTGCTACAAAGTTACAGCAATTAGATCTACTACCAATTGCTCACTTGCATCTAAGTTGCATCCTTCTGGATTTGGAACGTTATTACATGAAAGAAAATGAGAAAGAAAGTCTTCAAATTCTGAACACAAAGAAACTTCACCTGGTCTTTCATCCAAATAGCTTCTTCTCTGTCCAAGTAAACATATCGGTTAAGAGTGTAGGGTGTTTACAACTGGCTAATATTAAACTGAGTGATGACAACTGTCAGGTTTTCATTACATTTTTATTGGAACTCACTCAAGGGCCAACCTTACTAAATTTTACGCTCAACCATGTGGAAACAACTTGGAAATGTTTGGTTGGAATTTTTCAATTCCTTTGGCCCAAACCTGTAGAATATCTCAGTATTTACAATTTAACAATAGTTGAAAGCATTGATGAAGAAGATTTTATTTATTATGAAACAACATTGAAAGGAGTGAAAATAGAACATATTACAAAGAGAGTTTTTATTTTTTCACAGACAGCATTATACAGAGTGTTTTCCGATATGAATATCAGGATGTTAACCATAGCAGACACACATTTTATACACATGCTTTGTCCTCAGGTACCAAGCACATTTAACTTTTTGAACTTTACCCAGAATGTTTTTACAGATAGTGTTTTTCAAAATTGCAAAACTTTAGCTAGATTAGAGACACTCATCTTACAAAAGAATAAATTAGAAGACCTTTTCAAAATAAGCCTCATGACTAAGGATATGCTATCTTTGGAAATACTGGATGTTAGCTCGAATTCTTTGGAGTATGATAGACATGGTGAAAATTGCACTTGGGTTGGGAGTATAGTGGTGTTAAATTTATCTTCAAATATACTCACTGACTCTGTTTTCAGATGTTTACCTCCCAGGATCAAGGTTCTTGATCTTCACAGTAACAGAATAAGGAGCATCCCTAAGGATGTCGCCCATCTGGAAGCTCTGCAAGAACTCAATGTTGCTTCCAATTCTTTAGCTCACCTGCCTGGATGTGGTTCCTTTAGCAGCCTTTCCATTCTGACCATTGACTATAATTCAATTTCCAACCCATCAGCTGACTTCTTCCAGAGCTGCCAGAAGATTAGGTCCCTCAAAGCAGGGAACAATCCATTCCAATGTACATGTGAGCTAAGAGACTTCATCCAAAGTCTAGGTCAAGTATCGAGTGACGTGGTAGAGAGTTGGCCTGATTCTTACGAGTGTGAGTATCCAGAAAGTTATAAGGGGACTCTGCTCAAGGACTTCCGTGTATCTGAATTATCCTGCAACACAGCTCTGCTGATTGTCACCATCGGAGTCACTGGGCTGGCATTGGCTCTTACCATGACCGGCCTCTGTGTCTACTTTGATCTGCCCTGGTATCTCAGGATGCTGTGTCAGTGGACCCAGACTCGGCGCAGGGCTAGGAATGTACCCTTAGAAGAACTCCAAAGAACTCTCCAGTTCCATGCCTTCATTTCATATAGTGAACACGATTCTGCCTGGGTAAAAAATGAACTGGTACCTTGTCTAGAAAAAGAAGGTATAAAGATTTGTCTCCATGAGAGAAACTTTGTTCCTGGCAAGAGCATCATGGAAAATATCATAAACTGCATTGAGAAAAGCTACAAGTCCATCTTTGTTTTGTCTCCCAACTTTGTCCAGAGCGAGTGGTGCCACTATGAACTCTACTTTGCCCACCACAACCTCTTCCATGAAGGGTCTGATAACTTAATCCTGATCTTGCTGGATCCCATTCCACAGAACAGCATTCCTGGCAAGTATCACAAACTCAAAGCTCTCATGGCACAGCGAACTTATTTGGAATGGCCCAAGGAGAAGAGCAAACATGGACTTTTTTGGGCTAATATTAGAGCTGCTTTCAATATTAAATTAAAACTAGTCGCTGAAGAGGATGATGTGAAAACTTAA

>TLR6CF

ATGATCAAAGACAAAGACTCTATCACTGGAAGCTTCCATTTTGTGTACATTGTGACCTTAATAGTTGGAACCATAATCCAATTCTCTGATGAAAGTGAATTTACAGTAGACATGTCAAATATGAACCTTACTCATGTCCCAGAAGACCTGCCACCAAAAACCAAAATCTTAGATATGTCTCAGAACAATATATCTGAGCTTCACCTCTCTGACATGAGCTATCTCTCAGGACTAAAAATATTGAGAATTTCTCATAATAGAATCTGGTGGCTTGATTTTAGCATTTTCAAGTTCAACCAGGATTTGGAATATTTGGATTTATCTTACAATCAGTTACGGAACATGTCCTGCCATCTTATCAGGAGTCTCAAGCATTTAGACCTCTCATTCAATGACTTTCATGTCCTGCCCATCTGTAAGGAATTTGGCAACTTGACACAACTACAATTCTTAGGTTTAAGTGCTACAAAGTTACGGCAATTAGATCTGCTACCAATTGCTCATTTGCATCTAAGTTATATCCTTCTGGATTTACAAGGTTATTATGCAAAAGAAAGTGAAAAAGGAAGTCTTCAAATTCTAGATACAAAAACACTTCATCTTGTTTTTCATCCAAATCAGTTATTCTCTGTACAAGCAAACATGTTAGTTAATAATTTAGGGTGCTTACAACTGACTAATATTAAGTTGAATAATGACAACTGTCAAGTTTTAATTCAATTTTTATCAGAACTCACCAGAGGTCCAACTTTACTGAATTTTACTCTCCAACATGTGAAAACAACTTGGAAATGCTTGGTTAGAATTTTTAAATTTCTTTGGCCCAAACCTGTACAATATCTCAATATTTATAATTTAACAATAGTTGAAAGCATTAATAAGGAATATATTCATTATCCTAAAACAGCACTGAAAGCATTGACAATAGAGCATGTTAAAAATGAAGTTTTTCTTTTTTCACAGACAGCGTTATACACAATTTTTTCTGAGATGAACATTATGATGTTAACCATATCAGACACACCTTTTATACACATGCTTTGTCCTCCACCATCAAACACATTTAAGTTTTTGAACTTTACCCAGAATGTTTTCACAGATAGTGTTTTTCAAAGTTGTTCCCACCTAGTTAGATTGGAAACACTTATCTTACGAAAGAATAAATTGAAAGACCTTTACAAAGTAGGTCTCATGACGAAGCATATGACATCTTTGGAAATATTGGATGTTAGTGTGAATTCTTTGGAATATGATAGATATGATGGAAATTGCACTTGGGTTGGGAGTATAGTGGTGTTAAATTTGTCTTCAAATATACTTACTGACTCTGTTTTCAGATGTTTACCTCCCAAGGTCAAGGTGCTTGATCTTCACGATAACAGAATAAGGAGCATTCCTAAACCAATCATGAAGCTAGAAGATTTGCAAGAACTCAATGTTGCTTCCAATTCTTTAGCCCACTTTCCTGACTGTGGTACTTTTAATAGGCTTTCTGTACTGATCATTGACTCTAATTCAATTTCCAATCCATCAGCTGATTTCCTCCAGAGCTGCCATAACATTAGGTCCATGAGCGCAGGGAATAATCCATTCCAATGTACATGTGAGCTGAGAGAATTTGTCCAAAGTCTAGGCCAGGTAGCAAGCAAAGTAGTAGAGGGTTGGCCTGATTCTTATAAGTGTGACTCTCCAGAAAACTATAAGGGAACCCTACTGAAGGACTTTCACGTGTCTCCGTTATCCTGCAACACAACTCTGCTGCTTGTCACCATTGGGGTCGCTGTGCTAGTGTTCACTGTTACTGTGACTGCGCTCTGTATCTACTTTGATCTGCCCTGGTATCTTAGGATGGTGTTTCAGTGGACCCAGACCCGGCGCAGGGCAAGAAACACACCCTTAGAAGAACTCCAAAGAACCATCCAGTTCCATGCTTTTATTTCATACAGTGAACATGATTCTGCCTGGGTGAAGAATGAACTGGTACCCTGCCTAGAAAAAGAAGAACTAAGGATTTGTCTCCATGAGAGAAACTTTATTCCTGGCAAGAGCATTGTGGAAAATATCATAAACTGCATTGAGAAAAGTTACAAGTCCATCTTTGTTCTGTCTCCCAACTTTGTTCAGAGTGAGTGGTGCCATTATGAACTGTACTTTGCCCACCACAATCTCTTTCATGAAGGATCTAATAACTTAATCTTGATCTTGCTGGAACCCATTCCACAGAACTGCATTCCCAGCAAGTATCACAAGCTGAGGGCTCTCATGACGCAGCGGACTTACTTGGAATGGCCCAAGGAGAAGAGCAAACATGGACTTTTTTGGGCTAATATTAGAGCTGCTTTTAATATGAAGTTAACACTAATTGCTGAAAACAATAACGCAGAAGCTTCTTAA

>TLR10HS

ATGAGACTCATCAGAAACATTTACATATTTTGTAGTATTGTTATGACAGCAGAGGGTGATGCTCCAGAGCTGCCAGAAGAAAGGGAACTGATGACCAACTGCTCCAACATGTCTCTAAGAAAGGTTCCCGCAGACTTGACCCCAGCCACAACGACACTGGATTTATCCTATAACCTCCTTTTTCAACTCCAGAGTTCAGATTTTCATTCTGTCTCCAAACTGAGAGTTTTGATTCTATGCCATAACAGAATTCAACAGCTGGATCTCAAAACCTTTGAATTCAACAAGGAGTTAAGATATTTAGATTTGTCTAATAACAGACTGAAGAGTGTAACTTGGTATTTACTGGCAGGTCTCAGGTATTTAGATCTTTCTTTTAATGACTTTGACACCATGCCTATCTGTGAGGAAGCTGGCAACATGTCACACCTGGAAATCCTAGGTTTGAGTGGGGCAAAAATACAAAAATCAGATTTCCAGAAAATTGCTCATCTGCATCTAAATACTGTCTTCTTAGGATTCAGAACTCTTCCTCATTATGAAGAAGGTAGCCTGCCCATCTTAAACACAACAAAACTGCACATTGTTTTACCAATGGACACAAATTTCTGGGTTCTTTTGCGTGATGGAATCAAGACTTCAAAAATATTAGAAATGACAAATATAGATGGCAAAAGCCAATTTGTAAGTTATGAAATGCAACGAAATCTTAGTTTAGAAAATGCTAAGACATCGGTTCTATTGCTTAATAAAGTTGATTTACTCTGGGACGACCTTTTCCTTATCTTACAATTTGTTTGGCATACATCAGTGGAACACTTTCAGATCCGAAATGTGACTTTTGGTGGTAAGGCTTATCTTGACCACAATTCATTTGACTACTCAAATACTGTAATGAGAACTATAAAATTGGAGCATGTACATTTCAGAGTGTTTTACATTCAACAGGATAAAATCTATTTGCTTTTGACCAAAATGGACATAGAAAACCTGACAATATCAAATGCACAAATGCCACACATGCTTTTCCCGAATTATCCTACGAAATTCCAATATTTAAATTTTGCCAATAATATCTTAACAGACGAGTTGTTTAAAAGAACTATCCAACTGCCTCACTTGAAAACTCTCATTTTGAATGGCAATAAACTGGAGACACTTTCTTTAGTAAGTTGCTTTGCTAACAACACACCCTTGGAACACTTGGATCTGAGTCAAAATCTATTACAACATAAAAATGATGAAAATTGCTCATGGCCAGAAACTGTGGTCAATATGAATCTGTCATACAATAAATTGTCTGATTCTGTCTTCAGGTGCTTGCCCAAAAGTATTCAAATACTTGACCTAAATAATAACCAAATCCAAACTGTACCTAAAGAGACTATTCATCTGATGGCCTTACGAGAACTAAATATTGCATTTAATTTTCTAACTGATCTCCCTGGATGCAGTCATTTCAGTAGACTTTCAGTTCTGAACATTGAAATGAACTTCATTCTCAGCCCATCTCTGGATTTTGTTCAGAGCTGCCAGGAAGTTAAAACTCTAAATGCGGGAAGAAATCCATTCCGGTGTACCTGTGAATTAAAAAATTTCATTCAGCTTGAAACATATTCAGAGGTCATGATGGTTGGATGGTCAGATTCATACACCTGTGAATACCCTTTAAACCTAAGGGGAACTAGGTTAAAAGACGTTCATCTCCACGAATTATCTTGCAACACAGCTCTGTTGATTGTCACCATTGTGGTTATTATGCTAGTTCTGGGGTTGGCTGTGGCCTTCTGCTGTCTCCACTTTGATCTGCCCTGGTATCTCAGGATGCTAGGTCAATGCACACAAACATGGCACAGGGTTAGGAAAACAACCCAAGAACAACTCAAGAGAAATGTCCGATTCCACGCATTTATTTCATACAGTGAACATGATTCTCTGTGGGTGAAGAATGAATTGATCCCCAATCTAGAGAAGGAAGATGGTTCTATCTTGATTTGCCTTTATGAAAGCTACTTTGACCCTGGCAAAAGCATTAGTGAAAATATTGTAAGCTTCATTGAGAAAAGCTATAAGTCCATCTTTGTTTTGTCTCCCAACTTTGTCCAGAATGAGTGGTGCCATTATGAATTCTACTTTGCCCACCACAATCTCTTCCATGAAAATTCTGATCATATAATTCTTATCTTACTGGAACCCATTCCATTCTATTGCATTCCCACCAGGTATCATAAACTGAAAGCTCTCCTGGAAAAAAAAGCATACTTGGAATGGCCCAAGGATAGGCGTAAATGTGGGCTTTTCTGGGCAAACCTTCGAGCTGCTATTAATGTTAATGTATTAGCCACCAGAGAAATGTATGAACTGCAGACATTCACAGAGTTAAATGAAGAGTCTCGAGGTTCTACAATCTCTCTGATGAGAACAGATTGTCTATAA

>TLR10PT

ATGAGACTCATCAGAAACATTTACATATTTTGTAGTATTGTTATGACAGTAGAGGGTGATGCTCCAGAGCTGCCAGAAGAAAGGGAACTGATGACCAACTGCTCCAACATGTCTCTAAGAAAGGTTCCCGCAGACTTGACCCCAGCCACAACAACACTGGATTTATCCTATAACCTCCTTTTTCAACTCCAGAGTTCAGATTTTCATTCTGTCTCCAAACTGAGAGTTTTGATTCTATGCCATAACAGAATTCAACAGCTGGATCTCAAAACCTTTGAATTCAACAAGGAGTTAAGATATTTAGATTTGTCTAATAACAGACTGAAGAGTGTAACTTGGTATTTACTGGCAGGTCTCAGGTATTTAGATCTTTCTTTTAATGACTTTGACACCATGCCTATCTGTGAGGAAGCTGGCAACATGTCACACCTGGAAATCCTAGGTTTGAGTGGGGCAAAAATACAAAAATCAGATTTCCAGAAAATTGCTCATCTGCATCTAAATACTGTCTTCTTAGGATTCAGAACTCTTTCTCATTATGAAGAAGGTAGCCTGCCCATCTTAAACACAACAAAACTGCACATTGTTTTACCAATGGACACAAATTTCTGGGTTCTTTTGCGTGATGGAATCAAGACTTCAAAAATATTAGAAATGACAAATATAGATGGCAAAAGCCAATTTGTAAGTTATGAAATGCAACGAAATCTTAGTTTAGAAAATGCTAAGACATCGATTCTATTACTTAATAAAGTTGATTTACTCTGGGACGACCTTTTCCTTATCTTACAATTTGTTTGGCATACATCAGTGGAACACTTTCAGATCCGAAATGTGACTTTTGGTGGTAAGGCTTATCTTGACCACAATTCATTTGACTACTCAAATACTGTAATGAGAACTATAAAATTGGAGCATGTACATTTCAGAGTGTTTTACATTCAACAGGATAAAATCTATTTGCTTTTGACCAAAATGGACATAGAAAACCTGACAATATCAAATGCACAAATGCCACACATGCTTTTCCCTAATTATCCTACGAAATTCCAATATTTAAATTTTGCCAATAATATCTTAACAGACGAGTTGTTTAAAAGAACTATCCAACTGCCTCACTTGAAAACTCTCATTTTGAATGGCAATAAACTGGAGACACTTTCTTTAGTGAGTTGCTTTGCTAACAACACACCCTTGGAACACTTGGATCTGAGTCAAAATCTATTACAACATAAAAATGATGAAAATTGCTCATGGCCAGAAACTGTGGTCAATATGAATCTGTCATACAATAAATTGTCTGATTCTGTCTTCAGGTGCTTGCCCAAAAGTATTCAAATACTTGACCTAAATAATAACAAAATCCAAACTGTACCTAAAGAGACTATTCATCTGATGGCCTTACGAGAACTAAATATTGCATTTAACTTTCTAACTGATCTCCCTGGGTGCAGTCATTTCAGTAGACTTTCAATTCTGAACATTGAAATGAACTTCATTCTCAGCCCATCTCTGGATTTTGTTCAGAGCTGCCAAGAAGTTAAGACTCTAAATGCGGGAAGAAATCCATTCCGGTGTACCTGTGAATTAAAAAATTTCATTCAGCTTGAAACATATTCAGAGGTCATGATGGTTGGATGGTCAGATTCATACACCTGTGAATACCCTTTAAACCTAAGGGGAACTCGGTTAAAAGACGTTCATCTCCACGAATTATCTTGCAACACAGCTCTGTTGATTGTCACCATTGTGGTTATTATGCTAGTTCTGGGGTTGGCTGTGGCCTTCTGCTGTCTCCACTTTGATCTGCCCTGGTATCTCAGGATGCTAGGTCAATGCACACAAACATGGCACAGGGTTAGGAAAACAACCCAAGAACAACTCAAGAGAAATGTCCGATTCCACGCATTTATTTCATACAGTGAACATGATTCTCTGTGGGTGAAGAATGAATTGATCCCCAATCTAGAGAAGGAAGATGGTTCTATCTTGATTTGCCTTTATGAAAGCTACTTTGACCCTGGCAAAAGCATTAGTGAAAATATTGTAAGCTTCATTGAGAAAAGCTATAAGTCCATCTTTGTTTTGTCTCCCAACTTTGTCCAGAATGAGTGGTGCCATTATGAATTCTACTTTGCCCACCACAATCTCTTCCATGAAAATTCTGATCATATAATTCTTATCTTACTGGAACCCATTCCATTCTACTGCATTCCCACCAGGTATCATAAACTGAAAGCTCTCCTGGAAAAAAAAGCATACTTGGAATGGCCCAAGGATAGGCGTAAATGTGGGCTTTTCTGGGCAAACCTTCGAGCTGCTATTAATGTTAATGTATCAGCCACCAGAGAAATGTATGAACTGCAGACATTCACAGAGTTAAATGAAGAGTCTCGAGGTTCTACAATCTCTCTGATGAGAACAGACTGCCTATAA

>TLR10Ma

ATGAGACTCATCAGAAACATTTACATATTTTGTAGTATTGTTATGACAGTAGAGGGCGAGGCTCCAGAGCTGCCAGAAGAAAGGGAACTGATGACCAACTGCTCCAACATGTCTCTAAGAAAGGTTCCCGCAGACTTGACCCCAGCCACAACCACACTGGATTTATCCTATAACCTCCTTTTTCAACTCCAGAGTTCAGATTTTCATTCTGTCTCCAAACTGAAAGTTTTGATTCTACGCCATAACAGAATTCAACAGCTGAATCTCAAAACCTTTGAATTCAACAAGGAGTTAAGATATTTAGATTTGTCTAATAACAGACTGAAGAGCGTAACTTGGTATTTACTGGCAGGTCTCAGGTATTTAGATCTTTCTTTTAATGACTTTGACACCATGCCTATCTGTGAGGAAGCTGGCAACATGTCACACCTGGAAATCCTAGGTTTGAGTGGGGCAAAAATACAAAAATCAGATTTCCAGAAAATTGCTCATCTGCATCTAAATAGTGTCTTCTTAGGATTCAGAACTCTTTCTCATTATGAAGAAGGTAGCCTGCCCATCTTAAACACGACAAAACTGCACATTGTTTTACCAATGGACACAAATTTCTGGGTTCTTTTGCGTGATGGAATCAAGACTTCAAAAATATTAGAAATGACAAATATAGATGGCAAAAGCCAATTTGTAAGTTATGAAATGCAACGAAATATTAGTTTAGAAAATGCTAAGACATCAGTTCTATTACTTAATAAAGTTGATTTACTCTGGGACGACCTTTTCCATATCTTACAATTTGTTTGGCATACATCAGTGGAACACTTTCAGATCCAAAATGTGACTTTTGGTGGTAAGGCTTATCTTGACCACAATTCATTTGACTACTCAAATACTGTAATGAGAACTATAAAATTGGAGCATGTACATTTCAGAGTGTTTTACATTCAACAGGATAACATCTATTTGCTTTTGACCAAAATGGACATAGAAAACCTGACAATATCAAATGCACAAATGCCACATATGCTTTTCCCTAATTATCCTACAAAATTCCAATATTTAAATTTTGCCAATAATATCTTAACAGACGAGTTGTTTAAAAGAACTATCCAACTGCCTCACTTGAAAACTCTCCTTTTGAATAGCAATAAACTGGAGACACTTTCTTTAGTGAGTTGCTTTGCTAACAACACACCCTTGGAACACTTGGATCTGAGTCAAAATCTATTACAACATAAAAATGATGAAAATTGCTCATGGCCAGAAACTGTGGTCAACATGAATCTGTCATACAATAAATTGTCTGATTCTGTCTTCAGGTGCTTGCCCAAAGGTATTCAAATACTTGACCTAAATAATAACCAAATCCAAACTGTACCTAAAGAGACTATTCATCTGATGGCCTTACGAGAGCTAAATATTGCTTTTAATTTCCTAACTGATCTCCCTGGGTGCAGTCATTTCAGTAGACTTTCAATTCTGAACGTTGAAATGAACTTAATTCTCAGCCCATCTCTGGATTTTGTTCAGAGCTGCCAGGAAGTTAAGACTCTAAATGCGGGAAGAAATCCATTCCGGTGTACTTGTGAATTAAAAAATTTCATTCAGCTTGAAACATATTCAGAGGTCATGATGGTTGGATGGTCAGATTCTTACACCTGTGAATACCCTTTAAACCTAAGGGGCACTCGGTTAAAAGATGTTCATCTCCCTGAACTATCTTGCAACACAGCTCTGTTGATTGTCACCATTGTGGTTATCATGCTAGTTCTGGGGTTGGCTGTGGCCTTCTGCTGTCTCCACTTTGATCTGCCCTGGTATTTCAGGATGTTAGGTCAATGCACACAAACATGGCACAGGGTTAGGAAGACAACTCAAGAACAACTCAAGAGAAATGTCCGATTCCACGCATTTATTTCATACAGTGAACATGATTCTCTGTGGGTGAAGAATGAATTGATCCCCAATCTAGAGAAAGAAGATGGTTCTATCTTGATTTGCCTTTATGAAAGATACTTTGACCCTGGCAAGAGCATTAGTGAAAATATTGTAAGCTTCATTGAGAAAAGCTATAAGTCCATCTTTGTTTTGTCTCCCAACTTTGTCCAGAATGAGTGGTGCCATTATGAATTCTACTTTGCCTACCACAATCTCTTCCATGAAAATTCTGATCATATAATTCTTATCTTACTGGAACCCATTCCATTCTACTGCATTCCCACAAGGTGTCATAAACTGAAAACTCTCCTGGAAAAAAAAGCATACTTGGAATGGCCCAAGGATAGGCGTAAATGTGGGCTTTTCTGGGCAAACCTTCGAGTTGCTATTAATGTTAACATATCAGCCACCAGAGAAATGTATGAACTGCAGACATTCACAGAGTTAAATGAAGAGTCTCGAGGTTCTACAGTCTCTCTGATGAGAACAGACTGTCTATAA

>TLR10RN

ATGAGACTCATCAGCAGCATCTATACCCTTTATAACATCATTATGTCAACAGAAGCCTGGGCTCCAAGACCCACAGATGAAAGGGAACTGATCTCCAACTACTCCAACATGGCTTTAAGGAAGGTTCCCCCAGATGTGACCCCAGCCACAACCATACTGGATTTATCCTACAACCTCCTCTATCAATTCCAGAGCTCAGATTGCCACTCCTTCTCCAAACTGCGTTTTCTGATCCTGTGCCATAACAGCATCCAACAGCTGGACACCAAGATCTTTGACCTTAACAAGGAGCTAAGCTATTTAGACTTGTCTTACAATAGACTACAGTCCATCACTTGGTCTTCCTTGGTAGATCTCAGACATTTAGATCTTTCTTTTAACGACTTCGATACTCTGCCTGCCTGTGAGGAGATTGGCAACATGCGACATCTGGAAACCTTAGGTTTGAGTGGGGCCAAAATCCAAAGATCAGATCTCCAGAAAATTGCTAATTTGCAATTCAGCACTGTGTTCATAGGGCTGAATACTCTTTCTCACTATGAAGAAGGCAGCCTGCCTGTCCTAAACACGACAGTGCTTCACATAGTTTTACCAATGGCTGCTAACTTCTGGGTGCTTCTGTGTGATGGAATGAAGACTTCAAAGGTAGTCGAAATGTCAAACATAGGTGGCAAGAGCCAGTTTGTAAGTTATGAGACTCCGAGAACTCTTTCTGTGGAGAATACCAAGGCGTCCACTCTTATATTCCATAAAGTTGACCTGCTCTGGGATGACCTTCTCCACGTTTTTCAGTTTGTGTGGCGGACTTCAGTGGAATGCTTCCAGATTCAGGACCTGACTTTTGGAGGGATGGCTAGTCGGGACCACAGTTCATTTGACTACTCAAATACCGTAATGAAAGCTGTAATCTTGGAGAGCATACATTTCAGAATCTTTTTCATTCCCCAGAATAAAATCTACTTGTTTTTCACCCAAATGGATTTAGAAAACCTGACGATATCAGACGCACAAATGCCACATCTTCTCTTCCCTAGTCACCCCCAAAGTTTCCAACATTTAAATTTTGCCAATAACATCTTAACAGATGACCTGTTTAAAAACCCCATCCAGATGCCTTATCTGAAAACTCTCGTTTTGAGGGGCAACCAACTGGAGACTCTTTCTCTAGTGAGTCTCTTTGCCAACCACACGTCCTTGCTTCACCTGGATCTGAGCCAGAACCTATTGCAACATGGAAACGGGGAGAAATGCATGTGGCCCGACACCTTGATCCTCTTGAACTTGTCATCCAATAAATTTGATGGTTCTGTTTTCAGATGTTTGCCTAAAAGTATCCAGACACTTGATCTGAACAATAACAAAATTCGAGCAGTCCCTAAAGAGATTATATACCTAAAATCTTTACGAGAGCTAAGTATTGCTTTTAATTTTCTGACTGACCTCCCTGGGTGTGATCACTTCAAACAGCTCTCTAGTCTGAACATTGAAATGAATTTGATTCTCAGCCCATCTCTGGATTTTTTCCAGAGCTGCCAGGAGGTAAAGACTCTAAACGCAGGGCGAAATCCATTCCGGTGTATCTGTGAATTTAGAGACTTCATTTGGCTTGAGAAACATTCCAAGGGCCTGATGTCTGGATGGCCAGACTCCTACGCCTGCGAATACCCTTTGAGTCTAAAGGGGACTCCATTAAAAGATGTTTATCTTCCCGAATTAGCTTGCAACACAACTTTGCTGATTGTCACCATTGTGCTGGTTATCCTGGTTCTGGGTTTGGTTGTAGCATTCTGCATTCTAAAATTTGACATGCTTTGGTATCTCAGGGTGCTGGCCCAGTGCATCCGGACACAGCATAGGATTAGGAAGACAGTTCGAGAGGAGCGTAAGAGAAGTATACAGTTCCATGCATTTATCTCCTACGCTGCACATGATTCTGTCTGGGTGAAGAATGAACTGATTCCCAATCTGGAGACAGAAGATGGCTCTGTCTTGCTTTGTCTTCCCGAGAGGAACTTTGACCCTGACAGGAGCATTGCTGAAAATATTATAAGCTGCACTGAGAAAAGCTATAAGTCCATCTTTGTGCTGTCCCCCAACTTTGTGCAGAATGGGTGGTGCCATAATGAACTCTATCTGGCCCACCACAATCCTTTCCGTGACAATTATGATTACATCATTCCCATCTTACTAGAACCCATTCCCTCCTACTGTGTTCCTACCAAGTTCCCTAAGCTGAAAGCTCTTTTGGAAAAGAAAGTATACTTAGAGTGGCCCAAGGACAGGCGCAAACATGGGCTTTTTTGGGCAAACCTTCGAGCTGCCATTAACGTAAATTTATCAGACACAAGGGGAAGGTGTGAACTACAGACATTCACAAAGCCTAACGCGGATTGCCAAGGTTCTACCATCTCTCTGGTAAGAACAGACAGCTTCTAA

>TLR10BT

ATGAGATATATCAGAAGCATTTACATATTTTGTAGTATTGTTACGTCTGTGCGTAGTGGGGCTTCAGAGCTGCCCGAAGAAAGGGAATTGACTACCAACTTCTCCAGCATGTCTTTAACAAAGGTTCCTGAAGGCTTGACCCCAATTACAACCACATTGGATTTGTCTTATAATCTCCTTTTTCAACTCCAGCATTCAGATTTCCGTTCTCTCTCTAAACTGAAAGTTTTGATTCTATGCCACAACAGAATCCAAGAGCTGGATATCAAGACCTTTGAATTCAACAAGGAGTTAAGCTACTTAGATGTGTCTAACAACAGACTGAAGAGTGTAACCTGGTTCTCACTGGCAGGTCTCAGACATTTAGATCTGTCCTTCAATGACTTTGACACTCTGCCTATCAGCGTAGAAACTGGCAACATGTCACACCTGGAAACCCTAGGTTTGAGTGGGGCAAAAATACAAAAATCAGATTTTCAGAAAATTGCTCATTTGCAGCTAAATACCGTCTTATTAGGATTGAGAACTCTATCTCATTATGAAGAAGGTAGCTTGCCCATCTTAAACACAACAAGACTTCACATTGTGTTACCAGTGAACACAAATTTCTGGGTTCTTTTGCATGATGGAATCAAAACTTCAAAAATATTAGAAGTCATCAATATAGATTTGCAAAAGAGCCAGTTTACGAGTTATGAATCTCAACAAATTCCTATTTTAGAAAATGCCAAGACATCCATTCTGTTACTTAATAAAGTTGACTTATCCTGGGACGATCTTTTCCTCATCTTCCAATTGGTTTGGCATACATCAGTAGAGTACTTTCAGATTCAACATGTGACCTTTGGAGGTAAGGTTTATCTTGACCACAATTCATTTGACTACTCAAATACTGTAATGAGAACTATAAAACTGGAACATGTACATTTTAGAATTTTTAATATCCCCCAGGAGAGCATCTACTTGCTTTTTACCAAAATGGATATAGAAAACCTGACAATATCAGATGCACAAATGCCTCACATGCTGTTCCCTATGTACCCTACAAGATTCCAATATTTAAATTTCGCTAATAATATCTTAACAGATGATGTGTTTAAAAAATCTATCCAATTGCCTCATTTGAAAACTCTCATCTTGAAGGACAATAAATTGGAGACACTTTCCTTAGTGAGTCACTTTGCCAGCAACACATCCCTGAGGCACTTAGATCTGAGTGAAAATCTGTTACAACATGAAAATGATGAAAATTGCTTGTGGCCAGAAACCTTGGTCACCATGAACTTGTCATTCAACAAATTTGCTGATTCTGTTTTTGGGTGCTTGCCTAGAAACATTCAAATACTGGACCTGAATAGTAACAAAATTCAAACTGTCCCTAAAGCGATTACTCACCTGACATCTTTGCGAGAGTTAAATCTTGCATTTAATTTTCTAACTGATCTTCCTGGGTGCAGTCATTTCAGAAGACTCTTAGTTCTGAACGTTGAAATGAACTTAATTCTCAGCTCATCTCTGGATTTTTTCCAGAGCTGCCAGGAAGTTAAAACTCTAAATGCAGGAAGAAATCCATTCCGATGTACTTGTGAATTAAGAGATTTCATTCAGCTTGGAAAATACTCAGAAGGCATGATGGTTGGATGGTCAGATTCATACATCTGTGAATACCCTTTGAATCTAAAGGGGACTCAGTTAAAGGATGTTCATCTTCCAGAAATATCTTGCAACACAGGTTTGCTGATTGTCACCATTGTGGTTGTCATGCTCGTTCTGGGGATGGCTGTGGCCTTCTGCTGCCTCCACTTTGATTTGCCCTGGTATCTCAGGATGCTGCATCAATGGACTCAGACATGGCTCAGGGTTAGGAAGACAACTCAGGAACAACTCAAAAGAAGTGTCCAATTCCATGTGTTTATTTCATACAGTGAACATGATTCTGCCTGGGTGAAGTATGAATTGATTCCCAGTCTAGAGAAAGAAGATGGTTCTGTTCTGATTTGCCTTCATGAGGGAAACTCTGACCCTGGCAAGAGCATGACTGAAGATACCATAAACTGCATTGAGAAAAGTTACAAGTCCATCTTTGTTTTGTCTCCCAGCTTTGTCCAGACTGAATGGTGCCATTATGAACCCTACTTTGCCCACCACAATCTCTTCCATGAAAGTCTTGATTACATAATTCTTATCTTACTGGAACCCATTCCACTCTACTGCATCCCTACCAGATATCCTGAGCTGAAAGCTCTCATGGAAAAGAAAGCATACTTGGAATGGCCCAAGGATAGGCGTAAATGTGGCCTTTTTTGGGCAAACCTTCGAGCTGCTCTTCATGTTAATTTATTAGACACCAGAGGGACATGTGAACTACAGACATTCACAGAACTGAATGAAGGGTTTGGAGGTTCTGCAATCTCTCTGATCAGAACAGACTGCCTGTAG

>TLR10SS

ATGAAACTTATCAGAAGCATTTACATATTTTGTGGTATTGTCATGTCAGTGCATGGAAGGACTTCAGAGCTGCCAGAAGAAAGGGAATTGACTACCAACTGCTCCAACATGTCTTTAAGAAAGGTTCCCACAGACTTGACCCCAACCACAACCACACTGGATTTATCCTACAACCTCCTTTTTCAACTCCAGCATTCAGATTTTCATTCTTTCTCAAAACTAAAAATTTTGATTCTGTGCCATAACAGAATCCAAGAGCTGGACATCAAGACCTTTGAATTCAACAAGGAGTTACAATATTTAGATGTGTCTTACAATAGACTGAAGACTGTAACTTGGTATTCACTGGCAAGTCTCAGACAGTTGGATCTGTCCTTCAATGACTTTGACAGCATGCCTATCTCCGAGGAGACGGGCAACATGTCACACCTGGAAATCCTGGGTTTGAGTGCGGCCAAAATACGAAAATCAGATTTCCAGAAAATTGCTCATTTGCATCTAAATACTGTCTTTTTAGGATTGAGAACTCTTTCTCATTATGAAGAAGGTAGCCTGCCCATCTTAAACACAACAAAACTTCACATTGTTTTACCAATAAACACAAATTTCTGGGATCTTTTGCGTGATGGAATCAAGACTTCAAAAATATTGGAAATGACGAATCTAGATGGCAAAAGCCAATTTGCAAGTCATGAAACTCAACAAAACCTTATTTTAGAGAATACCAAGACATCCATTCTGTTACTTAATAAAGTTGATTTACCCTGGGATGACCTTTTCCTTATCTTACAATTTGTTTGGCACACATCAGTAGAATACTTCCAGATCCAACATGTGATTTTTGGAGGCAAGGTTTATCTTGATCACAATTCATTTGACTACTCAAATACTGTAATGAGAACTATAAACTTGGAGCATGTACATTTCAGGGTTTTTAATATCCCACAGGAGAGAGTCTACTTGCTTTTTACCAAAATGGCTATAGACAATCTGACAATATTAGATGCACAAATGCCTCACATGTTGTTCCCTACTTATCCTACAAGATTCCAATACTTAAATTTTGCTAGTAATATCTTTACAGATGACGTGTTTAGAAAACCTGTCCAACTGCCTCATTTGAAAACTCTCATTTTGAAGGACAACAAATTGGAGACACTTTCCTTAGTGAGTTGCTTTGTCAGAAACACATCCTTAGAACACTTAGATCTGAGCCAAAATCTGTTACAATATGAAAATGATGAAAAGTGCTTGTGGCCAGAAACTTTGATCACCATGAACTTGTCATTCAATAAACTTGCTGATTCCGTTTTCAGGTGCTTGCCCAGAAATATTCAAATGCTTGACCTGAATAATAACAACATTCACACTGTCCCTAAAGAAATTATTCATCTGACATCCTTACAAGAGTTAAATCTTGCATTTAACTTTCTAACTGATCTTCCTGGGTGCAGTCATTTCAGAAGACTCTCAGTTCTGAACATTGAGATGAACTTAATCCTCAGCCCGTCGGTGGATTTTTTTCAGAGTTGCCAGGAAGTTAAGACTTTAAACGCGGGAGGAAATCCATTCCGGTGTACTTGTGAATTAAGAGATTTCATCCAGCTTGAAAAATATTCAGAGGGCATGATGGTTGGGTGGTCAGATTCATACATCTGCGAATACCCTTTGGATCTAAAGGGGACTCAGCTAAAAGATGTTCATCTTTCTGAATTATCTTGCAACATAGCTTTGTTGATTGTCATCATAGTGATTATCATGCTAGTTCTGGGGATGGCTGTGGCCCTCTGCTGCCTCCGCTTTGATCTGCCCTGGTATCTCAGAATGCTGGGTCAGTGGGCACGGACATGGTACAGGGTTAGGAAAACAACCCAAGGACAACTCAAGAGAAATATTCAATTCCACGTGTTTATTTCATTCTGTGAACATGATTCGGCCTGGGTAAAGCATGAATTGATTCCCAATCTAGAGAAAGAAGATGGTTCTGTCCTGATTTGCTTTCATGAGGGAAACTCTGATCCTGGCAAGAGCATTACTGAAGATATCATAAACTGCATGGAGAAAAGCTCTAAGTCCATCTTTGTTTTGTCTCCCAATTTCGTCCAGAGCGAGTGGTGCCACTATGAACTCTACTTTGGCCACCACCACCTCTTCCATAAAGGGTCTGATGACATAATTCTTATCTTACTGGAACCCATTCCACTCTACTGCATTCCTACCAGGTATCCTGCACTGAAAGCTCTCATGGAAAAGAAAGCATATTTGGAATGGCCCAAGGATAGGCGTAAATGTTGCCTTTTTTGGGCAAACCTTAGAGCTGCTATTCATGTTAATCTATTAGAAACCAGAGAGGTGTGTGAACTACAGACATTCACAGAGCTGAATGAAGGGTCTCGAGGTTCTGCAATATCTCTGATAAAAACAGACTGCCTATAA

>TLR10CF

ATGAAACGTATCAGAAGCATTTATATATTTTGTAGTATTGCTATATCAGTGCAGGGCTGGGCTTCGAAGCTGCCAGAAGAAAGGGAGTTGACAACCAATTGCTCCAACATGTCCCTAAGAAAAATTCCTGCAGACTTGACCCCAACCACAACCACACTGGATTTATCCTACAACCTCCTTTCTCAACTCCAGAGTTCAGATTTTCGTTCTGTCTCTAAACTGAAGGTTTTGATTCTCTGCCATAACAGAATTCAAGAGCTGAATATCAAGATCTTTGAATTTAACAGAGAGTTAAGATATTTAGATTTGTCTTACAACAGATTGAAGATTGTAACTTGGTATTCACTGGCAGGTCTCAGGCATTTGGATCTTTCTTTCAATGACTTTGACACCGTGCCTATCTGTGAGGAGACTGGCAACATGTCACATTTGGAAATCCTAGGTTTGAGTGGGGCAAAAATACAAAAATCAAATTTCCAGAAAATTGCTCATTTGCATCTAAAAACTGTCTTCTTAGGATTGAGAAGTCTTTCTCACTATGAAGAAGGTAGCCTGCCCATCTTAAACACAACAAAACTTCACATTGTTTTACCAATGAACACAAATTTCTGGGTTCTTTTGCATGATGGAATCAAGACTTCAAAAATACTAGAAATGACAAATATAGATGGCAAAAGCCAATTTGCAAGTTATGGAACTCAAAAAAATCTTACTTTAGAGAATTCCAAGACATCTATTTTATTACTTAATAAAGTTGATTTACTCTGGGATGACCTTCTCCTCATCTTCCAATTTGTTTGGCATACGTCAGTAGAATGCTTCCAAATCCAACATCTGACTTTTGGAGGCAAGGTTTATCTCGACCACTATTCATTTGATTACTCAAACACTGTAATGAGAACTATAAAATTAGAGCATGTACAGTTCAGAATTTTTTATATTCCACAGGAGAGAGTCTACTTGCTCTTTACCAAAATGGATATAGAAAACCTGACTATATCAGATGCACAAATGCCATATATGCTGTTTCCTATATATCCTACAAGATTCCAATATTTAAATTTCGCTAATAATATCTTAACGGATGACCTGTTCAAGCAACCTATCCAATTACCTCATTTGAAAACTCTCATTTTGAAGGGCAATAAATTGGAGACGTTTTCTTTAGTGAGTTTCTTTGCCAACAACACATCCTTGAAGCACTTAGATCTGAGCCAGAATCTGTTACAACATGAAAATGGTGAAAATTGCTTTTGGCCAGAAACCTTGATCACTATGAACCTGTCATCCAACAAATTTGCTGATTCTGTTTTCAGGTGCTTGCCCAGAAATATTCAAATACTTGACTTGAATAATAACAAAATTCAAACTGTCCCTAAAGACATTATTCATCTGAAGTCTTTGCAAGAGTTAAATCTTGCATTTAATTTTCTAACTGATCTTCCAGGATGCAGTCATTTCAGAAAACTTTCAATTCTGAACATTGAAATGAACTTAATTCTCAGCCCATCTCTGGATTTTTTTCAGAGCTGTCAGGAAGTTAAGATTCTGAATGCAGGAAGAAATCCATTCCGGTGTACTTGTGAATTAAGAGATTTCATTCAGCTTGAAGAATATTCAGAGGGCATGATGATTGGATGGTTAGATTCATATATCTGTGAATATCCTTTGACTCTAAAGGGGACTCTGCTAAAGGACGTTCATCTTCCTGAGTTATCGTGTAACACAACTCTGTTGATTGTCACCATTGTGGTTATTATGCTAGTTCTGGGGATGGCTGTGGCCTTCTGCTGCTTCTACTTTGATCTGCCCTGGTATCTCAGGATGCTAGGTCAATGGACATTGCAGAGGATTAGAAAAACAACCCAAGAACAGCTCAAGAGAAATGTCCAGTTCCATGTGTTTATTGCATACAGTGAACATGATTCTACCTGGGTGAAGCACGAATTAATCCCCAATCTAGAGAAGAAAGAGAAATTGATTTGCCTTCATGAGGGAAACTTTGACCCTGGCAAGAGCATTATTGAAAATATCATGAACTGCATTGAGAAAAGCTATAAGTCCATCTTTGTTTTGTCTCCCAACTTTGTCCAGAGTGAGTGGTGCCATTATGAACTTTACTTCGCCCACCACAGTCTCTTCCATGAAAATTCTGATTACATAATTTTTATCTTACTGGAACCCATTCCACTCTACTGCATTCCTACCAAGTATCCTAAGCTGAAAGCTCTTATGGAAAAGAAAGCATACTTGGAATGGCCCAAGGATAGGCGTATATGTGGACTTTTTTGGGCAAATCTTCGAGCTGCTATTAATGCTAACTTATTAGAAACCAGAGAGATGTATGAACTACAGACATTTGTGGGGCTGAATGAAGAGTCTCAAGGTTCTGCAATCTCTCTGATAAGAACTGACTGCCTATAA

**Alignment of TLR1 and TLR6 cDNA sequences.**

* denotes a nucleotide that is conserved in TLR1 and TLR6 of all species

TLR1HS ---------------------ATGACTAGCATCTTCCATTTTGCCATTATCTTCATGTTA 39

TLR1PT ---------------------ATGCCTAGCATCTTCCATTTTGCCATTATCTTCATGTTA 39

TLR1Ma ---------------------ATGACTAGCATCTTCCATTTTGCCATTATCTTTATGTTA 39

TLR1MM ---------ATGACTAAACCAAATTCCCTCATCTTCTACTGTATCATTGTTTTAGGACTG 51

TLR1RN ---------ATGACTAAAACACAGTCCACCATCTTCTATTGTATTGTTGTCTTAGGGCTG 51

TLR1EE ---------ATGACTAAAACTTACTCTATTGTCTTCCATTTAATCATCATCTTCATGCTA 51

TLR1BT ---------ATGACTAAAAAAAATTCTAGCATCTTCCATTTTGCCATCATCTTTATATTA 51

TLR1SS ---------ATGACTAAAGAGAATCTTAGCATCTTCCATTTTGCCATCATCTTCATATTA 51

TLR1CF ---------ATGATGAAAACTAATCCTAGCATCTTCCAATTTGCCATCATCTTCATATTA 51

TLR6HS ATGACCAAAGACAAAGAACCTATTGTTAAAAGCTTCCATTTTGTTTGCCTTATGATCATA 60

TLR6PT ATGACCAAAGACAAAGAACCTATTGTTAAAAGCTTCCATTTTGTTTGCCTTATGATCATA 60

TLR6Ma ATGACCAAAGACAAAGAACCTGTTGTTAAAAGCTTCCATTTTGTTTGCCTTATGATCATA 60

TLR6MM ATGAGCCAAGACAGAAAACCCATCGTGGGGAGTTTCCACTTTGTTTGCGCCCTGGCCTTA 60

TLR6RN ATGTCCCAAGACAGAGAACCCATCGTGGAGAGTTTCCATTTTGTTTGCACCCTGGCCTTA 60

TLR6EE ATGACCAGAGACAAAGAATCTACTATGAGAAG------TTTTGCTTATACTGTGATCGTA 54

TLR6BT ATGATCAAAGACAAAGAATCTCCCATCAGAAGCTGTCATTTTGTTTACATTGTGGCCTTA 60

TLR6SS ATGAGCAAAGACAAAGAACCTACTGTCATAAGCCTTCATTCTGTGTATGTCATGACCTTA 60

TLR6CF ATGATCAAAGACAAAGACTCTATCACTGGAAGCTTCCATTTTGTGTACATTGTGACCTTA 60

* * *

TLR1HS ATACTTCAGATCAGAATACAATTATCTGAAGAAAGTGAATTTTTAGTTGATAGGTCAAAA 99

TLR1PT ATACTTCAGATCAGAATACAATTATCTGAAGAAAGTGAATTTTTAGTTGATAGGTCAAAA 99

TLR1Ma ACACTTCAGATCAGAATACAATTATCTGAAGAAAGTGAATTTTTAGTTGATAGGTCAAAA 99

TLR1MM ACACTT---ATGAAAATCCAATTATCTGAGGAATGTGAGCTTATCATAAAGAGGCCAAAC 108

TLR1RN ATACTT---ATCAAAATCCAGTTATCTGAGGAAAGTGAGTTGATCATTAAGAGGCCAAAT 108

TLR1EE ATAGTTAAGATCAGAACTCTACTATCTGATGGAAGTGATGTTTTAGCTGACAGATCAAAC 111

TLR1BT ATACTTGAGATCAGAACTCAATTATCTGATGAAAGTGAATTTTTAATTGACAGGTCAAAA 111

TLR1SS ATACTTGAGATCAGAATTCAATTATCTGAGGAAAGTGAAGTTTTAGTTGACAGATCAAAA 111

TLR1CF ATACTTGAGATCAGAATACAATTGTCTGAAGAAAGTGATTTTCTAGTTAACAGATCAAAA 111

TLR6HS ATAGTTGGAACCAGAATCCAGTTCTCCGACGGAAATGAATTTGCAGTAGACAAGTCAAAA 120

TLR6PT ATAGTTGGAACCAGAATCCACTTCTCCGACGGAAATGAATTTGCAGTAGACAAGTCAAAA 120

TLR6Ma ATAGTTGGAACTAGAATCCAGTTCTCTGACGGAAGTGAATTTGCAGTAGACAAGTCAAAA 120

TLR6MM ATAGTCGGAAGCATGACCCCGTTCTCTAATGAACTTGAGTCTATGGTAGACTATTCAAAC 120

TLR6RN ATAGTCGGAAGCATGACCCAGTTCTCTGATGAATTTGAGTCTGTAGTAGACTATTCAAAC 120

TLR6EE ACAGTTGGGATCGTAATCCAGTCCTCTGATGAAAATGAATTTACAGTAGATTTGTCCAAA 114

TLR6BT GTATTTGGAACCATAATCCAGTTCTCTGATGAAAGTGAATTTGTGGTAGACATGTCAAAA 120

TLR6SS GTATGGGGAACCCTAATCCAGTTCTCTGAAGAAAGTGAATTTGTGGTAGACAAGTCAAAA 120

TLR6CF ATAGTTGGAACCATAATCCAATTCTCTGATGAAAGTGAATTTACAGTAGACATGTCAAAT 120

* * * * ** * * * *** * * **

TLR1HS AACGGTCTCATCCACGTTCCTAAAGACCTATCCCAGAAAACAACAATCTTAAATATATCG 159

TLR1PT AACGGTCTCATCCACGTTCCTAAAGACCTGTCCCAGAAAACAACAATCTTAAATATATCG 159

TLR1Ma AACAGTCTCATCCACGTTCCTAAAGACCTATCCCAGAAAACAACAATCTTAAATATATCA 159

TLR1MM GCAAACCTTACCAGAGTGCCCAAGGACCTACCCTTGCAAACAACTACTTTAGATCTATCA 168

TLR1RN GCAAACCTTACCAGAGTGCCCAAGGACCTACCCTTGCAAACAACTACTTTAGATGTATCA 168

TLR1EE AGGACTCTCATTCATATTCCCAAGGATCTACCCCCCAGTACGACAATCCTAAATGTTTCA 171

TLR1BT AGAGGTCTCACCTATGTTCCCAAAAACTTATCCCTGGAAACAACCATCTTAGATATATCA 171

TLR1SS ACAGGTCTCACCCATGTTCCCAAAGACCTATCCTTGGAAACGACAATCTTAGATCTATCA 171

TLR1CF GCAGGTCTCTTTCACATTCCCAAAGACCTATCCCTGAAAACAACAATCTTAGATATATCA 171

TLR6HS AGAGGTCTTATTCATGTTCCAAAAGACCTACCGCTGAAAACCAAAGTCTTAGATATGTCT 180

TLR6PT AGAGGTCTTATTCATGTTCCAAAAGACCTACCGCTGAAAACCAAAGTCTTAGATATGTCT 180

TLR6Ma AGAGGTCTTACTCATGTTCCAAAAGACCTACCGCCGAAAACCAAAGTCTTAGATATGTCT 180

TLR6MM AGGAACCTTACTCATGTCCCCAAAGACCTGCCACCAAGAACAAAAGCCCTGAGTCTGTCT 180

TLR6RN AAGAACCTTACTCATGTCCCAAAAGACCTGTCACCAAGCACAAAATCCTTGAGTCTGTCT 180

TLR6EE AGAGGCCTCACTCATATTCCAAGAGATTTGCCATCCCAAACCGAAGTCTTAGATATGTCT 174

TLR6BT ACAAGCCTTATTCATGTTCCCAAAGACCTGCCACCAAAAACCAAAGTCTTAGACTTGTCT 180

TLR6SS ATAGGCCTTACTCGTGTTCCAAAAGACCTGCCACCCCAAACCAAAGTCTTAGATGTGTCT 180

TLR6CF ATGAACCTTACTCATGTCCCAGAAGACCTGCCACCAAAAACCAAAATCTTAGATATGTCT 180

** * ** * * * ** * * **

TLR1HS CAAAATTATATATCTGAGCTTTGGACTTCTGACATCTTATCACTGTCAAAACTGAGGATT 219

TLR1PT CAAAATTATATATCTGAGCTTTGGACTTCTGACATCTTATCACTGTCAAAACTGAGGATT 219

TLR1Ma CAAAATTATATATCTGAGCTTTGGACTTCTGACATCTTATCACTGTCAAAGCTGAGGATT 219

TLR1MM CAAAACAATATATCTGAGCTTCAGACTTCTGACATCCTCTCATTGTCCAAGCTGAGGGTC 228

TLR1RN CAAAACAATATATCTGAGCTTCAGACTTCTGACATACTCTTGTTGTCCAAACTGAGGGTC 228

TLR1EE CACAACTATATATCTGAGCTTTGGGCTTCTGACATCCTGTCACTCTCAAAGCTGAAGATC 231

TLR1BT TACAACTATATTTCTGAGCTTCAGATGCCTGACATCCTCTCACTATCAAAGCTGAAGATT 231

TLR1SS CAAAACTCTATCTCTGAGCTTCAGACTTCTGACATCCTCTCACTATCAAAGCTGAGGGTT 231

TLR1CF CAAAACTATATATCTGAGCTTCAGACTTCTGACATCCTATCACTATCAAAGCTGAGGATT 231

TLR6HS CAGAACTACATCGCTGAGCTTCAGGTCTCTGACATGAGCTTTCTATCAGAGTTGACAGTT 240

TLR6PT CAGAACTACATAGCTGAGCTTCAGGTCTCTGACATGAGCTTTCTATCAGAGTTGAAAGTT 240

TLR6Ma CACAACTACATAGCTGAGCTTCAGGTCTCTGACATAAGCTTTCTGTCAGAGTTGAAAGTT 240

TLR6MM CAAAACTCTATATCTGAGCTTCGGATGCCTGATATCAGCTTTCTGTCAGAGCTGAGAGTT 240

TLR6RN CAAAACTCCATATCTGATCTTCAGATGTCTGATATCAGCTTTCTGTCAGAGCTGAGAGTT 240

TLR6EE CAAAACAACGTATCTGAACTTCACCTTTCTGATATGAGCCTTATCTCAAGACTCAAAGTT 234

TLR6BT CAAAACAACATATCTGAGCTTCACCTGTCTGATATCAGCTTTCTCTCAGGGCTGAGAGTT 240

TLR6SS CAAAACTTCATAACTGAGCTTCACCTCTCTGACATCAGCTTTCTCTCGCAGCTGACGGTT 240

TLR6CF CAGAACAATATATCTGAGCTTCACCTCTCTGACATGAGCTATCTCTCAGGACTAAAAATA 240

* ** * **** *** **** ** * ** * * *

TLR1HS TTGATAATTTCTCATAATAGAATCCAGTATCTTGATATCAGTGTTTTCAAATTCAACCAG 279

TLR1PT TTGATAATTTCTCATAATAGAATCCAGTATCTTGATATCAGTGTTTTCAAATTCAACCAC 279

TLR1Ma TTGATAATTTCTCATAATAGACTCCAGTATCTTGATATCAGTGTTTTCAAATTCAACCAG 279

TLR1MM CTGATAATGTCCTACAACAGACTCCAGTATCTTAATATCAGTGTTTTCAAATTCAACACA 288

TLR1RN TTCATTATGTCCTACAACAGACTCCAGTATCTTAATATCAGTGTTTTCAAATTCAACACG 288

TLR1EE TTGATAATGTCTCATAACAGAATCCAGAATCTTGATATTAGTGTTTTCAGATTCAACCAG 291

TLR1BT TTGATAATTTCTCATAATAGAATCCAGTATCTTGACTTGAGTGTTTTTAAATTCAACCAG 291

TLR1SS TTCATAATTTCTCATAATAGAATACAGTATCTTGATGTCAGTGTTTTCAAATTCAACCAG 291

TLR1CF TTGATTGTTTCTTATAATAGAATTCAATATCTTGATATCAGTGTTTTCAAATTCAACCAG 291

TLR6HS TTGAGACTTTCCCATAACAGAATCCAGCTACTTGATTTAAGTGTTTTCAAGTTCAACCAG 300

TLR6PT TTGAGACTTTCCCATAACAGAATCCAGCTACTTGATTTAAGTGTTTTCAAGTTCAACCAG 300

TLR6Ma TTGAGACTTTCCCATAACAAAATCCAGCTACTTGATTTAAGTGTTTTCAAGTTCAACCAG 300

TLR6MM CTGAGACTCTCCCACAACAGGATACGGAGCCTTGATTTCCATGTATTCTTGTTCAATCAG 300

TLR6RN CTGAGACTCTCCCACAACAGAATACGGAGACTTGACTTTGGTGTGTTCTTGCTCAATCGG 300

TLR6EE TTAAGACTTTCCCATAACAGGATCCAGTGCCTTGATTTTAGTGTTTTCAGATTCAACCAG 294

TLR6BT CTGAGACTTTCCCATAATAGAATCCAGGGCCTTGATATTAGTATTTTCAAGTTCAACCAT 300

TLR6SS TTGAGACTTTCCCAGAATAGGATGCAGTGCCTTGATATCAGTGTTTTCAAGTTCAATCAG 300

TLR6CF TTGAGAATTTCTCATAATAGAATCTGGTGGCTTGATTTTAGCATTTTCAAGTTCAACCAG 300

* * * ** * ** * * *** * * * ** ****

TLR1HS GAATTGGAATACTTGGATTTGTCCCACAACAAGTTGGTGAAGATTTCTTGCCACCCTACT 339

TLR1PT GAATTGGAATACTTGGATTTGTCCCACAACAAGTTGGTGAAGATTTCTTGCCACCCTACT 339

TLR1Ma GAATTGGAATACTTGGATTTGTCCCACAACAAGTTGGCGAAAATTTCTTGCCACCCTACT 339

TLR1MM GAGCTGGAATATTTGGATTTGTCCCACAATGAGCTAAAGGTGATCTTGTGCCACCCAACA 348

TLR1RN GAACTGGAATATTTGGATTTGTCCCACAATGAGTTAAGGCTGATCTCTTGCCACGCAACA 348

TLR1EE GAATTGGAATACTTGGATTTATCTCACAACAAATTGGAGACAATTTCTTGCCACTCCACT 351

TLR1BT GAACTGGAATACTTGGATTTGTCCCACAACAATTTGGAGAAGATTTCTTGCCACCCTACT 351

TLR1SS GAACTGGAATACTTGGATTTGTCCCACAACAAGTTGGAGAAGATTTCGTGCCACCCTATG 351

TLR1CF GAATTGGAATACTTGGATCTGTCCCACAATGAGTTGGGGAGGATTTCTTGCCATCCTACC 351

TLR6HS GATTTAGAATATTTGGATTTATCTCATAATCAGTTGCAAAAGATATCCTGCCATCCTATT 360

TLR6PT GATTTAGAATATTTGGATTTATCTCATAATCAGTTGCAAAAGATATCCTGCCATCCTATT 360

TLR6Ma GACTTGGAATATTTGGATTTATCTCATAATCAGTTGCAAAAGATATCCTGCCATCCTATT 360

TLR6MM GACTTAGAATACCTGGATGTCTCACACAATCGGTTGCAAAACATCTCTTGCTGCCCTATG 360

TLR6RN GACTTAGAATACCTGGATGTCTCTCACAATCAGTTACAAAACATCTCTTGCTGTCCTATG 360

TLR6EE GATTTGGAATATTTGGATTTATCCCACAATCAGTTGAAAAAATTGTCCTGCCATGCCATC 354

TLR6BT GATTTGGAATATTTGGATTTATCTCATAATCAGTTGCAGAAGATATCCTGCCATCCAATC 360

TLR6SS GATTTGGAATATTTGGATTTATCTCACAATCAGTTGCAGACAATCTTGTGCCATCCCATC 360

TLR6CF GATTTGGAATATTTGGATTTATCTTACAATCAGTTACGGAACATGTCCTGCCATCTTATC 360

** * ***** ***** * ** * ** * * * *** *

TLR1HS GTGA---ACCTCAAGCACTTGGACCTGTCATTTAATGCATTTGATGCCCTGCCTATATGC 396

TLR1PT GTGA---ACCTCAAGCACTTGGACCTGTCATTTAATGCATTTGATGCCCTGCCTATATGC 396

TLR1Ma GTGA---ACCTCAAGCACTTGGACCTGTCCTTTAATGCATTTGATGCCCTGCCTATATGC 396

TLR1MM GTCA---GCCTCAAGCATTTGGACCTCTCCTTTAATGCCTTTGATGCCCTGCCTATATGC 405

TLR1RN GCCG---ACCTCAAACATTTAGACCTCTCCTTTAATGCATTTGATGCCCTGCCCATATGC 405

TLR1EE GCGA---ACCTTAAACATTTGGATCTCTCCTTCAATGCATTTGTTTTCCTACCCATATGC 408

TLR1BT CTGA---ACCTCAAGCACTTAGACCTCTCATTTAATCCATTTGATGCCCTGCCCATATGC 408

TLR1SS CTGA---ACCTCAAGCACTTGGACCTCTCATTTAATGCATTCGATGCCCTGCCCATATGC 408

TLR1CF GTGA---ACCTCAAGCACTTAGACCTTTCATTTAATGCATTTGATGATCTACCCATATGC 408

TLR6HS GTGA---GTTTCAGGCATTTAGATCTCTCATTCAATGATTTCAAGGCCCTGCCCATCTGT 417

TLR6PT GTGA---GTTTCAGGCATTTAGATCTCTCATTCAATGATTTCAAGGCCCTGCCCATCTGT 417

TLR6Ma ATGA---GTTTCAGGCATTTAGATCTCTCATTCAATGACTTCGAGGCCCTGCCCATCTGT 417

TLR6MM GCGA---GCCTGAGGCATCTAGACCTCTCATTCAATGACTTTGATGTACTGCCTGTGTGT 417

TLR6RN GTGA---ACTTGAAACATCTAGACCTCTCATTCAATGACTTTGAAGTGCTGCCCGTGTAT 417

TLR6EE CAGA---GTCTCCAGCATTTAGACCTCTCCTTCAATGACTTTGATAGTTTGCCCATTTGT 411

TLR6BT ACCACGACTCTCAAGCATTTAGACCTCTCATTCAATGACTTCGATGCCCTGCCCATCTGT 420

TLR6SS ACCA---GCCTCAAGCATTTGGACCTCTCATTCAATGACTTTGAAGCCCTGCCCATATGT 417

TLR6CF AGGA---GTCTCAAGCATTTAGACCTCTCATTCAATGACTTTCATGTCCTGCCCATCTGT 417

* ** * ** ** ** ** *** ** * ** * *

TLR1HS AAAGAGTTTGGCAATATGTCTCAACTAAAATTTCTGGGGTTGAGCACCACACACTTAGAA 456

TLR1PT AAAGAGTTTGGCAATATGTCTCAACTAAAATTTCTGGGGTTGAGCACCACACACTTAGAA 456

TLR1Ma AAAGAGTTTGGCAATATGTCTCAACTAAAATTTCTGGGGTTGAGTACTACACACTTAGAA 456

TLR1MM AAAGAATTTGGCAACATGTCCCAACTACAGTTCCTGGGGTTGAGCGGTTCTCGGGTACAA 465

TLR1RN AAAGAGTTTGGCAACCTGTCCCAACTACAGTTTCTGGGATTGAGCGGTTCTCAGATACAA 465

TLR1EE AAAGAGTTTGGTAACATGTCCCAGCTAGAATTTCTAGGGTTGAGTGCAAGCCAGTTACAA 468

TLR1BT CAAGAGTTTGGCAACATGTCTCAACTAGAATTTCTGGGGTTGAGTGCCACACAGTTACAG 468

TLR1SS CAAGAGTTTGGCAGTATGTTTCAACTAGAATTTCTGGGGTTGAGTGCCACACAGTTACAA 468

TLR1CF AAAGAGTTTGGCAACATGTCTCAACTAGAGTTTCTGGGGTTGAGTGCCACACAGTTACAG 468

TLR6HS AAGGAATTTGGCAACTTATCACAACTGAATTTCTTGGGATTGAGTGCTATGAAGCTGCAA 477

TLR6PT AAGGAATTTGGCAACTTGTCACAACTGAATTTCTTGGGATTGAGTGCTATGAAGCTGCAA 477

TLR6Ma AAGCAATTTGGCAACTTGTCACAACTGAATTTCTTGGGGTTGAGTGCTATGAAGTTACAA 477

TLR6MM AAGGAATTTGGCAACCTGACGAAGCTGACTTTCCTGGGATTAAGTGCTGCAAAGTTCCGA 477

TLR6RN AAGGAATTTGGCAACTTGATGAAGCTGAGTTTCTTGGGATTAAGTGCTGCAAAGTTCCGA 477

TLR6EE AAGGAATTTGGCAACTTGACACAACTGAATTTCTTGGGATTAAGTACCTCAAAGTTGCAA 471

TLR6BT AAGGAATTTGGCAACTTGACCCAACTGAATTTCTTAGGATTAAGTGCTACAAAGTTACAA 480

TLR6SS AAGGAGTTTGGCAACTTGACACAACTGAATTTCTTGGGATTAAGTGCTACAAAGTTACAG 477

TLR6CF AAGGAATTTGGCAACTTGACACAACTACAATTCTTAGGTTTAAGTGCTACAAAGTTACGG 477

* * ***** * * * ** ** * ** ** ** *

TLR1HS AAATCTAGTGTGCTGCCAATTGCTCATTTGAATATCAGCAAGGTCTTGCTGGTCTTAGGA 516

TLR1PT AAATCTAGTGTGCTGCCAATTGCTCATTTGAATATCAGCAAGATCTTGCTGGTCTTAGGA 516

TLR1Ma AAATCTACTGTGCTGCCAATTGCTCATTTGAATATCAGCAAGGTCTTGCTGGTCTTAGGA 516

TLR1MM AGTTCAAGTGTGCAGCTGATTGCTCATTTGAACATCAGTAAGGTTTTGCTGGTGTTAGGA 525

TLR1RN AATTCAAGTGTGCAGCTGATTGCTCATCTGAACATCAGTAAGGTTTTGCTGGTGTTAGGA 525

TLR1EE AAGTCTAGGTTGCTGTCAATTTCCCATCTGCACATCAGTAAGGTTTTACTGGTCTTAGGA 528

TLR1BT AAATCCAGTGTGCAGTCAATCACTCATTTGCACATCAGCAAGGTTTTATTGGTCTTAGGA 528

TLR1SS AAATCCAGTGTGTTGCCAATCGCTCATTTGCATATCGGTAAGGTTTTACTTGTCTTAGGA 528

TLR1CF AAATCTAGCATGCTACCAATTGCTTCTTTGCATATCAGAAAGGTTTTACTGGTCTTAGGA 528

TLR6HS AAATTAGATTTGCTGCCAATTGCTCACTTGCATCTAAGTTATATCCTTCTGGATTTAAGA 537

TLR6PT AAATTAGATTTGCTGCCAATTGCTCACTTGCATCTAAGTTATATCCTTCTGGATTTAAGA 537

TLR6Ma AAATTAGATTTACTGCCAATTGCTCACTTGCATCTAAGTTACATCCTTCTGGATTTAAGA 537

TLR6MM CAACTGGATCTGCTCCCAGTTGCTCACTTGCATCTAAGCTGCATTCTTCTGGACTTAGTG 537

TLR6RN CAACTGGATCTGCTCCCAATTTCTCACTTGCACCTGAGCTGTGTTCTTCTGGACTTGGTG 537

TLR6EE CAGTTAGACCTGCTACCAGTTTCTCACTTGCATGTAGACAGCATCCTTCTGGATTTACAA 531

TLR6BT CAATTAGATTTACTACCCATTGCTCACTTGCACCTAAGTTGTATCCTTCTGGATTTGGAA 540

TLR6SS CAATTAGATCTACTACCAATTGCTCACTTGCATCTAAGTTGCATCCTTCTGGATTTGGAA 537

TLR6CF CAATTAGATCTGCTACCAATTGCTCATTTGCATCTAAGTTATATCCTTCTGGATTTACAA 537

* * * ** * * * * * * **

TLR1HS GAGACTTATGGGGAAAAAGAAGACCCTGAGGGCCTTCAAGACTTTAACACTGAGAGTCTG 576

TLR1PT GAGACTTATGGGGAAAAAGAAGACCCTGAGGGCCTTCAAGACTTTAACACTGAGAGTCTG 576

TLR1Ma GAGCATTATGGGGACAAAGAAGACCCTGAGGGCCTTCAAAACTTTAACACTGAGAGTCTG 576

TLR1MM GATGCTTATGGGGAAAAAGAAGACCCCGAATCTCTTCGGCACGTTAGCACTGAGACTCTG 585

TLR1RN GACACTTATGGGGAAAAAGAAGATCCCAAGTGTCTTCAGCACATTAGCACTGAGACTCTG 585

TLR1EE GACTCCTATGGGGAAAAAGAAATCCCTGACAGCCTTCAAGACCTGAACACAGAAAGTCTT 588

TLR1BT GATACTTATGGGGAAAGAGAAGATGCCGAGAGCCTTCAAGACCTTAAGACACAGAGTCTG 588

TLR1SS GACTCTTACGGGGAAAGAGAAGACCCTGAGAGCCTTCAAGACCTTAACACACAGAGTCTG 588

TLR1CF GACACTTATGGGAAAAAAGAAGACCCTGAGAGCCTTCAAAAGCTTAACACAGAAAGTCTT 588

TLR6HS AATTATTATATAAAAGAAAATGAGACAGAAAGTCTACAAATTCTGAATGCAAAAACCCTT 597

TLR6PT AATTATTATATAAAAGAAAATGAGACGGAAAGTCTACAAATTCTGAATGCAAAAACCCTT 597

TLR6Ma AATTATTATATAAAAGAAAATGAGACAGAAAGTCTACAAATTCTGAATGCAAAAACACTT 597

TLR6MM AGTTATCATATAAAAGGCGGGGAAACAGAAAGTCTTCAGATTCCCAATACCACCGTTCTC 597

TLR6RN AATTATCAGATAAAAGATGGTGAAACAGAAAGTCTTCAGGTTCCAAATACCAACGTTCTC 597

TLR6EE GGCTATTCTATGAAAGGAAACGAGACTGGGAGTCTTCAAATTTTCAATACAAAAAAACTT 591

TLR6BT GACTAT---ATGAAAGAAAATAAGAAAGAAAGTCTTCAAATTCTGAATACAAAGAAACTT 597

TLR6SS CGTTATTACATGAAAGAAAATGAGAAAGAAAGTCTTCAAATTCTGAACACAAAGAAACTT 597

TLR6CF GGTTATTATGCAAAAGAAAGTGAAAAAGGAAGTCTTCAAATTCTAGATACAAAAACACTT 597

* ** * * **

TLR1HS CACATTGTGTTCCCCACAAACAAAGAATTCCATTTTATTTTGGATGTGTCAGTCAAGACT 636

TLR1PT CACATTGTGTTCCCCACAAACAAAGAATTCCATTTTATTTTGGATGTGTCAGTCAAGACT 636

TLR1Ma CACATTGTGTTCCCAACAAGCAAAGAATTCAATTTTATTTTGGATGTGTCAGTCAGGACT 636

TLR1MM CATATTGTTTTCCCGTCGAAAAGAGAATTCCGTTTTCTTCTGGATGTGTCCGTCAGCACT 645

TLR1RN CATATCGTTTTCCCTTCCAAAAGAGAATTCCATTTTCTTCTGGACATGTCTGTCAGCACG 645

TLR1EE CACATTGTTTTTCCCCTGGGGAAGGAGTTCCATTTTAATTTGGATGTGTCAATCAGTCAG 648

TLR1BT CACATTGTTTTCCCCACAGGAAAGGAATTCCATTTTATTTTGGACGTGTCAGTCGGCACC 648

TLR1SS CACATTGTTTATCCCCCAGGAAAGGAATTTCATTTTATGTTGGACGTGTCAGTCAGCACC 648

TLR1CF CACATTGTTTTCCCTATAAGAAAGGAATTCAGTTTTACTCTGGATGTATCAGTCAGCACT 648

TLR6HS CACCTTGTTTTTCACCCAACTAGTTTATTCGCTATCCAAGTGAACATATCAGTTAATACT 657

TLR6PT CACCTTGTTTTTCACCCAACTAGTTTATTCGCTATCCAAGTGAACATATCAGTTAATACT 657

TLR6Ma CACCTTGTTTTTCACCCAACTAGTTTATTCTCTATCCAAGTGAACATATCAGTTAATACT 657

TLR6MM CATTTGGTCTTTCATCCAAATAGCTTGTTCTCTGTTCAAGTGAACATGTCTGTAAACGCT 657

TLR6RN CATTTGGTCTTTCATCCGAATAGCCTGTTCTCTGTGCAAGTGAACATATCTGTAAATGCT 657

TLR6EE CACTTTGTTTTTCATCCAAATGATTTGTTCTCTGTGCTAGTGAACATATCTGTTAACATG 651

TLR6BT CACCTTGTTTTTCACCCAAATAGCTTTTTCTCTGTCCAAGTGGACATATCAGCGAATAGT 657

TLR6SS CACCTGGTCTTTCATCCAAATAGCTTCTTCTCTGTCCAAGTAAACATATCGGTTAAGAGT 657

TLR6CF CATCTTGTTTTTCATCCAAATCAGTTATTCTCTGTACAAGCAAACATGTTAGTTAATAAT 657

** * ** * * ** * * * * *

TLR1HS GTAGCAAATCTGGAACTATCTAATATCAAATGTGTGCTAGAAGATAACAAATGTTCTTAC 696

TLR1PT GTAGCAAATCTGGAACTATCTAATATCAAATGTGTGCTAGAAGATAACAAATGTTCTTAC 696

TLR1Ma GTAGCAAATCTGGAACTATCTAATATCAAATGTGTGCTAGAAGATAACGAATGTTCTTAC 696

TLR1MM ACGATCGGTTTGGAACTGTCTAACATCAAGTGTGTGCTTGAAGACCAGGGCTGCTCTTAT 705

TLR1RN GCCATCAGTTTGGAACTGTCTAACATCAAGTGTGTGCTTGAGGACAAGAACTGCTCTTAC 705

TLR1EE GCAGTAAGTCTGGAACTGTCTAATATCCAGTATGTGCTGGAAGATGGTGGTGCATGTTCT 708

TLR1BT ACAGTGAGTCTGGAACTGTCTAATATCAAATGTGTGCTTGATGATAATGGGTGTCCTTAT 708

TLR1SS GCAGTAAATCTGGAACTGTCCAATATCAGATGTGTGCTGGATGCTAACGGATGTCATCAC 708

TLR1CF GCAGTAAGTCTCGAATTGTCTAATATCAAATGTGTGCCAGATGGTCATGGATGGTCTTAT 708

TLR6HS TTAGGGTGCTTACAACTGACTAATATTAAAT------TGAATGATGACAACTGTCAAGTT 711

TLR6PT TTAGGGTGCTTACAACTGACTAATATTAAAT------TGAATGATGACAACTGTCAAGTT 711

TLR6Ma TTAGGGTGCTTACAACTGACTAATATTAAAT------TGAATGATGACAACTGTCAAGTT 711

TLR6MM TTAGGACATTTACAACTGAGTAATATTAAAT------TGAATGATGAAAACTGTCAAAGG 711

TLR6RN TTAGGATGCTTACAACTGAGTAATATTAAAT------TGAATGATAAAAACTGTCAAAGC 711

TLR6EE ATAGAGTGCTTACAGCTGACTAATATCAAAC------TGAATGATGACAACTGTCAAGTT 705

TLR6BT TTAGGGTGCTTACAACTGACTAATATTAAAT------TGAATGATTACAATTGTCAAGTT 711

TLR6SS GTAGGGTGTTTACAACTGGCTAATATTAAAC------TGAGTGATGACAACTGTCAGGTT 711

TLR6CF TTAGGGTGCTTACAACTGACTAATATTAAGT------TGAATAATGACAACTGTCAAGTT 711

* * * ** **

TLR1HS TTCCTAAGTATTCTGGCGAAAC---TTCAAACAAATCCAAAGTTATCAAATCTTACCTTA 753

TLR1PT TTCCTAAGTATTCTGGCGAAAC---TTCAAACAAATCCAAAGTTATCAAGTCTTACCTTA 753

TLR1Ma TTCCTAAATATTCTGGCAAAAC---TTCAAACAAATCCAAAGTTATCAAGTCTTACTTTA 753

TLR1MM TTCTTACGTGCTTTGTCAAAGC---TTGGAAAGAATCTGAAGCTCTCAAATCTTACCCTG 762

TLR1RN TTCCTAGGTACCTTAGAAAGAC---TTAGAAAGACTCAGAGGCTCTCAAATCTTACCCTG 762

TLR1EE TTCCAAAATGCTCTGAGGAAAC---TTCAGAAGAATCCAAGGCTATCAAATCTTACTTTA 765

TLR1BT TTCGAAAATGTTCTGTCAAAAC---TTCAAAAGAACTCAAGGTTATCAAATCTTACTTTA 765

TLR1SS TTCCAAAATGTTCTATTGAAAC---TTCAAAAGAACTCAAAGTTATCCAACCTGACTTTG 765

TLR1CF TTCCAAAATGTTCTGTCAAAAC---TTCAAAAGAATTCAAGGTTATCAAGTCTTACTTTA 765

TLR6HS TTCATTAAATTTTTATCAGAAC---TCACCAGAGGTTCAACCTTACTGAATTTTACCCTC 768

TLR6PT TTCATTAAATTTTTATCAGAAC---TCACCAGAGGTCCAACCTTACTGAATTTTACCCTC 768

TLR6Ma TTCATTAAATTTTTATTAGAAC---TCACTAGAGGTCCAACCTTGCTGAATTTTACCCTC 768

TLR6MM TTAATGACATTTTTATCAGAAC---TCACCAGAGGTCCAACCTTATTGAATGTGACCCTC 768

TLR6RN TTAATTATATTTTTATCAGAAC---TCACCAGAGGTCCAACCTTATTGAATCTGACCCTC 768

TLR6EE TTAATAAATTTCTTATCAGAACCACTTATCAGAGGCCCAAACTTACTGAATTTGACCCTA 765

TLR6BT TTACTTAAATTTTTATCAGGAC---TCACTGGAGGACCAACCTTACTAAATTTTACCCTC 768

TLR6SS TTCATTACATTTTTATTGGAAC---TCACTCAAGGGCCAACCTTACTAAATTTTACGCTC 768

TLR6CF TTAATTCAATTTTTATCAGAAC---TCACCAGAGGTCCAACTTTACTGAATTTTACTCTC 768

** * * * * * * * ** *

TLR1HS AACAACATTGAAACAACTTGGAATTCTTTCATTAGGATCCTCCAGCTGGTTTGGCATACA 813

TLR1PT AACAACATTGAAACAACTTGGAATTCTTTCATTAGGATCCTCCAGCTGGTTTGGCATACA 813

TLR1Ma AACAACATTGAAACAACTTGGAATTCTTTCATTAGGATCCTCCAGCTGGTTTGGCATACA 813

TLR1MM AACAATGTGGAAACAACGTGGAATTCCTTCATTAATATCCTCCAGATAGTTTGGCATACG 822

TLR1RN AACAACGTGGACACAACATGGAATTCCTTCATTAACATCCTTCAGCTGGTTTGGCATACA 822

TLR1EE AATAACATTGACACCACTTGGAATTCCTTCATGATGATTCTCCAGTTGGTTTGGCACACA 825

TLR1BT AACAACATTGAAATAACTTGGAATTCCTTCTTCACGATCCTCCAGTTGGTTTGGCGTACA 825

TLR1SS AACAACATTGAAACCACTTGGAATTCTTTCATCACCACCCTCCAGTTTGTCTGGCATACA 825

TLR1CF AACAACATTGAAACAACTTGGAATTTTTTCATTATGCTCCTTCAGTTGGTTTGGCATACA 825

TLR6HS AACCACATAGAAACGACTTGGAAATGCCTGGTCAGAGTCTTTCAATTTCTTTGGCCCAAA 828

TLR6PT AACCACATAGAAACGACTTGGAAATGCTTGGTCAGAGTCTTTCAATTTCTTTGGCCCAAA 828

TLR6Ma AATCACATAGAAACAACTTGGAAATGCCTGGTGAGAGTCTTTCAATTTCTTTGGCCCAAA 828

TLR6MM CAGCACATAGAAACAACCTGGAAGTGCTCGGTTAAACTTTTCCAATTCTTTTGGCCCCGA 828

TLR6RN CAGCACATAGAAACAAACTGGAAGTGCTTTGTTAGACTTTTACAATTCCTTTGGCCCAGA 828

TLR6EE GATCATGTGGAAACAACTTGGAAATGTTTAGTTAGAGTTTTTCAGTCCCTTTGGTCCAAG 825

TLR6BT AACCACATGGAAACAACTTGGAAATGTTTGGTTAAAGTTTTTCAGTTCCTTTGGCCCAAA 828

TLR6SS AACCATGTGGAAACAACTTGGAAATGTTTGGTTGGAATTTTTCAATTCCTTTGGCCCAAA 828

TLR6CF CAACATGTGAAAACAACTTGGAAATGCTTGGTTAGAATTTTTAAATTTCTTTGGCCCAAA 828

* * * * * * ***** * * * * * ***

TLR1HS ACTGTATGGTATTTCTCAATTTCAAACGTGAAGCTACAGGGTCAGCTGGACTTCAGAGAT 873

TLR1PT ACCGTATGGTATTTCTCAATTTCAAACGTGAAGCTACAGGGTCAGCTGGACTTCAGAGAT 873

TLR1Ma ACCGTATGGTATTTCTCAATTTCAAACGTGAAGCTACAGGGTCAACTGGACTTCAGAGAT 873

TLR1MM CCAGTCAAATATTTCTCAATTTCAAATGTGAAGCTACAAGGTCAACTTGCCTTCAGGATG 882

TLR1RN CCAGTCAAGTCTTTCTCAATTTCAAACGTGAAACTAAAAGGTCATTTTAACTTCAGAAGA 882

TLR1EE GGCGTAGAATATTTCTCAATTAAAAATGTGAAACTACAAGGTTGGTTTCATCCCAGAGAG 885

TLR1BT AACATTGAGTACTTCTCCATTTCAAATGTGAAACTACAAGGTTACCTTGACTCTAGAGAT 885

TLR1SS AGCATCGAGTATTTCTCCATTTCCAGTGTGAAACTACAAGGTCAGCTGGACTTCAGAGAT 885

TLR1CF AGCATAGAGTATTTCTCAATTTCAAATGTAAAACTACAAGGTTACCCTGACTTCAGAGAT 885

TLR6HS CCTGTGGAATATCTCAATATTTACAATTTAACAATAATTGAAAGCATTCGTGAAGAAGAT 888

TLR6PT CCTGTGGAATATCTCAATATTTACAATTTAACAATAATTGAAAGCATTCATGAAGAAGAG 888

TLR6Ma CCTGTGGAATATCTCAATATTTACAATTTAACAATAATTGAAAGCATTCATGAAGAAGAT 888

TLR6MM CCGGTGGAGTACCTCAATATTTACAACTTAACGATAACTGAGAGAATCGACAGGGAAGAA 888

TLR6RN CCTGTGGAGTATCTCAATATTTACAACTTAACGATAACTGAGAGCATAAGCAGGGAGACA 888

TLR6EE CCTATAGAATACCTGAATATTTACAATTTGACAGTAGTTGAAAGAATTGATGAAGAAGAG 885

TLR6BT CCTATAGAATATCTCAATATTTACAATTTAACAATAGTTGAAAGCATTGATGAAGAAGTT 888

TLR6SS CCTGTAGAATATCTCAGTATTTACAATTTAACAATAGTTGAAAGCATTGATGAAGAAGAT 888

TLR6CF CCTGTACAATATCTCAATATTTATAATTTAACAATAGTTGAAAGCATTAATAAGGAATAT 888

* * * *** * * * ** *

TLR1HS TTTGATTATTCTGGCACTTCCTTGAAGGCCTTGTCTATACACCAAGTTGTCAGCGATGTG 933

TLR1PT TTTGATTATTCTGGCACTTCCTTGAAGGCCTTGTCTATACACCAAGTTGTCAGCGATGTG 933

TLR1Ma TTTGATTATTCTGGCACTTCCCTGAAGGCCTTGTCTGTACACCAAGTCGTCAGCGATGTG 933

TLR1MM TTCAATTATTCTGACACTTCTCTGAAGGCTTTGTCGATACATCAAGTTGTCACTGATGTC 942

TLR1RN TTCCATTATTCTGACACTTCTCTGAGGGCTTTGTCGATACATCAAGTTGTCACTGATGTG 942

TLR1EE TTTAATTATTCTGACACTTCACTGAAATCCTTGACTATACATCAGGTTGTCAATAATGCA 945

TLR1BT TTTGATTATTCTGACACTTCACTGAAGGCCTTGTCTATACACAAAGTTGTCCATGATGTG 945

TLR1SS TTTGATTATTCTGACACGTCACTGAAGGCCTTGTCTCTACACCAAGTTGTCAGTGAGGTG 945

TLR1CF TTTGATTATTCTGACACTTCACTGAAGGCCTTATCTATACACCAAGTCGTTAGTAATGCA 945

TLR6HS TTTACTTATTCTAAAACGACATTGAAAGCATTGACAATAGAACATATCACGAACCAAGTT 948

TLR6PT TTTACTTATTCTAAAACGACATTGAAAGCATTGAAAATAGAACATATCACGAACAAAGTT 948

TLR6Ma TTTACTTATTCTAAAACGACATTGAAAGCATTGAAAATAGAACATATCACGAACCAAGTT 948

TLR6MM TTTACTTACTCGGAGACAGCACTGAAGTCACTGATGATAGAGCACGTCAAAAACCAAGTG 948

TLR6RN TTTATTTACGTGGAGACGGTGTTGAAGTCACTGAAGATAGAGCATGTCACAAACCAAGTG 948

TLR6EE TTCACTTACTCCAAAACATCATTGAAGGCACTGAAAATAGAGCATATTACAAATAGAGTT 945

TLR6BT TTTACTTATTATAAAACGACATTGAAGGCACTGAAAATAGAACATATTACAAACAAAGTT 948

TLR6SS TTTATTTATTATGAAACAACATTGAAAGGAGTGAAAATAGAACATATTACAAAGAGAGTT 948

TLR6CF ATTCATTATCCTAAAACAGCACTGAAAGCATTGACAATAGAGCATGTTAAAAATGAAGTT 948

* *** ** *** * ** * * * *

TLR1HS TTCGGTTTTCCGCAAAGTTATATCTATGAAATCTTTTCGAATATGAACATCAAAAATTTC 993

TLR1PT TTCAGTTTTCCGCAAAGTGATATCTATGAAATCTTTTCGAATATGAACATCAAAAATTTC 993

TLR1Ma TTCAATTTTCCACAAAGGGATATCTATGAAATCTTTTCAAATATGAACATCAAAAATTTC 993

TLR1MM TTCAGCTTCCCCCAAAGTTACATATACAGTATCTTTGCCAATATGAACATCCAAAACTTT 1002

TLR1RN TTCAGCTTCCCCCAAAGTAACATATACAGCATCTTCTCCAATATGAACATCCAAAGTTTT 1002

TLR1EE TACAGTTTAGAACAAAATTCTATCTACAAAATCTTTGCAAATATGAACATCCAACATTTC 1005

TLR1BT TTCAGTCTTCCACAAGGTTATGTCTATAAAATATTGTCAAATATGAACATCCAGCATCTC 1005

TLR1SS TTCAGTTTCCCACAAAGTTACATCTATAAAATCTTTTCAAATATGAACATTCAGTATCTC 1005

TLR1CF TTCAATTTGCCACAAAGTTATATCTATAAAATCTTTTCAAATATGAACATCCAAAATTTC 1005

TLR6HS TTTCTGTTTTCACAGACAGCTTTGTACACCGTGTTTTCTGAGATGAACATTATGATGTTA 1008

TLR6PT TTTCTGTTTTCACAGACAGCGTTGTACACCGTGTTTTCTGAGATGAACATTATGATGTTA 1008

TLR6Ma TTTATCTTTTCGCAGACAGCATTGTACACCGTGTTTTCTGAGATGAACATTATGATGTTA 1008

TLR6MM TTCCTCTTTTCAAAGGAGGCGCTATACTCGGTGTTTGCTGAGATGAACATCAAGATGCTC 1008

TLR6RN TTCCTCTTTGTGAAGGATGCACTATATTCTGTGTTTGCAGAAATGAACATCAGGATGCTC 1008

TLR6EE TTTCTTTTTTCACAGACTGCATTGTACACAGTGTTTTCTGAGATGAACATTATGATGTTA 1005

TLR6BT TTTATTTTTTCACAGACAGCATTATACACAGTGTTTTCTGAGATGAACATTCTGATGTTA 1008

TLR6SS TTTATTTTTTCACAGACAGCATTATACAGAGTGTTTTCCGATATGAATATCAGGATGTTA 1008

TLR6CF TTTCTTTTTTCACAGACAGCGTTATACACAATTTTTTCTGAGATGAACATTATGATGTTA 1008

* * * * ** * ** * * ***** ** *

TLR1HS ACAGTGTCTGGTACACGCATGGTCCACATGCTTTGCCCATCCAAAATTAGCCCGTTCCTG 1053

TLR1PT ACAGTGTCTGGTACACGCATGGTCCACATGCTTTGCCCATCCAAAATTAGCCCGTTCCTG 1053

TLR1Ma ACAGTGTCTGGTACACGCATGATCCACATGGTTTGCCCATCCAAAATCAGCCCGTTCCTG 1053

TLR1MM ACAATGTCTGGAACACACATGGTCCACATGCTGTGCCCGTCCCAAGTTAGCCCATTTCTG 1062

TLR1RN ACAGTATCTGGAACACGCATGGTCCACATGCTTTGCCCAGACCAAATTAGCCCATTTCTG 1062

TLR1EE ACAGTGTCTGGTACACCTATGGTCCACATGCTTTGCCCATTACAAACTAGCCCATTTCTG 1065

TLR1BT ACAGTGTCTGCTGCACACATGGTCCACATGGTCTGCCCATCCCAAATTAGCCCATTTCTG 1065

TLR1SS ACAGTGTCTGCCACACACATGGTCCACATGGTTTGCCCATCCCAAATTAGCCCATTTCTA 1065

TLR1CF ACAGTGTCTGGTACGCACATGGTCCACATGGTTTGCCCATCTCAAATTAGTCCATTTCTG 1065

TLR6HS ACCATTTCAGATACACCTTTTATACACATGCTGTGTCCTCATGCACCAAGCACATTCAAG 1068

TLR6PT ACCATTTCAGATACACCTTTTATACACATGCTGTGTCCTCATGCACCAAGCACATTCAAG 1068

TLR6Ma ACCATTTCAGATACACCTTTTATACACATGCTGTGTCCTCGTGCACCAAGCACATTCAAG 1068

TLR6MM TCTATCTCAGACACCCCTTTCATCCACATGGTGTGCCCGCCATCCCCAAGCTCATTTACA 1068

TLR6RN ACACTGTCAGACACGCCATTCATCCACATGGTGTGCCCTGAGTTCCCAAGCACATTTGCA 1068

TLR6EE ACCATATCAGAGGCACCTTTAATACACATGCTTTGTCCTCAGGCACCAAGCATGTTCAAG 1065

TLR6BT ACCATATCAGACACACGCTTTATACACATGCTTTGTCCTCAGGAACCAAGCACATTTAAG 1068

TLR6SS ACCATAGCAGACACACATTTTATACACATGCTTTGTCCTCAGGTACCAAGCACATTTAAC 1068

TLR6CF ACCATATCAGACACACCTTTTATACACATGCTTTGTCCTCCACCATCAAACACATTTAAG 1068

* * * * * * * * ****** * ** ** * **

TLR1HS CATTTGGATTTTTCCAATAATCTCTTAACAGACACGGTTTTTGAAAATTGTGGGCACCTT 1113

TLR1PT CATTTGGATTTTTCCAATAATCTCTTAACAGACACGGTTTTTGAAAATTGTGGGCACCTT 1113

TLR1Ma CATTTGGATTTTTCCAATAATCTCTTAACAGACACGGTTTTTGAAAATTGTGGGCACCTT 1113

TLR1MM CATGTGGACTTTACAGATAACCTTTTAACAGACATGGTTTTTAAAGACTGTAGAAACTTA 1122

TLR1RN TATTTGGACTTTACAGATAACCTTTTAACAGACATAGTTTTTGAAGACTGTAGAAATTTA 1122

TLR1EE TATTTGGATTTTTCTAATAATCTCTTGACAGACATGATTTTCAAAGATTGTGGAAATTTG 1125

TLR1BT TATTTGAATTTTTCCAATAATCTCTTAACAGACACAGTTTTCATAAACTGTACAAATTTG 1125

TLR1SS TATTTGGATTTTTCCAATAATGCCTTAACAGACATGGTTTTTAAAAATTGTGCAAACTTG 1125

TLR1CF CATTTGGATTTTTCTAATAATCTCTTAACAGACATTGTTTTTAAAAATTGTAGAAACTTG 1125

TLR6HS TTTTTGAACTTTACCCAGAACGTTTTCACAGATAGTATTTTTGAAAAATGTTCCACGTTA 1128

TLR6PT TTTTTGAACTTTACCCAGAACGTTTTCACAGATAGTATTTTTGAAAAATGTTCCACGTTA 1128

TLR6Ma TTTTTGAACTTTACCCAGAATGTTTTCACAGATAGTATTTTTGAAAAATGTTCCACGTTA 1128

TLR6MM TTTCTGAACTTTACCCAGAATGTTTTTACTGACAGTGTTTTTCAAGGCTGTTCCACCTTA 1128

TLR6RN TTTCTGAACTTTACCCAGAACGTTTTTACTGACAGCATTTTTCAAGGCTGTTCCACCTTG 1128

TLR6EE TTTTTGAACTTTACCCACAATGTTTTTACAGATAGTATTTTTCAAAACTGTTCCACACTA 1125

TLR6BT TTTTTGAACTTTACCCAGAATAGTTTCACAGATAGTGTCTTTCAAAATTGTGACACTTTA 1128

TLR6SS TTTTTGAACTTTACCCAGAATGTTTTTACAGATAGTGTTTTTCAAAATTGCAAAACTTTA 1128

TLR6CF TTTTTGAACTTTACCCAGAATGTTTTCACAGATAGTGTTTTTCAAAGTTGTTCCCACCTA 1128

* ** * *** * * ** ** ** ** * * ** * ** *

TLR1HS ACTGAGTTGGAGACACTTATTTTACAAATGAATCAATTAAAAGAACTTTCAAAAATAGCT 1173

TLR1PT ACTGAGTTGGAGACACTTATTTTACAAATGAATCAATTAAAAGAACTTTCAAAAATAGCT 1173

TLR1Ma ACTGAGTTGGAGACACTTATTTTACAAATGAATCAATTAAAAGAACTTTCAAAAATAGCT 1173

TLR1MM GTTAGATTGAAAACACTTAGTTTACAAAAGAATCAGTTAAAAAACCTTGAGAATATAATC 1182

TLR1RN ATTAGATTGAAAACACTTAGTTTACAAAAGAATCAGTTAAAAACACTTGAAAATATAATT 1182

TLR1EE ACCAAATTAGAGACTCTTATTTTACAAATGAATCAACTACAAGAATTTACAAAGATAGTT 1185

TLR1BT GCTAATTTGAAGACACTTATCCTACAAAAGAATCAGTTAAAAGAACTTGTAAACATAGTT 1185

TLR1SS GCTAATTTGAACACACTCAGTTTACAAATGAATCAGTTAAAAGAACTTGTGAATGTCATC 1185

TLR1CF ATTAAACTGGAGACACTTAGTTTACAAATGAATCAATTAAAAGAACTTGCAAGTATAGCT 1185

TLR6HS GTTAAATTGGAGACACTTATCTTACAAAAGAATGGATTAAAAGACCTTTTCAAAGTAGGT 1188

TLR6PT GTTAAATTGGAGACACTTATCTTACAAAAGAATGGATTAAAAGACCTTTTCAAAGTAGGT 1188

TLR6Ma GTTAAATTGGAGACACTTATCTTACAAAAGAATGGATTAAAAGACCTTTTCAAAGTAGGT 1188

TLR6MM AAGAGATTGCAGACACTTATCTTACAAAGGAATGGTTTGAAGAACTTTTTTAAAGTAGCT 1188

TLR6RN AAGAGACTGGAGACACTTATCTTGCAAAGGAATGGTTTAAAGAACCTTTTTAAGGTAGCT 1188

TLR6EE GGTAGATTAGAAACACTTATCTTACAGAAGAATGAATTGAAAGAACTTTTTAAAGTAGGT 1185

TLR6BT GCTAGATTGGAGACACTTATCTTACAAAAGAATGAATTAAAAGACCTTTTCAAAACAAGT 1188

TLR6SS GCTAGATTAGAGACACTCATCTTACAAAAGAATAAATTAGAAGACCTTTTCAAAATAAGC 1188

TLR6CF GTTAGATTGGAAACACTTATCTTACGAAAGAATAAATTGAAAGACCTTTACAAAGTAGGT 1188

* * ** ** * * * * **** * * ** *

TLR1HS GAAATGACTACACAGATGAAGTCTCTGCAACAATTGGATATTAGCCAGAATTCTGTAAGC 1233

TLR1PT GAAATGACTACACAGATGAAGTCTCTGCAACAATTGGATATTAGCCAGAATTCTGTAAGC 1233

TLR1Ma GAAATGACTACACGGATGAAGTCTCTGCAACAATTGGATATTAGCCAGAATTCTGTAAGC 1233

TLR1MM CTCACATCTGCAAAGATGACATCCCTACAAAAACTAGACATTAGCCAGAATTCTCTAAGG 1242

TLR1RN CTTATGTCTATGGAGATGACATCCCTACAAAAACTAGACATTAGCCAGAATTCTCTAAGG 1242

TLR1EE TACATGACCAAGAAGATGAAGTCTCTACAACTACTGGATATTAGCCAGAATTCTCTAAGG 1245

TLR1BT CATATGACCCAGGAAATGAAGTCTCTACAACAACTGGATGTTAGCCAGAATTCCCTGATG 1245

TLR1SS CATATGACCAAAGAAATGCAGTCTCTACAGCAATTGGATGTTAGCCAGAATACCCTGAGG 1245

TLR1CF CAAATGACCAACGAGATGAAGTCTCTACAACAATTGGATATTAGCCAGAATTCTCTAAGG 1245

TLR6HS CTCATGACGAAGGATATGCCTTCTTTGGAAATACTGGATGTTAGCTGGAATTCTTTGGAA 1248

TLR6PT CTTATGACTAAGGATATGCCTTCTTTGGAAATACTGGATGTTAGCTGGAATTCTTTGGAA 1248

TLR6Ma CTCATGACTAAGGATATGCCATCTTTGGAAATACTGGATGTTAGCTGGAATTCTTTGGAA 1248

TLR6MM CTCATGACTAAGAATATGTCCTCTCTGGAAACTTTGGATGTTAGTTTGAATTCTTTGAAC 1248

TLR6RN CTCATGACCAAGACTATGTCCTCTCTGGAAACATTGGATGTCAGTTTGAATTCTTTGAAC 1248

TLR6EE CTAATGACTAAGGATATGCAGTCTTTGGAAATACTGGATGTAAGCTGGAATTCTCTGAAC 1245

TLR6BT CTCATGACTAAGGATATGCTTTCTTTGGAAACACTGGATGTTAGCTGGAATTCTTTGGAA 1248

TLR6SS CTCATGACTAAGGATATGCTATCTTTGGAAATACTGGATGTTAGCTCGAATTCTTTGGAG 1248

TLR6CF CTCATGACGAAGCATATGACATCTTTGGAAATATTGGATGTTAGTGTGAATTCTTTGGAA 1248

* * *** ** * * * ** * ** **** * *

TLR1HS TATGATGAAAAGAAAGGAGACTGTTCTTGGACTAAAAGTTTATTAAGTTTAAATATGTCT 1293

TLR1PT TATGATGAAAAGAAAGGAGACTGTTCTTGGACTAAAAGTTTATTAAGTTTAAATATGTCT 1293

TLR1Ma TATGATGAAAAGAAAGGAGATTGCTCTTGGACTAAAAGTTTATTAAGTTTAAATATGTCT 1293

TLR1MM TACAGCGATGGGGGAATCCCATGCGCCTGGACCCAGAGTTTGTTAGTTTTAAATTTGTCT 1302

TLR1RN TACAGTGATGCGGGAAGCCCATGCTCCTGGACCCAGAGTTTGTTAGTTTTAAATTTGTCT 1302

TLR1EE ATTGATGAAAATGAAGGAAATTGTTCTTGGACAGAAAGTTTATCAAGTTTAAATTTATCT 1305

TLR1BT TATGATGAAAGTGAAGGAAATTGCCCTTGGGCCAGAAATTTATTAAGTTTAAATATGTCT 1305

TLR1SS TATGATGAAAATGAGGGAAGTTGCACTTGGACCGGAAGTTTATTAAGTTTAAATTTATCT 1305

TLR1CF TATGATGAAAATGAAGGAAACTGCTCTTGGACTAGAAGTTTATTAAGTTTAAATATGTCT 1305

TLR6HS TCTGGTAGACATAAAGAAAACTGCACTTGGGTTGAGAGTATAGTGGTGTTAAATTTGTCT 1308

TLR6PT TCTGGTAGACATAAGGAAAACTGCACTTGGGTTGAGAGTATAGTGGTGTTAAATTTGTCT 1308

TLR6Ma TCTGGTAGACATAGGGAAAACTGCACTTGGGTTGAGAGTATAGTGGTGTTAAATTTGTCT 1308

TLR6MM TCTCATGCATATGACAGGACATGCGCCTGGGCTGAGAGCATATTGGTGTTGAATTTGTCT 1308

TLR6RN TCTCATGTGTATGACAGAACATGTGCTTGGGCCGAGAGCATACGGGTGTTGAATTTGTCT 1308

TLR6EE TATGATAGATATGATGGAATTTGTACGTGGGCTCAGAGTATAGTGATGTTAAATTTATCT 1305

TLR6BT TATGACAGAAGTAATGGAAATTGCTCTTGGGTTGGGAGTATAGTGGTGTTAAATTTATCT 1308

TLR6SS TATGATAGACATGGTGAAAATTGCACTTGGGTTGGGAGTATAGTGGTGTTAAATTTATCT 1308

TLR6CF TATGATAGATATGATGGAAATTGCACTTGGGTTGGGAGTATAGTGGTGTTAAATTTGTCT 1308

** * *** * * ** *** * ***

TLR1HS TCAAATATACTTACTGACACTATTTTCAGATGTTTACCTCCCAGGATCAAGGTACTTGAT 1353

TLR1PT TCAAATATACTTACTGACACTATTTTCAGATGTTTACCTCCCAGGATCAAGGTACTTGAT 1353

TLR1Ma TCAAATATACTTACTGACACTATTTTCAAATGTTTACCTCCCAGGATCAAGGTACTTGAT 1353

TLR1MM TCGAATATGCTTACAGGCTCTGTCTTCAGATGCTTACCTCCCAAAGTCAAGGTCCTTGAC 1362

TLR1RN TCAAACATGCTTACGGACTCTGTCTTCAGGTGCTTACCTCCCAAGGTCAAGGTCCTTGAC 1362

TLR1EE TCAAATATACTTACTGAGTCTGTTTTCAGATGTCTACCTCCCAGGGTCAAGGTCCTTGAT 1365

TLR1BT TCAAATATACTTACTGACTCTGTTTTCAGATGTTTACCTCCTCAGATCAAGGTTCTTGAT 1365

TLR1SS TCAAATATACTCACTGACTCTGTTTTCAGATGTTTACCTCCCAGGATCAAGGTTCTTGAT 1365

TLR1CF TCAAATATACTTACTGACTCTGTTTTCAGATGTTTACCTCCCAAGGTCAAGGTGCTTGAT 1365

TLR6HS TCAAATATGCTTACTGACTCTGTTTTCAGATGTTTACCTCCCAGGATCAAGGTACTTGAT 1368

TLR6PT TCAAATATGCTTACTGACTCTGTTTTCAGATGTTTACCTCCCAGGATCAAGGTACTTGAT 1368

TLR6Ma TCAAATATACTTACTGACTCCGTTTTCAGATGTTTACCTCCCAGGATCAAGGTACTTGAT 1368

TLR6MM TCGAATATGCTTACAGGCTCTGTCTTCAGATGCTTACCTCCCAAGGTCAAGGTCCTTGAC 1368

TLR6RN TCGAATGTACTTTCGGACTCTGTCTTCAGGTGCTTACCTCCCAAGGTCAAGGTCCTTGAC 1368

TLR6EE TCAAATATACTTACTGAGTCTGTTTTCAGATGTCTACCTCCCAGGGTTAAGGTCCTTGAT 1365

TLR6BT TCAAATGCACTCACTGACTCTGTTTTCAGATGTTTACCTCCTCGGATCAAGGTTCTTGAT 1368

TLR6SS TCAAATATACTCACTGACTCTGTTTTCAGATGTTTACCTCCCAGGATCAAGGTTCTTGAT 1368

TLR6CF TCAAATATACTTACTGACTCTGTTTTCAGATGTTTACCTCCCAAGGTCAAGGTGCTTGAT 1368

** ** ** * * * * **** ** ******* * ***** *****

TLR1HS CTTCACAGCAATAAAATAAAGAGCATTCCTAAACAAGTCGTAAAACTGGAAGCTTTGCAA 1413

TLR1PT CTTCACAGCAATAAAATAAAGAGCGTTCCTAAACAAGTCGTAAAACTGGAAGCTTTGCAA 1413

TLR1Ma CTTCACAGCAATAAAATAAAGAGCATTCCTAAACAAGTCATAAAACTGGAAGCTTTGCAA 1413

TLR1MM CTTCACAACAACAGGATAATGAGCATCCCTAAAGATGTCACCCACCTGCAGGCTTTGCAG 1422

TLR1RN CTTCACAACAACAGGATAGTGAGCATCTCTAAAGATGTCACCCACCTGCAAGCTTTGCAG 1422

TLR1EE CTTCACAGTAACAGAATAAGGAGCATCCCAAGAGATGTCAACAATCTGGAAGCTTTGCAA 1425

TLR1BT CTTCACAATAACAGAATAAGGAGCATCCCTAAAGATGTCACTGGTCTAGAAACTTTGCAA 1425

TLR1SS CTTCACAATAACAGAATAAGGAGCATCCCTAAGGATGTCGCCCATCTGGAAGCTCTGCAA 1425

TLR1CF CTTCACGATAACAGAATAAGGAGCATTCCTAAACCAATCATGAAGCTAGAAGATTTGCAA 1425

TLR6HS CTTCACAGCAATAAAATAAAGAGCGTTCCTAAACAAGTCGTAAAACTGGAAGCTTTGCAA 1428

TLR6PT CTTCACAGCAATAAAATAAAGAGCGTTCCTAAACAAGTCATAAAACTGAAAGCTTTGCAA 1428

TLR6Ma CTTCACAACAATAAAATAAAGAGCATTCCTAAACAAGTCGTAAAACTGGAAGCTTTGCAA 1428

TLR6MM CTTCACAACAACAGGATAATGAGCATCCCTAAAGATGTCACCCACCTGCAGGCTTTGCAG 1428

TLR6RN CTTCACAACAACAGGATAGTGAGCATCCCTAAAGATGTCACCCACCTGCAAGCTTTGCAG 1428

TLR6EE CTTCACAGTAACAGAATAAGGAGCATCCCAAGAGATGTCAACAATCTGGAAGCTTTGCAA 1425

TLR6BT CTTCACAATAACAGAATAAGGAGCATCCCTAAAGATGTCACTGGTCTAGAAACTTTGCAA 1428

TLR6SS CTTCACAGTAACAGAATAAGGAGCATCCCTAAGGATGTCGCCCATCTGGAAGCTCTGCAA 1428

TLR6CF CTTCACGATAACAGAATAAGGAGCATTCCTAAACCAATCATGAAGCTAGAAGATTTGCAA 1428

****** ** * *** **** * * * ** ** * * ****

TLR1HS GAACTCAATGTTGCTTTCAATTCTTTAACTGACCTTCCTGGATGTGGCAGCTTTAGCAGC 1473

TLR1PT GAACTCAATGTTGCTTTCAATTCTTTAACTGACCTTCCTGGATGTGGCAGCTTTAGCAGC 1473

TLR1Ma GAACTCAATGTTGCTTTCAATTCTTTAACTGACCTTCCTGGATGTGGCAGCTTTAGCAGC 1473

TLR1MM GAACTCAATGTAGCATCCAACTCCTTAACTGACCTTCCTGGGTGCGGGGCCTTCAGCAGC 1482

TLR1RN GAACTCAATGTCGCATCCAATTTTTTAACTGACCTTCCTGGATGTGGAGCCTTCAGTAGC 1482

TLR1EE GTACTCAATGTTGCTTCCAATTTTTTAACCAACCTTCCTGGATGTGGTGCCTTTAGCAGC 1485

TLR1BT GAACTCAACCTTGCTTCCAATTCTTTAGCCCACCTTCCTGGATGTGGTATCTTTAGCAGC 1485

TLR1SS GAACTCAATGTTGCTTCCAATTCTTTAGCTCACCTGCCTGGATGTGGTTCCTTTAGCAGC 1485

TLR1CF GAACTCAATGTTGCTTCCAATTCTTTAGCCCACTTTCCTGACTGTGGTACTTTTAATAGG 1485

TLR6HS GAACTCAATGTTGCTTTCAATTCTTTAACTGACCTTCCTGGATGTGGCAGCTTTAGCAGC 1488

TLR6PT GAACTCAATGTTGCTTTCAATTCTTTAACTGACCTTCCTGGATGTGGCAGCTTTAGCAGC 1488

TLR6Ma GAACTCAATGTTGCTTTCAATTCTTTAACTGACCTTCCTGGATGTGGCAGCTTTAGCAGC 1488

TLR6MM GAACTCAATGTAGCATCCAACTCCTTAACTGACCTTCCTGGGTGTGGGGCCTTCAGCAGC 1488

TLR6RN GAACTCAATGTCGCATCCAATTTTTTAACTGACCTTCCTGGATGTGGAGCCTTCAGTAGC 1488

TLR6EE GTACTCAATGTTGCTTCCAATTTTTTAACCAACCTTCCTGGATGTGGTGCCTTTAGCAGC 1485

TLR6BT GAACTCAACCTTGCTTCCAATTCTTTAGCCCACCTTCCTGGATGTGGTATCTTTAGCAGC 1488

TLR6SS GAACTCAATGTTGCTTCCAATTCTTTAGCTCACCTGCCTGGATGTGGTTCCTTTAGCAGC 1488

TLR6CF GAACTCAATGTTGCTTCCAATTCTTTAGCCCACTTTCCTGACTGTGGTACTTTTAATAGG 1488

* ****** * ** * *** * *** * ** * **** ** ** ** * **

TLR1HS CTTTCTGTATTGATCATTGATCACAATTCAGTTTCCCACCCATCGGCTGATTTCTTCCAG 1533

TLR1PT CTTTCTGTATTGATCATTGATCACAATTCAGTTTCCCACCCATCGGCTGATTTCTTCCAG 1533

TLR1Ma CTTTCTGTATTGATCATTGATCACAATTCAGTTTCCCACCCATCAGCTGATTTCTTCCAG 1533

TLR1MM CTTTCTGTGCTGGTCATCGACCATAACTCAGTTTCCCATCCCTCTGAGGATTTCTTCCAG 1542

TLR1RN CTTTCTGTGCTGGTCATCGACCATAACTCAGTTTCCCACCCCTCCTCTGATTTCTTCCAG 1542

TLR1EE CTTTCTGCACTGATCATTGACTATAACTCAATTTCCAGTCCATCAGTTGATTTCTTCCAG 1545

TLR1BT CTTTCCATACTGATCATTGACTATAACTCAATTTCCAATCCATCAGCTGATTTCTTCCAG 1545

TLR1SS CTTTCCATTCTGATCATTGACTATAATTCAATTTCCAACCCATCAGCTGACTTCTTCCAG 1545

TLR1CF CTTTCTGTACTGATCATTGACTCTAATTCAATTTCCAATCCATCAGCTGATTTCCTCCAG 1545

TLR6HS CTTTCTGTATTGATCATTGATCACAATTCAGTTTCCCACCCATCGGCTGATTTCTTCCAG 1548

TLR6PT CTTTCTGTATTGATCATTGATCACAATTCAGTTTCCCACCCATCAGCTGATTTCTTCCAG 1548

TLR6Ma CTTTCTGTATTGATCATTGATCACAATTCAGTTTCCCACCCATCAGCTGATTTCTTCCAG 1548

TLR6MM CTTTCTGTGCTGGTCATCGACCATAACTCAGTTTCCCATCCCTCTGAGGATTTCTTCCAG 1548

TLR6RN CTTTCTGTGCTGGTCATCGACCATAACTCAGTTTCCCACCCCTCCTCTGATTTCTTCCAG 1548

TLR6EE CTTTCTGCACTGATCATTGACTATAACTCAATTTCCAGTCCATCAGTTGATTTCTTCCAG 1545

TLR6BT CTTTCCATACTGATCATTGACTATAATTCAATTTCCAATCCATCAGCTGATTTCTTCCAG 1548

TLR6SS CTTTCCATTCTGACCATTGACTATAATTCAATTTCCAACCCATCAGCTGACTTCTTCCAG 1548

TLR6CF CTTTCTGTACTGATCATTGACTCTAATTCAATTTCCAATCCATCAGCTGATTTCCTCCAG 1548

***** ** *** ** ** *** ***** ** ** ** *** *****

TLR1HS AGCTGCCAGAAGATGAGGTCAATAAAAGCAGGGGACAATCCATTCCAATGTACCTGTGAG 1593

TLR1PT AGCTGCCAGAAGATGAGGTCAATAAAAGCAGGGGACAATCCATTCCAATGTACCTGTGAG 1593

TLR1Ma AGCTGCCAGAAGATGAGGTCAATAAAAGCAGGAAACAATCCATTCCAGTGTACCTGTGAG 1593

TLR1MM AGCTGTCAGAATATTAGATCCCTAACAGCGGGAAACAACCCATTCCAATGCACATGTGAG 1602

TLR1RN AGCTGTCAGAATATCAGGTCCATAACAGCGGGGAACAACCCATTCCGATGCACATGTGAG 1602

TLR1EE AGCTGCCAGAACATTAGGTCAGTAAAAGCAGGGAACAACCCATTCCAGTGTACATGTGAG 1605

TLR1BT AGCTGCCAGAAGATTAGGTCCCTCAAAGCGGGGAACAATCCATTCCAATGTTCCTGTGAG 1605

TLR1SS AGCTGCCAGAAGATTAGGTCCCTCAAAGCAGGGAACAATCCATTCCAATGTACATGTGAG 1605

TLR1CF AGCTGCCATAACATTAGGTCCATAAGCGCAGGGAATAATCCATTCCAGTGTACATGTGAG 1605

TLR6HS AGCTGCCAGAAGATGAGGTCAATAAAAGCAGGGGACAATCCATTCCAATGTACCTGTGAG 1608

TLR6PT AGCTGCCAGAAGATGAGGTCAATAAAAGCAGGGGACAATCCATTCCAATGTACCTGTGAG 1608

TLR6Ma AGCTGCCAGAAGATGAGGTCAATAAAAGCAGGGAACAATCCATTCCAGTGTACCTGTGAG 1608

TLR6MM AGCTGTCAGAATATTAGATCCCTAACAGCGGGAAACAACCCATTCCAATGCACATGTGAG 1608

TLR6RN AGCTGTCAGAATATCAGGTCCATAACAGCGGGGAACAACCCATTCCGATGCACATGTGAG 1608

TLR6EE AGCTGCCAGAACATTAGGTCAGTAAAAGCAGGGAACAACCCATTCCAGTGTACATGTGAG 1605

TLR6BT AGCTGCCAGAAGATTAGGTCCCTCAAAGTGGGGAACAATCCATTCCAATGTTCCTGTGAG 1608

TLR6SS AGCTGCCAGAAGATTAGGTCCCTCAAAGCAGGGAACAATCCATTCCAATGTACATGTGAG 1608

TLR6CF AGCTGCCATAACATTAGGTCCATGAGCGCAGGGAATAATCCATTCCAATGTACATGTGAG 1608

***** ** ** ** ** ** * * * ** * ** ******* ** * ******

TLR1HS CTAGGAGAATTTGTCAAAAATATAGACCAAGTATCAAGTGAAGTGTTAGAGGGCTGGCCT 1653

TLR1PT CTAAGAGAATTTGTCAAAAATATAGACCAAGTATCAAGTGAAGTGTTAGAGGGCTGGCCT 1653

TLR1Ma CTAAGAGAATTTATCAAAAATATAGAGCAAGTATCAAGTGAAGTGGTAGAGGGCTGGCCT 1653

TLR1MM CTGAGGGACTTTGTCAAGAACATAGGCTGGGTAGCAAGAGAAGTGGTGGAGGGCTGGCCT 1662

TLR1RN CTGAGGGAGTTTGTCAAAAACATAGGTCAGGCATCAAGAGAAGTGGTGGAGGGCTGGCCT 1662

TLR1EE CTCAGAGAGTTTGTCCAGAGAATGGGCCAAGTGTCAAGGGAAGTGGTAGAGGACTGGCCT 1665

TLR1BT CTAAGAGACTTCATCCAAAGTATAGGCCAAGTATCAAGTGACGTGGTAGAGGGCTGGCCT 1665

TLR1SS CTAAGAGACTTCATCCAAAGTCTAGGTCAAGTATCGAGTGACGTGGTAGAGAGTTGGCCT 1665

TLR1CF CTGAGAGAATTTGTCCAAAGTCTAGGCCAGGTAGCAAGCAAAGTAGTAGAGGGTTGGCCT 1665

TLR6HS CTAAGAGAATTTGTCAAAAATATAGACCAAGTATCAAGTGAAGTGTTAGAGGGCTGGCCT 1668

TLR6PT CTAAGACAATTTGTCAAAAGTATAGACCAAGTATCAAGTGAAGTGTTAGAGGGCTGGCCA 1668

TLR6Ma CTAAGAGAATTTGTCAAAAATATAGAGCAAGTATCAAGTGAAGTGGTAGAGGGCTGGCCT 1668

TLR6MM CTGAGGGACTTTGTCAAGAACATAGGCTGGGTAGCAAGAGAAGTGGTGGAGGGCTGGCCT 1668

TLR6RN CTGAGGGAGTTTGTCAAAAACATAGGTCAGGCATCAAGAGAAGTGGTGGAGGGCTGGCCT 1668

TLR6EE CTCAGAGAGTTTGTCCAGAGAATGGGCCAAGTGTCAAGGGAAGTGGTAGAGAACTGGCCT 1665

TLR6BT CTAAGAGACTTTATCCAAAGTGTAGGCCAAGTATCCAGTGACGTGGTAGAGGGCTGGCCT 1668

TLR6SS CTAAGAGACTTCATCCAAAGTCTAGGTCAAGTATCGAGTGACGTGGTAGAGAGTTGGCCT 1668

TLR6CF CTGAGAGAATTTGTCCAAAGTCTAGGCCAGGTAGCAAGCAAAGTAGTAGAGGGTTGGCCT 1668

** * * ** ** * * * * * * ** * ** * *** *****

TLR1HS GATTCTTATAAGTGTGACTACCCGGAAAGTTATAGAGGAACCCTACTAAAGGACTTTCAC 1713

TLR1PT GATTCTTATAAGTGTGACTACCCAGAAAGTTATAGAGGAAGCCCACTAAAGGACTTTCAC 1713

TLR1Ma GATTCTTATAAGTGTGACTACCCAGAAAGTTATAGAGGAACCCCACTAAAGGACTTTCAC 1713

TLR1MM GACTCTTACAGGTGTGACTACCCAGAAAGCTCTAGGGGAACTGCACTGAGGGACTTCCAC 1722

TLR1RN GACTCTTACAGGTGTGATTACCCAGACAGCATTAAGGGAACCCCACTGCAGGACTTCCAC 1722

TLR1EE GGTTCTTACCAGTGTGACTATCCAGAAAGCTTTAAGGGAACTGCACTAAAGGACTTCCAC 1725

TLR1BT GAGTCTTATAAGTGTGACTATCCGGAAAGCTACAAGGGAACCCCTCTAAAGGACTTCCAG 1725

TLR1SS GATTCTTACGAGTGTGAGTATCCAGAAAGCTATAAGGGGACTCTGCTCAAGGACTTCCGT 1725

TLR1CF GATTCTTATAAGTGTGACTCTCCAGAAAACTATAAGGGAACCCTACTGAAGGACTTTCAC 1725

TLR6HS GATTCTTATAAGTGTGACTACCCAGAAAGTTATAGAGGAAGCCCACTAAAGGACTTTCAC 1728

TLR6PT GATTCTTATAAGTGTGACTACCCAGAAAGTTATAGAGGAACCCCACTAAAGGACTTTCAC 1728

TLR6Ma GATTCTTATAAGTGTGACTACCCAGAAAGTTATAGAGGAACCCCACTAAAGGACTTTCAC 1728

TLR6MM GACTCTTACAGGTGTGACTACCCAGAAAGCTCTAAGGGAACTGCACTGAGGGACTTCCAC 1728

TLR6RN GACTCTTACAGATGTGATTACCCAGACAGCATTAAGGGAACCCCACTGCAGGACTTCCAC 1728

TLR6EE GGTTCTTACCAGTGTGACTATCCAGAAAGCTTTAAGGGAACTGCACTAAAGGACTTCCAC 1725

TLR6BT GAGTCTTATAAGTGTGACTATCCGGAAAGCTACAAGGGAACCCCTCTAAAGGACTTCCAG 1728

TLR6SS GATTCTTACGAGTGTGAGTATCCAGAAAGTTATAAGGGGACTCTGCTCAAGGACTTCCGT 1728

TLR6CF GATTCTTATAAGTGTGACTCTCCAGAAAACTATAAGGGAACCCTACTGAAGGACTTTCAC 1728

* ***** ***** * ** ** * * ** * ** ****** *

TLR1HS ATGTCTGAATTATCCTGCAACATAACTCTGCTGATCGTCACCATCGTTGCCACCATGCTG 1773

TLR1PT ATGTCTGAATTATCCTGCAACATAACTCTGCTGATCGTCACCATCGTTGCCACCATGCTG 1773

TLR1Ma ATGTCTGAATTATCCTGCAACATAACTCTGCTGATCGTCACCATCGGTGCCACCATGCTG 1773

TLR1MM ATGTCTCCACTATCCTGTGATACTGTTCTGCTGACTGTCACCATCGGGGCCACTATGCTG 1782

TLR1RN ATGTCTCCACTGTCCTGCGATACAATTCTACTGACTGTCACCATTGGGGCCACTCTGCTG 1782

TLR1EE ATGTCTCAGCTGTCCTGCAACACCACTCTGTTGATTGTCACCATTGTGGTCATTGTGCTG 1785

TLR1BT GTATCTGAGCTATCCTGCAACACAGCTCTGCTGATCGTCACCATTGTGGTCCCTGGGCTG 1785

TLR1SS GTATCTGAATTATCCTGCAACACAGCTCTGCTGATTGTCACCATCGGAGTCACTGGGCTG 1785

TLR1CF GTGTCTCCGTTATCCTGCAACACAACTCTGCTGCTTGTCACCATTGGGGTCGCTGTGCTA 1785

TLR6HS ATGTCTGAATTATCCTGCAACATAACTCTGCTGATCGTCACCATCGGTGCCACCATGCTG 1788

TLR6PT ATGTCTGAATTATCCTGCAACATAACTCTGCTGATCATCACCATCGGTGCCACCATGCTG 1788

TLR6Ma ATGTCTGAATTATCCTGCAACATAACTCTGCTGATCATCACCATCGGTGCCACCATGCTG 1788

TLR6MM ATGTCTCCACTGTCCTGTGATACTGTTCTGCTGACTGTCACCATCGGGGCCACTATGCTG 1788

TLR6RN ATGTCTCCACTGTCCTGCGATACAATTCTACTGACTGTCACCATTGGGGCCACTCTGCTG 1788

TLR6EE ATGTCTCAGCTGTCCTGCAACACCACTCTGTTGATTGTCACCATTGTGGTCATTGTGCTG 1785

TLR6BT GTATCTGAGCTATCCTGCAACACAGCTCTGCTGATCGTCACCATTGTGGTCCCTGGGCTG 1788

TLR6SS GTATCTGAATTATCCTGCAACACAGCTCTGCTGATTGTCACCATCGGAGTCACTGGGCTG 1788

TLR6CF GTGTCTCCGTTATCCTGCAACACAACTCTGCTGCTTGTCACCATTGGGGTCGCTGTGCTA 1788

* *** * ***** * * *** ** ******* * * * ***

TLR1HS GTGTTGGCTGTGACTGTGACCTCCCTCTGCAGCTACTTGGATCTGCCCTGGTATCTCAGG 1833

TLR1PT GTGTTGGCTGTGACTGTGACCTCCCTCTGCATCTACTTGGATCTGCCCTGGTATCTCAGG 1833

TLR1Ma GTGTTGGCTGTGACTGTGACCTTCCTCTGCATCTACTTGGATCTGCCCTGGTATCTCAGG 1833

TLR1MM GTGCTGGCTGTCACTGGGGCTTTCCTCTGTCTCTACTTTGACCTGCCCTGGTATGTGAGG 1842

TLR1RN CTGCTGGCTGCCATTGGGGCTTCCCTCTGTCTCTACTTTGATCTGCCCTGGTATCTCAGG 1842

TLR1EE GTGTTGGGTAGTACCACGGTCATGCTCTGTATCTACTTTGATGTGCTCTGGTATCTGAGG 1845

TLR1BT GTGCTGGCTGTTGCTGTGACTGTCCTCTGTATCTACCTGGATCTGCCCTGGTACCTCAGG 1845

TLR1SS GCACTGGCTCTTACCATGACCGGCCTCTGTGTCTACTTTGATCTGCCCTGGTATCTCAGG 1845

TLR1CF GTGTTCACTGTTACTGTGACTGCGCTCTGTATCTACTTTGATCTGCCCTGGTATCTTAGG 1845

TLR6HS GTGTTGGCTGTGACTGTGACCTCCCTCTGCATCTACTTGGATCTGCCCTGGTATCTCAGG 1848

TLR6PT GTGTTGGCTGTGACTGTGACCTCCCTCTGCATCTACTTGGATCTGCCCTGGTATCTCAGG 1848

TLR6Ma GTGTTGGCTGTGACTGTGACCTTCCTCTGCATCTACTTGGATCTGCCCTGGTATCTCAGG 1848

TLR6MM GTGCTGGCTGTCACTGGGGCTTTCCTCTGTCTCTACTTTGACCTGCCCTGGTATGTGAGG 1848

TLR6RN CTACTGGCTGCCATTGGGGCTTCCCTCTGTCTCTACTTTGATCTGCCCTGGTATCTCAGG 1848

TLR6EE GTGTTGGGTAGTACCACGGTCATGCTCTGTATCTACTTTGATGTGCTCTGGTATCTGAGG 1845

TLR6BT GTGCTGGCTGTTGCTGTGACTGTCCTCTGTATCTACCTGGATCTGCCCTGGTACCTCAGG 1848

TLR6SS GCATTGGCTCTTACCATGACCGGCCTCTGTGTCTACTTTGATCTGCCCTGGTATCTCAGG 1848

TLR6CF GTGTTCACTGTTACTGTGACTGCGCTCTGTATCTACTTTGATCTGCCCTGGTATCTTAGG 1848

* * * ***** **** * ** *** ****** * ***

TLR1HS ATGGTGTGCCAGTGGACCCAGACCCGGCGCAGGGCCAGGAACATACCCTTAGAAGAACTC 1893

TLR1PT ATGGTGTGCCAGTGGACCCAGACCCGGCGCAGGGCCAGGAACATACCCTTAGAAGAACTC 1893

TLR1Ma ATGGTGTGCCAGTGGACCCAGACCCGGCGCAGGGCCAGGAATGTACCCTTAGAAGAACTC 1893

TLR1MM ATGCTGTGTCAGTGGACACAGACCAGGCACAGGGCCAGGCACATCCCCTTAGAGGAACTC 1902

TLR1RN ATGCTATGGCAGTGGACACAGACCAGGCACAGGGCCCGGAACATCCCCTTAGAGGAACTG 1902

TLR1EE ATGATGTGCCATTGGACCCAGACCCGGCAAAGGGCTAGGAACACTCCCTTAGCTGAACTC 1905

TLR1BT ATGGTGTGTCAGTGGACCCAGACCCGGCGCAGGGCCAGGAATGTACCCTTGGAAGAACTC 1905

TLR1SS ATGCTGTGTCAGTGGACCCAGACTCGGCGCAGGGCTAGGAATGTACCCTTAGAAGAACTC 1905

TLR1CF ATGGTGTTTCAGTGGACCCAGACCCGGCGCAGGGCAAGAAACACACCCTTAGAAAATCTC 1905

TLR6HS ATGGTGTGCCAGTGGACCCAGACTCGGCGCAGGGCCAGGAACATACCCTTAGAAGAACTC 1908

TLR6PT ATGGTGTGCCAGTGGACCCAGACCCGGCGCAGGGCCAGGAACATACCCTTAGAAGAACTC 1908

TLR6Ma ATGGTGTGCCAGTGGACCCAGACCCGGCACAGGGCCAGGAATGTACCCTTAGAAGAACTC 1908

TLR6MM ATGCTGTGTCAGTGGACACAGACCAGGCACAGGGCCAGGCACATCCCCTTAGAGGAACTC 1908

TLR6RN ATGCTATGGCAGTGGACACAGACCAGGCACAGGGCCAGGAACATCCCCTTAGAGGAACTG 1908

TLR6EE ATGATGTGCCATTGGACCCAGACCCGGCAAAGGGCTAGGAACACTCCCTTAGCTGAACTC 1905

TLR6BT ATGGTGTGTCAGTGGACCCAGACCCGGCGCAGGGCCAGGAATGTACCCTTGGAAGAACTC 1908

TLR6SS ATGCTGTGTCAGTGGACCCAGACTCGGCGCAGGGCTAGGAATGTACCCTTAGAAGAACTC 1908

TLR6CF ATGGTGTTTCAGTGGACCCAGACCCGGCGCAGGGCAAGAAACACACCCTTAGAAGAACTC 1908

*** * * ** ***** ***** *** ***** * * ***** * * **

TLR1HS CAAAGAAATCTCCAGTTTCATGCATTTATTTCATATAGTGGGCACGATTCTTTCTGGGTG 1953

TLR1PT CAAAGAAATCTCCAGTTTCATGCATTTATTTCATATAGTGGGCACGATTCTTTCTGGGTG 1953

TLR1Ma CAAAGAAATCTCCAGTTTCATGCATTTATTTCATATAGTGGGCACGATTCTTTCTGGGTG 1953

TLR1MM CAGAGAAACCTCCAGTTCCATGCTTTTGTCTCATACAGTGGGCATGATTCTGCCTGGGTG 1962

TLR1RN CAGAGGAACCTCCAGTTCCATGCTTTTGTCTCATACAGTGGGCATGATTCTGCCTGGGTG 1962

TLR1EE CAGAGAAACCTCCAGTTCCATGCTTTCATTTCATATAGTGAACATGATTCCGCCTGGGTG 1965

TLR1BT CAAAGAACTCTCCAGTTCCATGCTTTTATTTCATATAGTGGGCACGATTCTGCCTGGGTG 1965

TLR1SS CAAAGAACTCTCCAGTTCCATGCCTTCATTTCATATAGTGGGCATGATTCTGCTTGGGTA 1965

TLR1CF CAAAGAACCATCCAGTTCCATGCTTTTATTTCATATAGCGGGCATGATTCTGCCTGGGTG 1965

TLR6HS CAAAGAAACCTCCAGTTTCATGCTTTTATTTCATATAGTGAACATGATTCTGCCTGGGTG 1968

TLR6PT CAAAGAAATCTCCAGTTTCATGCTTTTATTTCATATAGTGAACATGATTCTGCCTGGGTG 1968

TLR6Ma CAAAGAAATCTCCAGTTTCATGCATTTATTTCATATAGTGAACATGATTCTGCCTGGGTG 1968

TLR6MM CAGAGAAACCTCCAGTTCCATGCTTTTGTCTCATACAGTGAGCATGATTCTGCCTGGGTG 1968

TLR6RN CAGAGGAACCTCCAGTTCCATGCTTTTGTCTCATACAGTGAGCATGATTCTGCCTGGGTG 1968

TLR6EE CAGAGAAACCTCCAGTTCCATGCTTTCATTTCATATAGTGAACATGATTCCGCCTGGGTG 1965

TLR6BT CAAAGAACTCTCCAGTTCCATGCTTTTATTTCATACAGTGAACATGACTCTGCCTGGGTG 1968

TLR6SS CAAAGAACTCTCCAGTTCCATGCCTTCATTTCATATAGTGAACACGATTCTGCCTGGGTA 1968

TLR6CF CAAAGAACCATCCAGTTCCATGCTTTTATTTCATACAGTGAACATGATTCTGCCTGGGTG 1968

** ** * ******* ***** ** * ***** ** * ** ** ** *****

TLR1HS AAGAATGAATTATTGCCAAACCTAGAGAAAGAAGGTATGCAGATTTGCCTTCATGAGAGA 2013

TLR1PT AAGAATGAATTATTACCAAACCTAGAGAAAGAAGGTATGCAGATTTGCCTTCATGAGAGA 2013

TLR1Ma AAGAATGAATTATTACCAAACCTAGAGAAAGAAGGTATGCAGATTTGCCTTCATGAGAGA 2013

TLR1MM AAGAACGAATTACTACCCAACCTAGAGAAAGATGACATCCAGATTTGCCTCCATGAGAGG 2022

TLR1RN AAGAATGAATTACTACCAAACCTAGAGAAAGATGACATTCGGGTTTGCCTCCATGAGAGA 2022

TLR1EE AAGAGTGAATTACTACCAAACTTAGAAAAAGAAAATATACGAATTTGTCTTCATGAGAGA 2025

TLR1BT AAGAATGAATTAATACCTAACCTAGAAAAAGAAGATATAAGAATTTGTCTCCATGAGAGA 2025

TLR1SS AAGAATGAATTACTACCAAATGTAGAAAAAGAAGGTATAAAGATTTGTCTCCATGAGAGA 2025

TLR1CF AAGAGTGAATTACTACCAAACCTAGAAAAAGAAGAACTAAGGATTTGTCTCCATGAGAGA 2025

TLR6HS AAAAGTGAATTGGTACCTTACCTAGAAAAAGAAGATATACAGATTTGTCTTCATGAGAGA 2028

TLR6PT AAAACTGAATTGGTACCTTACCTAGAAAAAGAAGATATACAGATTTGTCTTCATGAAAGA 2028

TLR6Ma AAAAATGAATTGGTACCTTACCTAGAGAAAGAAGGTATGCAGGTTTGCCTTCATGAGAGA 2028

TLR6MM AAGAACGAATTACTACCCAACCTAGAGAAAGATGACATCCGGGTTTGCCTCCATGAGAGG 2028

TLR6RN AAGAATGAATTACTACCAAACCTAGAGAAAGATGACATTCGGGTTTGCCTCCATGAGAGA 2028

TLR6EE AAGAATGAGCTGGTACCCTGCCTAGAAAAAGAAAATATACGAATTTGTCTTCATGAGAGA 2025

TLR6BT AAGAATGAATTAATACCTAACCTAGAAAAAGAAGATATAAGAATTTGTCTCCACGAGAGA 2028

TLR6SS AAAAATGAACTGGTACCTTGTCTAGAAAAAGAAGGTATAAAGATTTGTCTCCATGAGAGA 2028

TLR6CF AAGAATGAACTGGTACCCTGCCTAGAAAAAGAAGAACTAAGGATTTGTCTCCATGAGAGA 2028

** * ** * * ** **** ***** * **** ** ** ** **

TLR1HS AACTTTGTTCCTGGCAAGAGCATTGTGGAAAATATCATCACCTGCATTGAGAAGAGTTAC 2073

TLR1PT AACTTTGTTCCTGGCAAGAGCATTGTGGAAAATATCATCACCTGCATTGAGAAGAGTTAC 2073

TLR1Ma AACTTTGTTCCTGGCAAGAGCATTGTGGAAAATATCATCAACTGCATTGAGAAGAGTTAC 2073

TLR1MM AACTTTGTCCCTGGCAAGAGCATTGTGGAGAACATCATCAATTTCATTGAGAAGAGTTAC 2082

TLR1RN AACTTTGTCCCTGGCAAGAGCATTGTGGAGAACATCATACACTTCATTGAGAAGAGTTAC 2082

TLR1EE AACTTTGTCCCTGGGAAGAGCATCATAGAAAACATTATCAACTGCATTGAGAAAAGTTAC 2085

TLR1BT AACTTTGTTGCTGGCAAGAGCATTGTGGAAAATATCATCAACTGCATTGAGAAAAGTTAC 2085

TLR1SS AACTTTGTTCCTGGCAAGAGCATCATGGAAAATATCATAAACTGCATTGAGAAAAGCTAC 2085

TLR1CF AACTTTATTCCTGGCAAGAGCATTGTGGAAAATATCATAAACTGCATTGAGAAAAGTTAC 2085

TLR6HS AACTTTGTCCCTGGCAAGAGCATTGTGGAAAATATCATCAACTGCATTGAGAAGAGTTAC 2088

TLR6PT AACTTTGTCCCTGGCAAGAGCATTGTGGAAAATATCATCAACTGCATTGAGAAGAGTTAC 2088

TLR6Ma AACTTTGTTCCTGGTAAGAGCATTGTGGAAAATATCATCAACTGCATTGAGAAGAGTTAC 2088

TLR6MM AACTTTGTCCCTGGCAAGAGCATTGTGGAGAACATCATCAATTTCATTGAGAAGAGTTAC 2088

TLR6RN AACTTTGTCCCTGGCAAGAGCATTGTGGAGAACATCATACACTTCATTGAGAAGAGTTAC 2088

TLR6EE AACTTTGTCCCTGGGAAGAGCATCATAGAAAACATTATCAACTGCATTGAGAAAAGTTAC 2085

TLR6BT AACTTTGTTGCCGGCAAGAGCATTGTGGAAAATATCATCAACTGCATTGAGAAAAGTTAC 2088

TLR6SS AACTTTGTTCCTGGCAAGAGCATCATGGAAAATATCATAAACTGCATTGAGAAAAGCTAC 2088

TLR6CF AACTTTATTCCTGGCAAGAGCATTGTGGAAAATATCATAAACTGCATTGAGAAAAGTTAC 2088

****** * * ** ******** * ** ** ** ** * ********* ** ***

TLR1HS AAGTCCATCTTTGTTTTGTCTCCCAACTTTGTCCAGAGTGAATGGTGCCATTATGAACTC 2133

TLR1PT AAGTCCATCTTTGTTTTGTCTCCCAACTTTGTCCAGAGTGAATGGTGCCATTATGAACTC 2133

TLR1Ma AAGTCCATCTTTGTTTTGTCTCCCAACTTTGTCCAGAGTGAGTGGTGCCATTATGAACTC 2133

TLR1MM AAGTCCATCTTTGTGCTGTCTCCCCACTTCATCCAGAGTGAGTGGTGTCATTATGAACTC 2142

TLR1RN AAGTCCATCTTTGTGCTGTCTCCCCACTTCATCCAGAGTGAGTGGTGCCATTATGAACTC 2142

TLR1EE AAGTCCATCTTTATTCTGTCTCCCAACTTTGTACAGAGTGAGTGGTGCCATTATGAGCTC 2145

TLR1BT AAATCCATCTTTGTCTTGTCTCCCAACTTTGTCCAGAGCGAATGGTGCCATTATGAACTC 2145

TLR1SS AAGTCCATCTTTGTTTTGTCTCCCAACTTTGTCCAGAGCGAGTGGTGCCACTATGAACTC 2145

TLR1CF AAGTCCATCTTTGTTCTGTCTCCCAACTTTGTTCAGAGTGAGTGGTGCCATTATGAACTG 2145

TLR6HS AAGTCCATCTTTGTTTTGTCTCCCAACTTTGTCCAGAGTGAGTGGTGCCATTACGAACTC 2148

TLR6PT AAGTCCATCTTTGTTTTGTCTCCCAACTTTGTCCAGAGTGAATGGTGCCATTACGAACTC 2148

TLR6Ma AAGTCCATCTTTGTTTTGTCTCCCAACTTTGTCCAGAGTGAGTGGTGCCATTATGAACTC 2148

TLR6MM AAGGCCATCTTTGTGCTGTCTCCCCACTTCATCCAGAGTGAGTGGTGCCATTATGAACTC 2148

TLR6RN AAGTCCATCTTTGTGCTGTCTCCCCACTTCATCCAGAGTGAGTGGTGCCATTATGAACTC 2148

TLR6EE AAGTCCATCTTTGTTCTGTCTCCCAACTTTGTACAGAGTGAGTGGTGCCATTATGAGCTC 2145

TLR6BT AAATCCATCTTTGTCTTGTCTCCCAACTTTGTCCAGAGCGAATGGTGCCATTATGAACTC 2148

TLR6SS AAGTCCATCTTTGTTTTGTCTCCCAACTTTGTCCAGAGCGAGTGGTGCCACTATGAACTC 2148

TLR6CF AAGTCCATCTTTGTTCTGTCTCCCAACTTTGTTCAGAGTGAGTGGTGCCATTATGAACTG 2148

** ******** * ******** **** * ***** ** ***** ** ** ** **

TLR1HS TACTTTGCCCATCACAATCTCTTTCATGAAGGATCTAATAGCTTAATCCTGATCTTGCTG 2193

TLR1PT TACTTTGCCCATCACAATCTCTTTCATGAAGGATCTAATAACTTAATCCTGATCTTGCTG 2193

TLR1Ma TACTTTGCCCATCACAATCTCTTTCATGAAGGATCTAATAACTTAATCCTGATCTTGCTG 2193

TLR1MM TATTTTGCCCATCACAATCTCTTCCATGAAGGCTCTGATAACTTAATCCTCATCTTGCTG 2202

TLR1RN TACTTTGCCCATCACAATCTCTTCCACGAAGGGTCTGATAACTTAATCCTGATCTTGCTG 2202

TLR1EE TACTTTGCCCACCACAATCTCTTTCATAAAGGGTCTGATAACTTAATCTTGATCTTGCTA 2205

TLR1BT TACTTTGCCCACCACAATCTCTTCCATGAAGGATCTGATAACTTAATCCTGATCTTGCTG 2205

TLR1SS TACTTTGCCCACCACAACCTCTTCCATGAAGGGTCTGATAACTTAATCCTGATCTTGCTG 2205

TLR1CF TACTTTGCCCACCACAATCTCTTTCATGAAGGATCTAATAACTTAATCTTGATCTTGCTG 2205

TLR6HS TATTTTGCCCATCACAATCTCTTTCATGAAGGATCTAATAACTTAATCCTCATCTTACTG 2208

TLR6PT TATTTTGCCCATCACAATCTCTTTCATGAAGGATCTAATAACTTAATCCTCATCTTACTG 2208

TLR6Ma TACTTTGCCCATCACAATCTCTTTCATGAAGGATCTAATAACCTAATCCTCATCTTACTG 2208

TLR6MM TATTTTGCCCATCATAATCTCTTCCATGAAGGCTCTGATAACTTAATCCTCATCTTGCTG 2208

TLR6RN TACTTTGCCCATCACAATCTCTTCCACGAAGGGTCTGATAACTTAATCCTGATCTTGCTG 2208

TLR6EE TACTTTGCCCACCACAATCTCTTTCATGAAGGGTCTGATAACTTAATCTTGATCTTGCTG 2205

TLR6BT TACTTTGCCCACCACAATCTCTTCCATGAAGGATCTAATAACTTAATCCTGATCTTGCTG 2208

TLR6SS TACTTTGCCCACCACAACCTCTTCCATGAAGGGTCTGATAACTTAATCCTGATCTTGCTG 2208

TLR6CF TACTTTGCCCACCACAATCTCTTTCATGAAGGATCTAATAACTTAATCTTGATCTTGCTG 2208

** ******** ** ** ***** ** **** *** *** * ***** * ***** **

TLR1HS GAACCCATTCCGCAGTACTCCATTCCTAGCAGTTATCACAAGCTCAAAAGTCTCATGGCC 2253

TLR1PT GAACCCATTCCACAGTACTCCATTCCTAGCAGTTATCACAAGCTCAAAAGTCTCATGGCC 2253

TLR1Ma GAACCCATTCCGCAGTACTCCATTCCTAGCAGCTATCACAAGCTCAAAAATCTCATGGCC 2253

TLR1MM GCACCCATTCCCCAGTACTCCATCCCTACCAATTACCACAAGCTCAAAACTCTCATGTCA 2262

TLR1RN GAACCAATTCCACAGTACTCCATCCCTACCAATTACCACAAGCTCAAAACTCTCATGGCA 2262

TLR1EE GAACCCATTCCACAGTATTCCATTCCTAACAGTTATCACAAGCTTAAAGCTCTCATGGCA 2265

TLR1BT GATCCCATTCCACAGTATTCCATTCCTAGCAGCTACCACAAGCTAAGAGCTCTCATGGCA 2265

TLR1SS GATTCCATTCCACAGTATTCCATCCCCAGCAGCTATCACAAACTCAAAGCTCTCATGGCA 2265

TLR1CF GAACCTATTCCACAGTATTCCATTCCTAGCAGCTATCACAAGCTCAAAAATCTCATGGCA 2265

TLR6HS GAACCCATTCCACAGAACAGCATTCCCAACAAGTACCACAAGCTGAAGGCTCTCATGACG 2268

TLR6PT GAACCCATTCCACAGAACAGCATTCCCAACAAGTACCACAAGCTGAAGGCTCTCATGATG 2268

TLR6Ma GAACCCATTCCACAGAATAGCATTCCCAACAAGTACCACAAGCTGAGGGCTCTGATGACT 2268

TLR6MM GAACCCATTCTACAGAACAACATTCCCAGTAGATACCACAAGCTGCGGGCTCTCATGGCA 2268

TLR6RN GAACCCATCCAACAGAACAACATTCCCAGTAGATACCACAAGCTGAGGGCTCTCATGGCA 2268

TLR6EE GAACCCATTCCACAGAACAACATTCCTAGTAAGTATCACAAGCTGAAGGCTCTCATGACA 2265

TLR6BT GAACCCATTCCACAGAACACCATTCCTGATAGATATCACAAGCTAAGAGCTCTCATGGCA 2268

TLR6SS GATCCCATTCCACAGAACAGCATTCCTGGCAAGTATCACAAACTCAAAGCTCTCATGGCA 2268

TLR6CF GAACCCATTCCACAGAACTGCATTCCCAGCAAGTATCACAAGCTGAGGGCTCTCATGACG 2268

* * ** * *** * *** ** * ** ***** ** *** ***

TLR1HS AGGAGGACTTATTTGGAATGGCCCAAGGAAAAGAGCAAACGTGGCCTTTTTTGGGCTAAC 2313

TLR1PT AGGAGGACTTATTTGGAATGGCCCAAGGAAAAGAGCAAACGTGGCCTTTTTTGGGCTAAC 2313

TLR1Ma AGGAGGACTTATTTGGAATGGCCCAAGGAAAAGAGCAAACATGGGCTTTTTTGGGCTAAT 2313

TLR1MM CGAAGGACCTATCTGGAATGGCCCACAGAGAAGAACAAGCATGGACTTTTTTGGGCAAAC 2322

TLR1RN CGGAGGACCTATTTGGAATGGCCCACAGAAAAGAGCAAGCATGGACTCTTTTGGGCAAAT 2322

TLR1EE AGAAGGACTTATTTGGAATGGCCCAAGGAGAAGAGAAAACATGGACTTTTTTGGGCTAGC 2325

TLR1BT CAGAGAACTTATTTGGAATGGCCCAAGGAGAAGAGTAAACACGGACTTTTTTGGGCTAAC 2325

TLR1SS CAGCGAACTTATTTGGAATGGCCCAAGGAGAAGAGCAAACATGGACTTTTTTGGGCTAAT 2325

TLR1CF CAAAGGACTTATTTGGAATGGCCCAAGGAGAAGAGCAAACATGGACTTTTTTGGGCTAAC 2325

TLR6HS CAGCGGACTTATTTGCAGTGGCCCAAGGAGAAAAGCAAACGTGGGCTCTTTTGGGCTAAC 2328

TLR6PT CAGCGGACTTATTTGCAGTGGCCCAAGGAGAAAAGCAAACGTGGGCTCTTTTGGGCTAAC 2328

TLR6Ma CAGAGGACTTATTTGCAATGGCCCAAGGAGAAAAGCAAACGTGGGCTCTTTTGGGCTAAC 2328

TLR6MM CAGCGGACTTACTTGGAATGGCCTACTGAGAAGGGCAAACGTGGGCTGTTTTGGGCCAAC 2328

TLR6RN CAGCGGACTTACTTGGAATGGCCTATTGAGAAGGGTAAACGTGGGCTGTTTTGGGCCAAC 2328

TLR6EE CAACGAACTTATTTGGAATGGCCTAAGGAGAAGAGCAAACATGGACTTTTCTGGGCTAAC 2325

TLR6BT CAGAGAACTTATTTGGAATGGCCCAAGGAGAAGAACAAGCATGGACTCTTTTGGGCTAAT 2328

TLR6SS CAGCGAACTTATTTGGAATGGCCCAAGGAGAAGAGCAAACATGGACTTTTTTGGGCTAAT 2328

TLR6CF CAGCGGACTTACTTGGAATGGCCCAAGGAGAAGAGCAAACATGGACTTTTTTGGGCTAAT 2328

* ** ** ** * ***** * ** ** ** * ** ** ** ***** *

TLR1HS TTAAGGGCAGCCATTAATATTAAGCTGACAGAGCAAGCAAAGAAATAG------------ 2361

TLR1PT TTAAAGGCAGCCATTAATATTAAGCTGACAGAGCAAGCAAAGAAATAG------------ 2361

TLR1Ma CTAAGGGCAGCCATTAATATTAAGCTGACAGAGCAAGCAAAAAAATAG------------ 2361

TLR1MM CTAAGAGCATCCATTAATGTTAAGCTGGTTAACCAGGCAGAAGGAACGTGTTACACACAG 2382

TLR1RN CTAAGAGCATCCATTAATGTTAAGCTGGTCAACCAGGCAGAAGCAACATGTTACACACAG 2382

TLR1EE TTAAGAGTATCCATTAATATTAAATTGACAGAGCAAGCAAAAGAAGTATGTCATACACAA 2385

TLR1BT CTAAGAGCATCCATTAATATTAAACTGATGGAAAAAGCAGCAGAAATACATTAA------ 2379

TLR1SS CTGAGAGCATCCATTAATATTAAATTGATGGAGAAAGCAGAAGAAATAAGTTACACACAG 2385

TLR1CF CTAAGAGCGTCTATTAATATTAAATTGAGGGAGCAAGCAAAAAAATAG------------ 2373

TLR6HS ATTAGAGCCGCTTTTAATATGAAATTAACA---CTAGTCACTGAAAACAATGATGTGAAA 2385

TLR6PT ATTAGAGCCGCTTTTAATATGAAATTAACA---CTAGTCACTGAAAACAATGATGTGAAA 2385

TLR6Ma ATTAGAGCCACTTTTAATGTGAAATTAACA---CTAGTCACTGAAAACAATGATGTGAAA 2385

TLR6MM CTTAGAGCTTCATTTATTATGAAGTTAGCC---TTAGTCAATGAGGA---TGATGTGAAA 2382

TLR6RN CTTAGAGCTTCTTTTATTATGAAGCTAGCC---TTAGTTAATGAGAA---TGATGTGAAA 2382

TLR6EE ATCACAGCTGCTTTTCATATGAAATTAACA---CTAGTGAATGAAAA---TGATGCAGAA 2379

TLR6BT ATTAGAGCTGCTTTTAATATTAAATTAAGA---CTAGTCACTGAAAATGATGATGTGAAA 2385

TLR6SS ATTAGAGCTGCTTTCAATATTAAATTAAAA---CTAGTCGCTGAAGAGGATGATGTGAAA 2385

TLR6CF ATTAGAGCTGCTTTTAATATGAAGTTAACA---CTAATTGCTGAAAACAATAACGCAGAA 2385

* * * * * * * ** *

TLR1HS ---------------------------------

TLR1PT ---------------------------------

TLR1Ma ---------------------------------

TLR1MM CAATAA--------------------------- 2388

TLR1RN CAATAA--------------------------- 2388

TLR1EE ATCCAAAATATTCTTACTACTTCTGCTTTTTGA 2418

TLR1BT ---------------------------------

TLR1SS ATCTAA--------------------------- 2391

TLR1CF ---------------------------------

TLR6HS TCTTAA--------------------------- 2391

TLR6PT TCTTAA--------------------------- 2391

TLR6Ma TCTTAA--------------------------- 2391

TLR6MM ACTTGA--------------------------- 2388

TLR6RN ACTTGA--------------------------- 2388

TLR6EE ACTTAG--------------------------- 2385

TLR6BT GGTTAA--------------------------- 2391

TLR6SS ACTTAA--------------------------- 2391

TLR6CF GCTTCTTAA------------------------ 2394

**TLR1 and TLR6 protein sequences**

>TLR1HS

MTSIFHFAII FMLILQIRIQ LSEESEFLVD RSKNGLIHVP KDLSQKTTIL NISQNYISEL

WTSDILSLSK LRILIISHNR IQYLDISVFK FNQELEYLDL SHNKLVKISC HPTVNLKHLD

LSFNAFDALP ICKEFGNMSQ LKFLGLSTTH LEKSSVLPIA HLNISKVLLV LGETYGEKED

PEGLQDFNTE SLHIVFPTNK EFHFILDVSV KTVANLELSN IKCVLEDNKC SYFLSILAKL

QTNPKLSSLT LNNIETTWNS FIRILQLVWH TTVWYFSISN VKLQGQLDFR DFDYSGTSLK

ALSIHQVVSD VFGFPQSYIY EIFSNMNIKN FTVSGTRMVH MLCPSKISPF LHLDFSNNLL

TDTVFENCGH LTELETLILQ MNQLKELSKI AEMTTQMKSL QQLDISQNSV SYDEKKGDCS

WTKSLLSLNM SSNILTDTIF RCLPPRIKVL DLHSNKIKSI PKQVVKLEAL QELNVAFNSL

TDLPGCGSFS SLSVLIIDHN SVSHPSADFF QSCQKMRSIK AGDNPFQCTC ELGEFVKNID

QVSSEVLEGW PDSYKCDYPE SYRGTLLKDF HMSELSCNIT LLIVTIVATM LVLAVTVTSL

CIYLDLPWYL RMVCQWTQTR RRARNIPLEE LQRNLQFHAF ISYSGHDSFW VKNELLPNLE

KEGMQICLHE RNFVPGKSIV ENIITCIEKS YKSIFVLSPN FVQSEWCHYE LYFAHHNLFH

EGSNSLILIL LEPIPQYSIP SSYHKLKSLM ARRTYLEWPK EKSKRGLFWA NLRAAINIKL

TEQAKK

>TLR1PT

MPSIFHFAII FMLILQIRIQ LSEESEFLVD RSKNGLIHVP KDLSQKTTIL NISQNYISEL

WTSDILSLSK LRILIISHNR IQYLDISVFK FNHELEYLDL SHNKLVKISC HPTVNLKHLD

LSFNAFDALP ICKEFGNMSQ LKFLGLSTTH LEKSSVLPIA HLNISKILLV LGETYGEKED

PEGLQDFNTE SLHIVFPTNK EFHFILDVSV KTVANLELSN IKCVLEDNKC SYFLSILAKL

QTNPKLSSLT LNNIETTWNS FIRILQLVWH TTVWYFSISN VKLQGQLDFR DFDYSGTSLK

ALSIHQVVSD VFSFPQSDIY EIFSNMNIKN FTVSGTRMVH MLCPSKISPF LHLDFSNNLL

TDTVFENCGH LTELETLILQ MNQLKELSKI AEMTTQMKSL QQLDISQNSV SYDEKKGDCS

WTKSLLSLNM SSNILTDTIF RCLPPRIKVL DLHSNKIKSV PKQVVKLEAL QELNVAFNSL

TDLPGCGSFS SLSVLIIDHN SVSHPSADFF QSCQKMRSIK AGDNPFQCTC ELREFVKNID

QVSSEVLEGW PDSYKCDYPE SYRGSPLKDF HMSELSCNIT LLIVTIVATM LVLAVTVTSL

CIYLDLPWYL RMVCQWTQTR RRARNIPLEE LQRNLQFHAF ISYSGHDSFW VKNELLPNLE

KEGMQICLHE RNFVPGKSIV ENIITCIEKS YKSIFVLSPN FVQSEWCHYE LYFAHHNLFH

EGSNNLILIL LEPIPQYSIP SSYHKLKSLM ARRTYLEWPK EKSKRGLFWA NLKAAINIKL

TEQAKK

>TLR1Ma

MTSIFHFAII FMLTLQIRIQ LSEESEFLVD RSKNSLIHVP KDLSQKTTIL NISQNYISEL

WTSDILSLSK LRILIISHNR LQYLDISVFK FNQELEYLDL SHNKLAKISC HPTVNLKHLD

LSFNAFDALP ICKEFGNMSQ LKFLGLSTTH LEKSTVLPIA HLNISKVLLV LGEHYGDKED

PEGLQNFNTE SLHIVFPTSK EFNFILDVSV RTVANLELSN IKCVLEDNEC SYFLNILAKL

QTNPKLSSLT LNNIETTWNS FIRILQLVWH TTVWYFSISN VKLQGQLDFR DFDYSGTSLK

ALSVHQVVSD VFNFPQRDIY EIFSNMNIKN FTVSGTRMIH MVCPSKISPF LHLDFSNNLL

TDTVFENCGH LTELETLILQ MNQLKELSKI AEMTTRMKSL QQLDISQNSV SYDEKKGDCS

WTKSLLSLNM SSNILTDTIF KCLPPRIKVL DLHSNKIKSI PKQVIKLEAL QELNVAFNSL

TDLPGCGSFS SLSVLIIDHN SVSHPSADFF QSCQKMRSIK AGNNPFQCTC ELREFIKNIE

QVSSEVVEGW PDSYKCDYPE SYRGTPLKDF HMSELSCNIT LLIVTIGATM LVLAVTVTFL

CIYLDLPWYL RMVCQWTQTR RRARNVPLEE LQRNLQFHAF ISYSGHDSFW VKNELLPNLE

KEGMQICLHE RNFVPGKSIV ENIINCIEKS YKSIFVLSPN FVQSEWCHYE LYFAHHNLFH

EGSNNLILIL LEPIPQYSIP SSYHKLKNLM ARRTYLEWPK EKSKHGLFWA NLRAAINIKL

TEQAKK

>TLR1MM

MTKPNSLIFY CIIVLGLTLM KIQLSEECEL IIKRPNANLT RVPKDLPLQT TTLDLSQNNI

SELQTSDILS LSKLRVLIMS YNRLQYLNIS VFKFNTELEY LDLSHNELKV ILCHPTVSLK

HLDLSFNAFD ALPICKEFGN MSQLQFLGLS GSRVQSSSVQ LIAHLNISKV LLVLGDAYGE

KEDPESLRHV STETLHIVFP SKREFRFLLD VSVSTTIGLE LSNIKCVLED QGCSYFLRAL

SKLGKNLKLS NLTLNNVETT WNSFINILQI VWHTPVKYFS ISNVKLQGQL AFRMFNYSDT

SLKALSIHQV VTDVFSFPQS YIYSIFANMN IQNFTMSGTH MVHMLCPSQV SPFLHVDFTD

NLLTDMVFKD CRNLVRLKTL SLQKNQLKNL ENIILTSAKM TSLQKLDISQ NSLRYSDGGI

PCAWTQSLLV LNLSSNMLTG SVFRCLPPKV KVLDLHNNRI MSIPKDVTHL QALQELNVAS

NSLTDLPGCG AFSSLSVLVI DHNSVSHPSE DFFQSCQNIR SLTAGNNPFQ CTCELRDFVK

NIGWVAREVV EGWPDSYRCD YPESSRGTAL RDFHMSPLSC DTVLLTVTIG ATMLVLAVTG

AFLCLYFDLP WYVRMLCQWT QTRHRARHIP LEELQRNLQF HAFVSYSGHD SAWVKNELLP

NLEKDDIQIC LHERNFVPGK SIVENIINFI EKSYKSIFVL SPHFIQSEWC HYELYFAHHN

LFHEGSDNLI LILLAPIPQY SIPTNYHKLK TLMSRRTYLE WPTEKNKHGL FWANLRASIN

VKLVNQAEGT CYTQQ

>TLR1RN

MTKTQSTIFY CIVVLGLILI KIQLSEESEL IIKRPNANLT RVPKDLPLQT TTLDVSQNNI

SELQTSDILL LSKLRVFIMS YNRLQYLNIS VFKFNTELEY LDLSHNELRL ISCHATADLK

HLDLSFNAFD ALPICKEFGN LSQLQFLGLS GSQIQNSSVQ LIAHLNISKV LLVLGDTYGE

KEDPKCLQHI STETLHIVFP SKREFHFLLD MSVSTAISLE LSNIKCVLED KNCSYFLGTL

ERLRKTQRLS NLTLNNVDTT WNSFINILQL VWHTPVKSFS ISNVKLKGHF NFRRFHYSDT

SLRALSIHQV VTDVFSFPQS NIYSIFSNMN IQSFTVSGTR MVHMLCPDQI SPFLYLDFTD

NLLTDIVFED CRNLIRLKTL SLQKNQLKTL ENIILMSMEM TSLQKLDISQ NSLRYSDAGS

PCSWTQSLLV LNLSSNMLTD SVFRCLPPKV KVLDLHNNRI VSISKDVTHL QALQELNVAS

NFLTDLPGCG AFSSLSVLVI DHNSVSHPSS DFFQSCQNIR SITAGNNPFR CTCELREFVK

NIGQASREVV EGWPDSYRCD YPDSIKGTPL QDFHMSPLSC DTILLTVTIG ATLLLLAAIG

ASLCLYFDLP WYLRMLWQWT QTRHRARNIP LEELQRNLQF HAFVSYSGHD SAWVKNELLP

NLEKDDIRVC LHERNFVPGK SIVENIIHFI EKSYKSIFVL SPHFIQSEWC HYELYFAHHN

LFHEGSDNLI LILLEPIPQY SIPTNYHKLK TLMARRTYLE WPTEKSKHGL FWANLRASIN

VKLVNQAEAT CYTQQ

>TLR1EE

MTKTYSIVFH LIIIFMLIVK IRTLLSDGSD VLADRSNRTL IHIPKDLPPS TTILNVSHNY

ISELWASDIL SLSKLKILIM SHNRIQNLDI SVFRFNQELE YLDLSHNKLE TISCHSTANL

KHLDLSFNAF VFLPICKEFG NMSQLEFLGL SASQLQKSRL LSISHLHISK VLLVLGDSYG

EKEIPDSLQD LNTESLHIVF PLGKEFHFNL DVSISQAVSL ELSNIQYVLE DGGACSFQNA

LRKLQKNPRL SNLTLNNIDT TWNSFMMILQ LVWHTGVEYF SIKNVKLQGW FHPREFNYSD

TSLKSLTIHQ VVNNAYSLEQ NSIYKIFANM NIQHFTVSGT PMVHMLCPLQ TSPFLYLDFS

NNLLTDMIFK DCGNLTKLET LILQMNQLQE FTKIVYMTKK MKSLQLLDIS QNSLRIDENE

GNCSWTESLS SLNLSSNILT ESVFRCLPPR VKVLDLHSNR IRSIPRDVNN LEALQVLNVA

SNFLTNLPGC GAFSSLSALI IDYNSISSPS VDFFQSCQNI RSVKAGNNPF QCTCELREFV

QRMGQVSREV VEDWPGSYQC DYPESFKGTA LKDFHMSQLS CNTTLLIVTI VVIVLVLGST

TVMLCIYFDV LWYLRMMCHW TQTRQRARNT PLAELQRNLQ FHAFISYSEH DSAWVKSELL

PNLEKENIRI CLHERNFVPG KSIIENIINC IEKSYKSIFI LSPNFVQSEW CHYELYFAHH

NLFHKGSDNL ILILLEPIPQ YSIPNSYHKL KALMARRTYL EWPKEKRKHG LFWASLRVSI

NIKLTEQAKE VCHTQIQNIL TTSAF

>TLR1BT

MTKKNSSIFH FAIIFILILE IRTQLSDESE FLIDRSKRGL TYVPKNLSLE TTILDISYNY

ISELQMPDIL SLSKLKILII SHNRIQYLDL SVFKFNQELE YLDLSHNNLE KISCHPTLNL

KHLDLSFNPF DALPICQEFG NMSQLEFLGL SATQLQKSSV QSITHLHISK VLLVLGDTYG

EREDAESLQD LKTQSLHIVF PTGKEFHFIL DVSVGTTVSL ELSNIKCVLD DNGCPYFENV

LSKLQKNSRL SNLTLNNIEI TWNSFFTILQ LVWRTNIEYF SISNVKLQGY LDSRDFDYSD

TSLKALSIHK VVHDVFSLPQ GYVYKILSNM NIQHLTVSAA HMVHMVCPSQ ISPFLYLNFS

NNLLTDTVFI NCTNLANLKT LILQKNQLKE LVNIVHMTQE MKSLQQLDVS QNSLMYDESE

GNCPWARNLL SLNMSSNILT DSVFRCLPPQ IKVLDLHNNR IRSIPKDVTG LETLQELNLA

SNSLAHLPGC GIFSSLSILI IDYNSISNPS ADFFQSCQKI RSLKAGNNPF QCSCELRDFI

QSIGQVSSDV VEGWPESYKC DYPESYKGTP LKDFQVSELS CNTALLIVTI VVPGLVLAVA

VTVLCIYLDL PWYLRMVCQW TQTRRRARNV PLEELQRTLQ FHAFISYSGH DSAWVKNELI

PNLEKEDIRI CLHERNFVAG KSIVENIINC IEKSYKSIFV LSPNFVQSEW CHYELYFAHH

NLFHEGSDNL ILILLDPIPQ YSIPSSYHKL RALMAQRTYL EWPKEKSKHG LFWANLRASI

NIKLMEKAAE IH

>TLR1SS

MTKENLSIFH FAIIFILILE IRIQLSEESE VLVDRSKTGL THVPKDLSLE TTILDLSQNS

ISELQTSDIL SLSKLRVFII SHNRIQYLDV SVFKFNQELE YLDLSHNKLE KISCHPMLNL

KHLDLSFNAF DALPICQEFG SMFQLEFLGL SATQLQKSSV LPIAHLHIGK VLLVLGDSYG

EREDPESLQD LNTQSLHIVY PPGKEFHFML DVSVSTAVNL ELSNIRCVLD ANGCHHFQNV

LLKLQKNSKL SNLTLNNIET TWNSFITTLQ FVWHTSIEYF SISSVKLQGQ LDFRDFDYSD

TSLKALSLHQ VVSEVFSFPQ SYIYKIFSNM NIQYLTVSAT HMVHMVCPSQ ISPFLYLDFS

NNALTDMVFK NCANLANLNT LSLQMNQLKE LVNVIHMTKE MQSLQQLDVS QNTLRYDENE

GSCTWTGSLL SLNLSSNILT DSVFRCLPPR IKVLDLHNNR IRSIPKDVAH LEALQELNVA

SNSLAHLPGC GSFSSLSILI IDYNSISNPS ADFFQSCQKI RSLKAGNNPF QCTCELRDFI

QSLGQVSSDV VESWPDSYEC EYPESYKGTL LKDFRVSELS CNTALLIVTI GVTGLALALT

MTGLCVYFDL PWYLRMLCQW TQTRRRARNV PLEELQRTLQ FHAFISYSGH DSAWVKNELL

PNVEKEGIKI CLHERNFVPG KSIMENIINC IEKSYKSIFV LSPNFVQSEW CHYELYFAHH

NLFHEGSDNL ILILLDSIPQ YSIPSSYHKL KALMAQRTYL EWPKEKSKHG LFWANLRASI

NIKLMEKAEE ISYTQI

>TLR1CF

MMKTNPSIFQ FAIIFILILE IRIQLSEESD FLVNRSKAGL FHIPKDLSLK TTILDISQNY

ISELQTSDIL SLSKLRILIV SYNRIQYLDI SVFKFNQELE YLDLSHNELG RISCHPTVNL

KHLDLSFNAF DDLPICKEFG NMSQLEFLGL SATQLQKSSM LPIASLHIRK VLLVLGDTYG

KKEDPESLQK LNTESLHIVF PIRKEFSFTL DVSVSTAVSL ELSNIKCVPD GHGWSYFQNV

LSKLQKNSRL SSLTLNNIET TWNFFIMLLQ LVWHTSIEYF SISNVKLQGY PDFRDFDYSD

TSLKALSIHQ VVSNAFNLPQ SYIYKIFSNM NIQNFTVSGT HMVHMVCPSQ ISPFLHLDFS

NNLLTDIVFK NCRNLIKLET LSLQMNQLKE LASIAQMTNE MKSLQQLDIS QNSLRYDENE

GNCSWTRSLL SLNMSSNILT DSVFRCLPPK VKVLDLHDNR IRSIPKPIMK LEDLQELNVA

SNSLAHFPDC GTFNRLSVLI IDSNSISNPS ADFLQSCHNI RSISAGNNPF QCTCELREFV

QSLGQVASKV VEGWPDSYKC DSPENYKGTL LKDFHVSPLS CNTTLLLVTI GVAVLVFTVT

VTALCIYFDL PWYLRMVFQW TQTRRRARNT PLENLQRTIQ FHAFISYSGH DSAWVKSELL

PNLEKEELRI CLHERNFIPG KSIVENIINC IEKSYKSIFV LSPNFVQSEW CHYELYFAHH

NLFHEGSNNL ILILLEPIPQ YSIPSSYHKL KNLMAQRTYL EWPKEKSKHG LFWANLRASI

NIKLREQAKK

>TLR6HS

MTKDKEPIVK SFHFVCLMII IVGTRIQFSD GNEFAVDKSK RGLIHVPKDL PLKTKVLDMS

QNYIAELQVS DMSFLSELTV LRLSHNRIQL LDLSVFKFNQ DLEYLDLSHN QLQKISCHPI

VSFRHLDLSF NDFKALPICK EFGNLSQLNF LGLSAMKLQK LDLLPIAHLH LSYILLDLRN

YYIKENETES LQILNAKTLH LVFHPTSLFA IQVNISVNTL GCLQLTNIKL NDDNCQVFIK

FLSELTRGPT LLNFTLNHIE TTWKCLVRVF QFLWPKPVEY LNIYNLTIIE SIREEDFTYS

KTTLKALTIE HITNQVFLFS QTALYTVFSE MNIMMLTISD TPFIHMLCPH APSTFKFLNF

TQNVFTDSIF EKCSTLVKLE TLILQKNGLK DLFKVGLMTK DMPSLEILDV SWNSLESGRH

KENCTWVESI VVLNLSSNML TDSVFRCLPP RIKVLDLHSN KIKSVPKQVV KLEALQELNV

AFNSLTDLPG CGSFSSLSVL IIDHNSVSHP SADFFQSCQK MRSIKAGDNP FQCTCELREF

VKNIDQVSSE VLEGWPDSYK CDYPESYRGS PLKDFHMSEL SCNITLLIVT IGATMLVLAV

TVTSLCIYLD LPWYLRMVCQ WTQTRRRARN IPLEELQRNL QFHAFISYSE HDSAWVKSEL

VPYLEKEDIQ ICLHERNFVP GKSIVENIIN CIEKSYKSIF VLSPNFVQSE WCHYELYFAH

HNLFHEGSNN LILILLEPIP QNSIPNKYHK LKALMTQRTY LQWPKEKSKR GLFWANIRAA

FNMKLTLVTE NNDVKS

>TLR6PT

MTKDKEPIVK SFHFVCLMII IVGTRIHFSD GNEFAVDKSK RGLIHVPKDL PLKTKVLDMS

QNYIAELQVS DMSFLSELKV LRLSHNRIQL LDLSVFKFNQ DLEYLDLSHN QLQKISCHPI

VSFRHLDLSF NDFKALPICK EFGNLSQLNF LGLSAMKLQK LDLLPIAHLH LSYILLDLRN

YYIKENETES LQILNAKTLH LVFHPTSLFA IQVNISVNTL GCLQLTNIKL NDDNCQVFIK

FLSELTRGPT LLNFTLNHIE TTWKCLVRVF QFLWPKPVEY LNIYNLTIIE SIHEEEFTYS

KTTLKALKIE HITNKVFLFS QTALYTVFSE MNIMMLTISD TPFIHMLCPH APSTFKFLNF

TQNVFTDSIF EKCSTLVKLE TLILQKNGLK DLFKVGLMTK DMPSLEILDV SWNSLESGRH

KENCTWVESI VVLNLSSNML TDSVFRCLPP RIKVLDLHSN KIKSVPKQVI KLKALQELNV

AFNSLTDLPG CGSFSSLSVL IIDHNSVSHP SADFFQSCQK MRSIKAGDNP FQCTCELRQF

VKSIDQVSSE VLEGWPDSYK CDYPESYRGT PLKDFHMSEL SCNITLLIIT IGATMLVLAV

TVTSLCIYLD LPWYLRMVCQ WTQTRRRARN IPLEELQRNL QFHAFISYSE HDSAWVKTEL

VPYLEKEDIQ ICLHERNFVP GKSIVENIIN CIEKSYKSIF VLSPNFVQSE WCHYELYFAH

HNLFHEGSNN LILILLEPIP QNSIPNKYHK LKALMMQRTY LQWPKEKSKR GLFWANIRAA

FNMKLTLVTE NNDVKS

>TLR6Ma

MTKDKEPVVK SFHFVCLMII IVGTRIQFSD GSEFAVDKSK RGLTHVPKDL PPKTKVLDMS

HNYIAELQVS DISFLSELKV LRLSHNKIQL LDLSVFKFNQ DLEYLDLSHN QLQKISCHPI

MSFRHLDLSF NDFEALPICK QFGNLSQLNF LGLSAMKLQK LDLLPIAHLH LSYILLDLRN

YYIKENETES LQILNAKTLH LVFHPTSLFS IQVNISVNTL GCLQLTNIKL NDDNCQVFIK

FLLELTRGPT LLNFTLNHIE TTWKCLVRVF QFLWPKPVEY LNIYNLTIIE SIHEEDFTYS

KTTLKALKIE HITNQVFIFS QTALYTVFSE MNIMMLTISD TPFIHMLCPR APSTFKFLNF

TQNVFTDSIF EKCSTLVKLE TLILQKNGLK DLFKVGLMTK DMPSLEILDV SWNSLESGRH

RENCTWVESI VVLNLSSNIL TDSVFRCLPP RIKVLDLHNN KIKSIPKQVV KLEALQELNV

AFNSLTDLPG CGSFSSLSVL IIDHNSVSHP SADFFQSCQK MRSIKAGNNP FQCTCELREF

VKNIEQVSSE VVEGWPDSYK CDYPESYRGT PLKDFHMSEL SCNITLLIIT IGATMLVLAV

TVTFLCIYLD LPWYLRMVCQ WTQTRHRARN VPLEELQRNL QFHAFISYSE HDSAWVKNEL

VPYLEKEGMQ VCLHERNFVP GKSIVENIIN CIEKSYKSIF VLSPNFVQSE WCHYELYFAH

HNLFHEGSNN LILILLEPIP QNSIPNKYHK LRALMTQRTY LQWPKEKSKR GLFWANIRAT

FNVKLTLVTE NNDVKS

>TLR6MM

MSQDRKPIVG SFHFVCALAL IVGSMTPFSN ELESMVDYSN RNLTHVPKDL PPRTKALSLS

QNSISELRMP DISFLSELRV LRLSHNRIRS LDFHVFLFNQ DLEYLDVSHN RLQNISCCPM

ASLRHLDLSF NDFDVLPVCK EFGNLTKLTF LGLSAAKFRQ LDLLPVAHLH LSCILLDLVS

YHIKGGETES LQIPNTTVLH LVFHPNSLFS VQVNMSVNAL GHLQLSNIKL NDENCQRLMT

FLSELTRGPT LLNVTLQHIE TTWKCSVKLF QFFWPRPVEY LNIYNLTITE RIDREEFTYS

ETALKSLMIE HVKNQVFLFS KEALYSVFAE MNIKMLSISD TPFIHMVCPP SPSSFTFLNF

TQNVFTDSVF QGCSTLKRLQ TLILQRNGLK NFFKVALMTK NMSSLETLDV SLNSLNSHAY

DRTCAWAESI LVLNLSSNML TGSVFRCLPP KVKVLDLHNN RIMSIPKDVT HLQALQELNV

ASNSLTDLPG CGAFSSLSVL VIDHNSVSHP SEDFFQSCQN IRSLTAGNNP FQCTCELRDF

VKNIGWVARE VVEGWPDSYR CDYPESSKGT ALRDFHMSPL SCDTVLLTVT IGATMLVLAV

TGAFLCLYFD LPWYVRMLCQ WTQTRHRARH IPLEELQRNL QFHAFVSYSE HDSAWVKNEL

LPNLEKDDIR VCLHERNFVP GKSIVENIIN FIEKSYKAIF VLSPHFIQSE WCHYELYFAH

HNLFHEGSDN LILILLEPIL QNNIPSRYHK LRALMAQRTY LEWPTEKGKR GLFWANLRAS

FIMKLALVNE DDVKT

>TLR6RN

MSQDREPIVE SFHFVCTLAL IVGSMTQFSD EFESVVDYSN KNLTHVPKDL SPSTKSLSLS

QNSISDLQMS DISFLSELRV LRLSHNRIRR LDFGVFLLNR DLEYLDVSHN QLQNISCCPM

VNLKHLDLSF NDFEVLPVYK EFGNLMKLSF LGLSAAKFRQ LDLLPISHLH LSCVLLDLVN

YQIKDGETES LQVPNTNVLH LVFHPNSLFS VQVNISVNAL GCLQLSNIKL NDKNCQSLII

FLSELTRGPT LLNLTLQHIE TNWKCFVRLL QFLWPRPVEY LNIYNLTITE SISRETFIYV

ETVLKSLKIE HVTNQVFLFV KDALYSVFAE MNIRMLTLSD TPFIHMVCPE FPSTFAFLNF

TQNVFTDSIF QGCSTLKRLE TLILQRNGLK NLFKVALMTK TMSSLETLDV SLNSLNSHVY

DRTCAWAESI RVLNLSSNVL SDSVFRCLPP KVKVLDLHNN RIVSIPKDVT HLQALQELNV

ASNFLTDLPG CGAFSSLSVL VIDHNSVSHP SSDFFQSCQN IRSITAGNNP FRCTCELREF

VKNIGQASRE VVEGWPDSYR CDYPDSIKGT PLQDFHMSPL SCDTILLTVT IGATLLLLAA

IGASLCLYFD LPWYLRMLWQ WTQTRHRARN IPLEELQRNL QFHAFVSYSE HDSAWVKNEL

LPNLEKDDIR VCLHERNFVP GKSIVENIIH FIEKSYKSIF VLSPHFIQSE WCHYELYFAH

HNLFHEGSDN LILILLEPIQ QNNIPSRYHK LRALMAQRTY LEWPIEKGKR GLFWANLRAS

FIMKLALVNE NDVKT

>TLR6EE

MTRDKESTMR SFAYTVIVTV GIVIQSSDEN EFTVDLSKRG LTHIPRDLPS QTEVLDMSQN

NVSELHLSDM SLISRLKVLR LSHNRIQCLD FSVFRFNQDL EYLDLSHNQL KKLSCHAIQS

LQHLDLSFND FDSLPICKEF GNLTQLNFLG LSTSKLQQLD LLPVSHLHVD SILLDLQGYS

MKGNETGSLQ IFNTKKLHFV FHPNDLFSVL VNISVNMIEC LQLTNIKLND DNCQVLINFL

SEPLIRGPNL LNLTLDHVET TWKCLVRVFQ SLWSKPIEYL NIYNLTVVER IDEEEFTYSK

TSLKALKIEH ITNRVFLFSQ TALYTVFSEM NIMMLTISEA PLIHMLCPQA PSMFKFLNFT

HNVFTDSIFQ NCSTLGRLET LILQKNELKE LFKVGLMTKD MQSLEILDVS WNSLNYDRYD

GICTWAQSIV MLNLSSNILT ESVFRCLPPR VKVLDLHSNR IRSIPRDVNN LEALQVLNVA

SNFLTNLPGC GAFSSLSALI IDYNSISSPS VDFFQSCQNI RSVKAGNNPF QCTCELREFV

QRMGQVSREV VENWPGSYQC DYPESFKGTA LKDFHMSQLS CNTTLLIVTI VVIVLVLGST

TVMLCIYFDV LWYLRMMCHW TQTRQRARNT PLAELQRNLQ FHAFISYSEH DSAWVKNELV

PCLEKENIRI CLHERNFVPG KSIIENIINC IEKSYKSIFV LSPNFVQSEW CHYELYFAHH

NLFHEGSDNL ILILLEPIPQ NNIPSKYHKL KALMTQRTYL EWPKEKSKHG LFWANITAAF

HMKLTLVNEN DAET

>TLR6BT

MIKDKESPIR SCHFVYIVAL VFGTIIQFSD ESEFVVDMSK TSLIHVPKDL PPKTKVLDLS

QNNISELHLS DISFLSGLRV LRLSHNRIQG LDISIFKFNH DLEYLDLSHN QLQKISCHPI

TTTLKHLDLS FNDFDALPIC KEFGNLTQLN FLGLSATKLQ QLDLLPIAHL HLSCILLDLE

DYMKENKKES LQILNTKKLH LVFHPNSFFS VQVDISANSL GCLQLTNIKL NDYNCQVLLK

FLSGLTGGPT LLNFTLNHME TTWKCLVKVF QFLWPKPIEY LNIYNLTIVE SIDEEVFTYY

KTTLKALKIE HITNKVFIFS QTALYTVFSE MNILMLTISD TRFIHMLCPQ EPSTFKFLNF

TQNSFTDSVF QNCDTLARLE TLILQKNELK DLFKTSLMTK DMLSLETLDV SWNSLEYDRS

NGNCSWVGSI VVLNLSSNAL TDSVFRCLPP RIKVLDLHNN RIRSIPKDVT GLETLQELNL

ASNSLAHLPG CGIFSSLSIL IIDYNSISNP SADFFQSCQK IRSLKVGNNP FQCSCELRDF

IQSVGQVSSD VVEGWPESYK CDYPESYKGT PLKDFQVSEL SCNTALLIVT IVVPGLVLAV

AVTVLCIYLD LPWYLRMVCQ WTQTRRRARN VPLEELQRTL QFHAFISYSE HDSAWVKNEL

IPNLEKEDIR ICLHERNFVA GKSIVENIIN CIEKSYKSIF VLSPNFVQSE WCHYELYFAH

HNLFHEGSNN LILILLEPIP QNTIPDRYHK LRALMAQRTY LEWPKEKNKH GLFWANIRAA

FNIKLRLVTE NDDVKG-

>TLR6SS

MTKDKKPTVI SLHSVYVMTL VWGTLIQFSE ESEFVVDKSK IGLTRVPKDL PPQTKVLDVS

QNFITELHLS DISFLSQLTV LRLSQNRMQC LDISVFKFNQ DLEYLDLSHN QLQTILCHPI

TSLKHLDLSF NDFEALPICK EFGNLTQLNF LGLSATKLQQ LDLLPIAHLH LSCILLDLER

YYMKENEKES LQILNTEKLH LVFHPNSFFS VQVNISVKSV GCLQLANIKL GDDNCQVFIT

FLLELTQGPT LLNFTLNHVE TTWKCLVGIF QFLWPKPVEY LSIYNLTIVE SIDEEDFIYY

ETTLKGVKIE HITKRVFIFS QTALYRVFSD MNIRMLTIAD THFIHMLCPQ VPSTFNFLNF

TQNVFTDSVF QNCKTLARLE TLILQKNKLE DLFKISLMTK DMLSLEILDV SSNSLEYDRH

GENCTWVGSI VVLNLSSNIL TDSVFRCLPP RIKVLDLHSN RIRSIPKDVA HLEALQELNV

ASNSLAHLPG CGSFSSLSIL SIDYNSISNP SADFFQSCQK IRSLKAGNNP FQCTCELRDF

IQSLGQVSSD VVESWPDSYE CEYPESYKGT LLKDFRVSEL SCNTALLIVT IGVTGLALAL

TMTGLCVYFD LPWYLRMLCQ WTQTRRRARN VPLEELQRTL QFHAFISYSE HDSAWVKNEL

VPCLEKEGIK ICLHERNFVP GKSIMENIIN CIEKSYKSIF VLSPNFVQSE WCHYELYFAH

HNLFHEGSDN LILILLDPIP QNSIPGKYHK LKALMAQRTY LEWPKEKSKH GPFWANIRAA

FNIKLKLVAE EDDVKT

>TLR6CF

MIKDKDSITG SFHFVYIVTL IVGTIIQFSD ESEFTVDMSN MNLTHVPEDL PPKTKILDMS

QNNISELHLS DMSYLSGLKI LRISHNRIWW LDFSIFKFNQ DLEYLDLSYN QLRNMSCHLI

RSLKHLDLSF NDFHVLPICK EFGNLTQLQF LGLSATKLRQ LDLLPIAHLH LSYILLDLQG

YYAKESEKGS LQILDTKTLH LVFHPNQLFS VQANMLVNNL GCLQLTNIKL NNDNCQVLIQ

FLSELTRGPT LLNFTLQHVK TTWKCLVRIF KFLWPKPVQY LNIYNLTIVE SINKEYIHYP

KTALKALTIE HVKNEVFLFS QTALYTIFSE MNIMMLTISD TPFIHMLCPP PSNTFKFLNF

TQNVFTDSVF QSCSHLVRLE TLILRKNKLK DLYKVGLMTK HMTSLEILDV SVNSLEYDRY

DGNCTWVGSI VVLNLSSNIL TDSVFRCLPP KVKVLDLHDN RIRSIPKPIM KLEDLQELNV

ASNSLAHFPD CGTFNRLSVL IIDSNSISNP SADFLQSCHN IRSMSAGNNP FQCTCELREF

VQSLGQVASK VVEGWPDSYK CDSPENYKGT LLKDFHVSPL SCNTTLLLVT IGVAVLVFTV

TVTALCIYFD LPWYLRMVFQ WTQTRRRARN TPLEELQRTI QFHAFISYSE HDSAWVKNEL

VPCLEKEELR ICLHERNFIP GKSIVENIIN CIEKSYKSIF VLSPNFVQSE WCHYELYFAH

HNLFHEGSNN LILILLEPIP QNCIPSKYHK LRALMTQRTY LEWPKEKSKH GLFWANIRAA

FNMKLTLIAE NNNAEAS
